# A new tyrannosaur with evidence for anagenesis and crocodile-like facial sensory system

**DOI:** 10.1038/srep44942

**Published:** 2017-03-30

**Authors:** Thomas D. Carr, David J. Varricchio, Jayc C. Sedlmayr, Eric M. Roberts, Jason R. Moore

**Affiliations:** 1 Carthage College, 2001 Alford Park Drive, Kenosha, WI 53140, USA; 2 Department of Earth Sciences, Montana State University, P.O. Box 173480, Bozeman, MT 59717-3480, USA; 3 Louisiana State University Health Sciences Centre - School of Medicine, 1901 Perdido Street, New Orleans, LA 70112, USA; 4 Geosciences, College of Science and Engineering, James Cook University, Townsville, QLD 4811, Australia; 5 Honors College, University of New Mexico, Albuquerque, NM 87131, USA.

## Abstract

A new species of tyrannosaurid from the upper Two Medicine Formation of Montana supports the presence of a Laramidian anagenetic (ancestor-descendant) lineage of Late Cretaceous tyrannosaurids. In concert with other anagenetic lineages of dinosaurs from the same time and place, this suggests that anagenesis could have been a widespread mechanism generating species diversity amongst dinosaurs, and perhaps beyond. We studied the excellent fossil record of the tyrannosaurid to test that hypothesis. Phylogenetic analysis places this new taxon as the sister species to *Daspletosaurus torosus*. However, given their close phylogenetic relationship, geographic proximity, and temporal succession, where *D. torosus* (~76.7–75.2 Ma) precedes the younger new species (~75.1–74.4 Ma), we argue that the two forms most likely represent a single anagenetic lineage. *Daspletosaurus* was an important apex predator in the late Campanian dinosaur faunas of Laramidia; its absence from later units indicates it was extinct before *Tyrannosaurus rex* dispersed into Laramidia from Asia. In addition to its evolutionary implications, the texture of the facial bones of the new taxon, and other derived tyrannosauroids, indicates a scaly integument with high tactile sensitivity. Most significantly, the lower jaw shows evidence for neurovasculature that is also seen in birds.

Tyrannosaurinae is a diverse clade of advanced theropods from the Late Cretaceous of Asia and the American West that culminated in the giant *T. rex* at the end of the Mesozoic^1^,^2^,^3^,^4^. The clade has a high diversity in body size and skull shape, including medium-sized taxa with long and low skulls^5^, large taxa with short snouts^2^, and a dwarf species from the Arctic Circle^6^. Tyrannosaurines are geographically widespread; fossils have been collected in China and Mongolia, and throughout western North America, from Alaska to Texas. The evolutionary success of Tyrannosaurinae is also reflected in its 14 million-year duration, which includes the geologically oldest species of tyrannosaurid^4^.

We report here the discovery of a new large tyrannosaurine (Figs 1–4) from the Upper Cretaceous of Montana. Phylogenetic analysis places this taxon as the sister species of *Daspletosaurus torosus* (Fig. 2). The new species adds to the number of late Campanian tyrannosaurines and shows that this clade was a dominant component of the dinosaur faunas of the American West shortly after the emplacement of the Western Interior Seaway, in contrast to the depauperate, and geographically and temporally restricted *Albertosaurus* lineage that makes up the sister lineage of Tyrannosaurinae. In terms of its geological age and excellently preserved fossils, the new taxon is important regarding tyrannosaur evolution and appearance.

Tyrannosaurs, the larger group of giant predatory dinosaurs of the Late Cretaceous from Asia and North America, are notable in terms of diversity and evolution thanks to many discoveries of new species over the past decade^2^,^3^,^4^,^7^,^8^,^9^,^10^. With an excellent fossil record, tyrannosaurs stand as exemplar fossil organisms in studies of dinosaur biomechanics, development, anatomy, evolution, cognition, and paleoecology^3^,^7^. Further, their well-resolved phylogeny and excellent stratigraphic record provides the rare opportunity to investigate the evolutionary processes (i.e., speciation modes) behind their numerical diversity and extreme morphologies^2^,^5^,^7^,^10^,^11^.

Several extinct clades with stratigraphically continuous fossil records, including invertebrates^12^, sticklebacks^13^, and mammals^14^ exhibit anagenesis, evolution within a single lineage. Among living species, anagenesis has occurred in cyprinid teleosts^15^ and it has produced genetic diversity among oceanic island plants^16^,^17^. Anagenesis gained currency in studies of dinosaur evolution with Horner *et al*.’s^1^ landmark study that found evidence for phyletic evolution in four lineages of dinosaurs, including tyrannosaurs, from the Late Cretaceous of the American West.

Renewed interest in anagenesis among dinosaur workers stems from the further discovery of stratigraphically stacked sequences of ornithischian dinosaurs, specifically hadrosaurids and ceratopsians^18^,^19^ and coincides with the realization among paleontologists and neontologists that the branching patterns of phylogenies do not necessarily reflect the actual processes that produced organismal diversity^20^,^21^,^22^,^23^. For example, anagenesis is a linear process that is obscured when the continuum of change is atomized into multiple subunits (species) and are arranged into a branching pattern on a phylogenetic tree^12^,^22^.

Currently, it remains unclear if anagenesis is a frequent mode of speciation in dinosaur evolution. Studies of other organisms suggest that it is not^20^. For example, cladogenesis (splitting or budding of an ancestor into daughter species) is considered to be the most frequent speciation mode among fossil invertebrates, and perhaps across metazoans in general^12^,^15^,^20^.

For paleontologists, the best evidence for anagenesis requires dense stratigraphic sampling and precise radiometric dating, temporally successive specimens of close phylogenetic relationship, clear stratigraphic data for specimens, and a high sample size that includes growth series^1^,^18^,^19^,^20^,^24^,^25^. Tyrannosaurs represent one of the few dinosaur clades that meet these criteria^7^,^26^,^27^,^28^. Here we present a test of Horner *et al*.’s^1^ case for anagenesis among Late Cretaceous North American tyrannosaurs that meet all of those criteria.

Tyrannosaurs in the flesh are ubiquitous across all media, from museum galleries to Hollywood blockbusters, arguably making them the most iconic dinosaurs in modern culture. As ambassadors of Natural History, it is ironic that the life appearance of the tyrannosaur face has not been rigorously studied, despite the availability of well-preserved fossils, published data on their closest living relatives (birds, crocodylians), and modern comparative methods^29^,^30^. The question of facial integument in theropods (meat-eating dinosaurs) is critical to understanding the evolutionary transition from nonbeak to beak along the line to birds, since beaks are specialized epidermal structures^31^.

Tyrannosaurs are central to this question: they are advanced, bird-like theropods with large skulls covered in a variety of textures that correspond to identifiable, overlying soft tissues^29^. We apply the information known about textures and their causal soft tissues to deduce the life appearance of the integumentary covering of tyrannosaurs^29^; these results can then be placed into the framework of the evolutionary transition from nonavian theropods to birds. Theropoda Marsh, 1881

Tyrannosaurinae Matthew and Brown, 1922 (sensu Sereno *et al*., 2005)

*Daspletosaurus* Russell, 1970

*Daspletosaurus*. All species more closely related to *Daspletosaurus torosus* than to *Tyrannosaurus rex*.

*D. horneri* sp. nov.

## Etymology

*Horneri*, Latinized form of Horner, in honor of Jack Horner, in recognition of his successful field program in the Two Medicine Formation that has recovered many new species of dinosaurs that are critical for our understanding of the palaeobiology of dinosaurs in Laramidia, support in the preparation and curation of these specimens, and to acknowledge that his mentoring efforts have launched many professional scientific careers.

## Holotype

**Figure 1:**
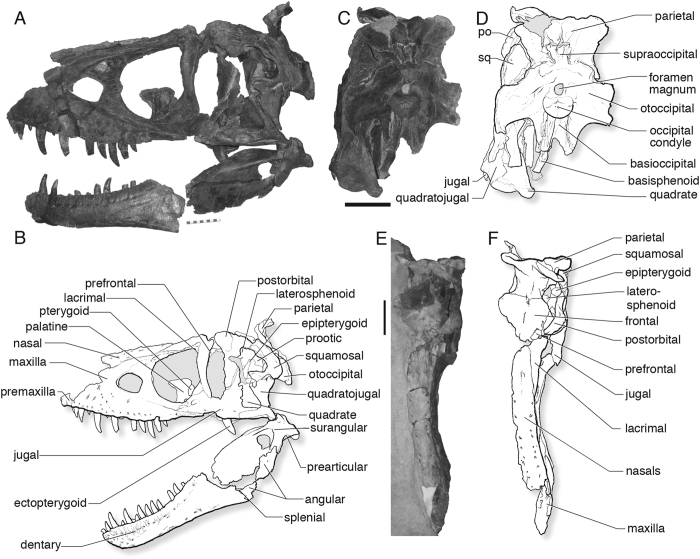
Skull and jaws of the holotype (MOR 590) of *Daspletosaurus horneri* sp. nov.; (**A**) photograph and, (**B**) labeled line drawing of skull and jaws in left lateral view; (**C**) photograph and, (**D**) labeled line drawing of occiput and suspensorium in caudal view; (**E**) photograph and, (**F**) labeled line drawing of skull in dorsal view. Scale bars equal 10 cm. Abbreviations: MOR, Museum of the Rockies.

MOR (Museum of the Rockies, Bozeman) 590, a complete skull, partial pectoral limb, and nearly complete hindlimb (Fig. 1, see Supplementary Tables S1, S2, S3).

## Paratypes

MOR 1130, an incomplete skull, partial axial series, and partial pelvic girdle and hindlimb. MOR 553S/7.19.0.97, a nearly complete dentary of a small juvenile, based on four features shared with the holotype and the other paratype: 17 dental alveoli, first three alveoli form a rostromedially extending arcade, laterally bowed dentary, rostroventral corner of bone is below the level of the septum between alveoli three and four.

## Referred material

AMNH FARB (American Museum of Natural History, Fossil Amphibians, Reptiles, and Birds, New York) 5477, a maxilla, partial postorbital, and parietal; MOR 3068, a partial mandibular ramus; MOR 553D.9.19.91, left ectopterygoid; MOR 553E.7.6.91.196, right ectopterygoid.

## Horizon and localities

Glacier County (Co.), Lewis and Clark Co., and Teton Co., Montana, USA; Upper Cretaceous upper Two Medicine Formation.

## Stratigraphic Distribution

The type and paratype specimens all come from the upper portion of the Two Medicine Formation^32^,^33^ (TMF); MOR 590 occurs 65 m below a dated bentonite horizon (TM-4)^7^, and the MOR 553 specimens sit at least 10 m above this same bentonite. TM-4 occurs 480 m above the base of the ~545 m–thick TMF^32^, and recalibration of legacy standards on ^40^Ar/^39^Ar ages from the TMF^32^ indicates that the TM-4 tuff is older than previously considered, yielding a recalibrated age of 75.03 Ma +/− 0.72 Ma^34^. The fourth skeleton (MOR 1130) comes from within the disturbed belt on the eastern flank of the Rocky Mountains where folding and faulting make determining exact stratigraphic positions difficult. However, the MOR 1130 specimen sits 5.9 m above a newly dated bentonite, reported here to be 74.38 +/− 0.72 Ma (U-Pb zircon weighted mean age (1 σ; MSWD = 0.55); see Supplementary Fig. S1, Discussion S1, Table S4; for bentonite locality information see ref. 35), indicating that it is slightly younger than the other specimens.

## Diagnosis

Can be distinguished from all other derived tyrannosauroids, including *Daspletosaurus torosus*, by the presence of: a wide dental arcade at the front of the snout, where the maxillary and dentary tooth rows extend distinctly rostromedially and the first interdental plate of the maxilla is narrow, which resembles those of the premaxilla where the tooth row is mediolaterally oriented; dentary distinctly bowed (convex) laterally; promaxillary sinus stopping between alveoli 3 and 4, as observed in medial view of the completely prepared pneumatic chamber; rostral end of the choana on the maxilla above alveolus 7; inflated dorsal surface of the lacrimal not reaching the medial edge of the bone; medial pneumatic recess of the lacrimal tall and narrow slot; concave upper half of orbital margin of the lacrimal; entire circumference of the pneumatic recess of the squamosal is undercut and clearly defined; sinuous rostral edge in dorsal view of the dorsotemporal fossa on the frontal; joint surface for the squamosal on the parietal covers the base of the caudolateral process; and the tympanic ridge extends onto the prootic.

Several autapomorphies were obtained by the cladistic analysis; autapomorphies were not included in the data matrix, but several characters were optimized on the terminal branch of *D. horneri*. These include a pneumatic foramen penetrating the lateral surface of the quadratojugal, shallow notch between the basal tubera, short epipophyses of the anterior cervicals, and the humerus is ~34% the length of the femur (for further comparisons see Supplementary Discussion S2 and Supplementary Fig. S2).

## Previous work

The so-called Two Medicine tyrannosaurine has been included in several phylogenetic analyses^2^,^3^,^4^,^8^,^11^,^36^, with scorings based primarily on MOR 590. It has universally been recovered as a derived tyrannosaurine, but its specific relationships are unresolved, where it has been recovered as the sister species of *D. torosus*^2^,^3^,^8^,^36^ or closer to *T. rex*^4^,^36^. Description of the taxon has been limited to brief summaries of salient features (e.g., form of lacrimal horn) that distinguish it from *D. torosus* and to differentiate *Daspletosaurus* from other genera of tyrannosaurs^1^,^37^, but an extensive description of the taxon has not been made.

## Description and comparisons

The *D. horneri* holoype is estimated to be ~9.0 m in total length and 2.2 m tall at the acetabulum (for additional measurements, see Supplementary Tables S1, S2 and S3). *D. horneri* differs from its sister species in that *D. torosus* has a rostral ramus of the lacrimal that is longer than the ventral ramus, indicating that *D. horneri* has a taller skull. The antorbital fossa of the lacrimal is separated by a deep concavity from the ventral ramus of the bone in *D. torosus*, whereas these surfaces are confluent in *D. horneri*. In *D. torosus*, the coronoid region of the surangular faces equally dorsally and laterally, whereas it faces more laterally than dorsally in *D. horneri*.

**Figure 2:**
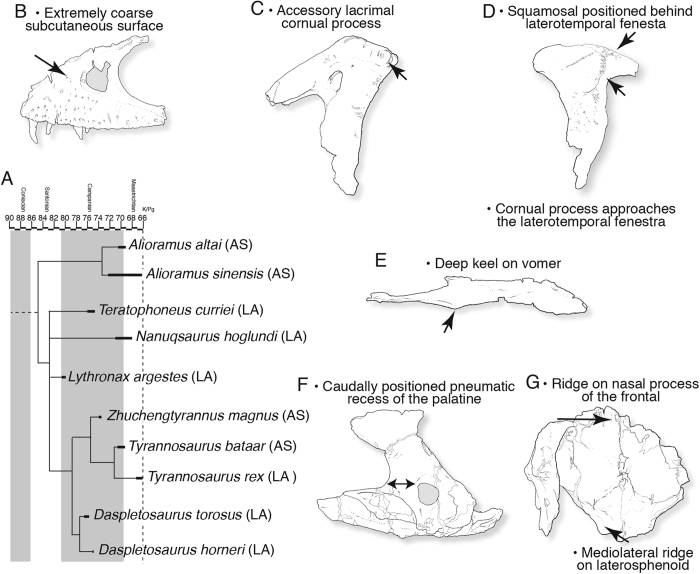
Phylogenetic position and synapomorphies of *Daspletosaurus*, based on parsimony analysis. (**A**) Phylogenetic relationships of tyrannosaurines calibrated to geological time. Full consensus trees in Extended Data. Synapomorphies of the *Daspletosaurus* lineage from: (**B**) maxilla of MOR 1130; (**C**) lacrimal of MOR 1130; (**D**) postorbital of CMN 11594; (**E**) vomer of MOR 590; (**F**) palatine of MOR 1130; and (**G**) frontoparietal complex of MOR 590. Abbreviations: AMNH FARB, American Museum of Natural History, Fossil Amphibians, Reptiles, and Birds; As, Asia CMN, Canadian Museum of Nature; K/Pg, Cretaceous-Paleogene; LA, Laramidia; MOR, Museum of the Rockies.

*Daspletosaurus* is one of the best-supported tyrannosaurid clades, distinguished by a suite of unique, mostly cranial features (Fig. 2) that suggests a relatively long evolutionary history. In particular, the cornual processes (‘horns’) of *Daspletosaurus* are enhanced: a new, secondary horn extends from the side of the large, triangular primary lacrimal horn that is expressed by most tyrannosaurids; and the postorbital horn, which nearly spans the rostrocaudal width of the postorbital bar, is the largest seen among tyrannosaurids. The primary cornual process of the lacrimal differs between the two *Daspletosaurus* species, being taller in *D. torosus* (ratio height of process to maximum height of rostral ramus: 69%) than in *D. horneri* (ratio: 53%).

## Results

### Phylogeny

The phylogenetic analysis (see Supplementary phylogenetic character list, Tables S5 and S6) recovered 18 most parsimonious trees, each having a tree length of 802 steps, a CI of 0.56, an HI of 0.44, and an RC of 0.45. The topology conforms to that of earlier works^3^,^11^, but the strict consensus tree and 50% majority rule tree shows that *Aviatyrannis* and Proceratosauridae and the lineage leading to more derived tyrannosauroids form an unresolved trichotomy (Fig. 2A; see Supplementary Fig. S3). Also, the tyrannosaurines *Lythronax, Teratophoneus*, and *Nanuqsaurus* form an unresolved trichotomy. *Alioramus* and *Qianzhousaurus* are recovered as sister species, and given that relationship we regard the genus *Qianzhousaurus* as a junior synonym of *Alioramus* (Fig. 2A). We follow the taxonomic practice in regarding sister species as congeneric, so this renaming does not constitute a phylogenetic rearrangement of alioramins. This convention is followed elsewhere in the tree (e.g., *Albertosaurus, Daspletosaurus, Tyrannosaurus*). Our results recover *Timurlengia* as more derived than *Xiongguanlong* and as the sister species of a new taxon from the Iren Dabasu Formation. The latter taxon (under study by TDC) is based on a partial individual (AMNH FARB 6556) that includes several teeth (premaxillary, lateral) and skull bones (lacrimal, jugal, pterygoid, ectopterygoid, quadratojugal) that was collected in 1923 under the auspices of the AMNH. The presence of D-shaped premaxillary teeth and the presence of a jugal pneumatic recess with a secondary fossa identifies it as a derived tyrannosauroid. The absence of hindlimb bones prevents comparison with the lectotype of *Alectrosaurus olseni*, and so it was treated as a separate taxon in our analysis (for a complete list of unambiguously resolved synapomorphies see Supplementary synapomorphy list).

Importantly, *Daspletosaurus horneri* is recovered as the sister species of *D. torosus*; this relationship is supported by 11 unambiguously optimized synapomorphies (Fig. 2B–G), including the presence of a coarse subcutaneous surface of the maxilla, an accessory cornual process on the lacrimal, a partly concealed maxillary process of the lacrimal, a cornual process of the postorbital that closely approaches the laterotemporal fenestra, a rostral tip of the squamosal that stops caudal to the rostral margin of the laterotemporal fenestra, a ridge along the nasal process of the frontal, a deep ventral keel on the vomer, a caudal pneumatic recess of the palatine that is positioned caudal to the rostral margin of the dorsal process, a distinct mediolaterally oriented ridge on the dorsum of the laterosphenoid that extends toward the medial edge of the dorsotemporal fenestra, a ‘chin’ of the dentary (where the rostral and ventral margins of the dentary join) that is positioned below the third dentary tooth, and there are more than 13 maxillary teeth.

### Historical biogeography

The pattern of historical biogeography indicated by our parsimony analysis is consistent with the hypothesis of Brusatte and Carr^11^, where North American taxa are not divided into northern and southern subclades^4^. Our topology shows an Asian diversification intermediate-grade tyrannosauroids (*Xiongguanlong baimoensis, Timurlengia euotica*, Iren Dabasu taxon) during the mid- and early Late Cretaceous that preceded a dispersal event to North America, which was followed by subsequent and frequent exchange between the landmasses throughout the Late Cretaceous.

### Ontogeny

**Figure 3:**
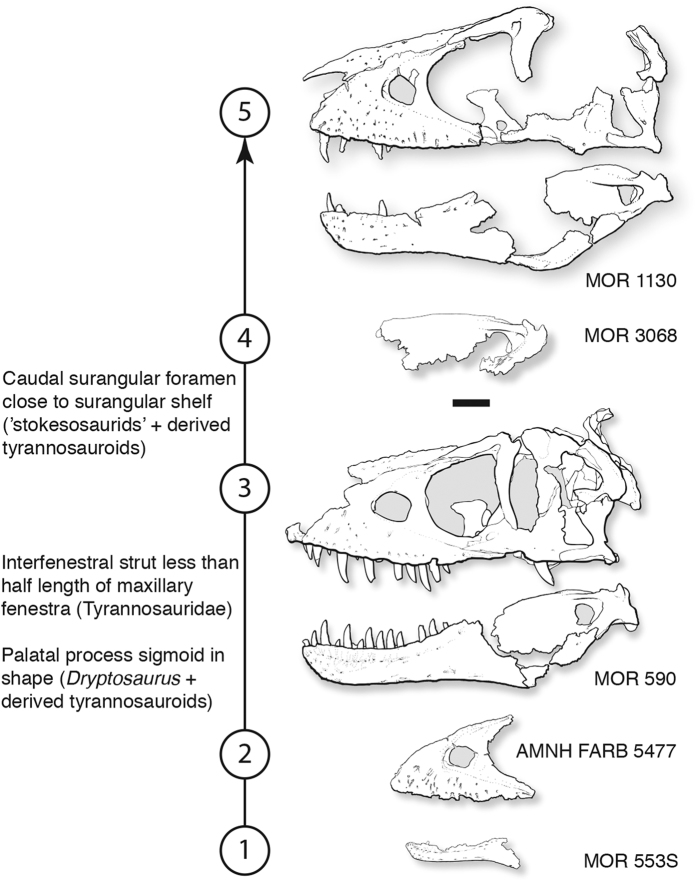
The growth series of *Daspletosaurus horneri* sp. nov., based on parsimony analysis. Unambiguously optimized derived phylogenetic characters were recovered as synontomorphies at two of the five growth stages, which are labeled at the corresponding numbers. Scale bar equals 10 cm. Abbreviations: AMNH FARB, American Museum of Natural History, Fossil Amphibians, Reptiles, and Birds; MOR, Museum of the Rockies.

The growth series of *D. horneri* includes juveniles, subadults, and an adult (Fig. 3). The smallest specimen, a dentary, has a tooth row that is 221.5 mm long, in contrast to the 423.0 mm tooth row of the adult, which has a 947.0 mm long skull. The length of the dentary tooth row has been shown by Currie^38^ that in comparison with skull length, it shows a weak negative allometry. Therefore, the original skull length of the small specimen was greater than 496.0 mm, slightly more than half the length of the adult skull.

The parsimony analysis of morphological features (see Supplementary ontogenetic character list, Data S1) resulted in a single most parsimonious growth series of 168 steps, with a CI of 0.93, an HI of 0.07, an RI of 0.93, and an RC of 0.91 (Fig. 3; see Supplementary Fig. S4). The transition from a gracile juvenile to a robust adult in *D. horneri* is similar to that seen in other tyrannosauroids^8^,^26^,^28^.

### Soft tissues

The excellent quality of preservation of these specimens permits us to assess the type of soft tissue that covered the face (premaxilla, maxilla, nasal, lacrimal, jugal, postorbital, squamosal, dentary). In *D. horneri*, and in all derived tyrannosauroids, the subcutaneous texture is coarse and shows a hierarchy of textures (see Supplementary Discussion S3). In order to identify the soft tissue that produced this complex surface, we compared the condition of tyrannosaurids with that of crocodylians (*Alligator mississippiensis*) and birds (*Struthio camelus, Anser* sp., *Anas* sp., *Cygnus* sp., *Meleagris gallopavo*), and we followed several studies^29^,^31^,^39^,^40^ to identify osteological correlates imprinted on the cortical surface of facial bones to deduce their causal soft tissues.

**Figure 4:**
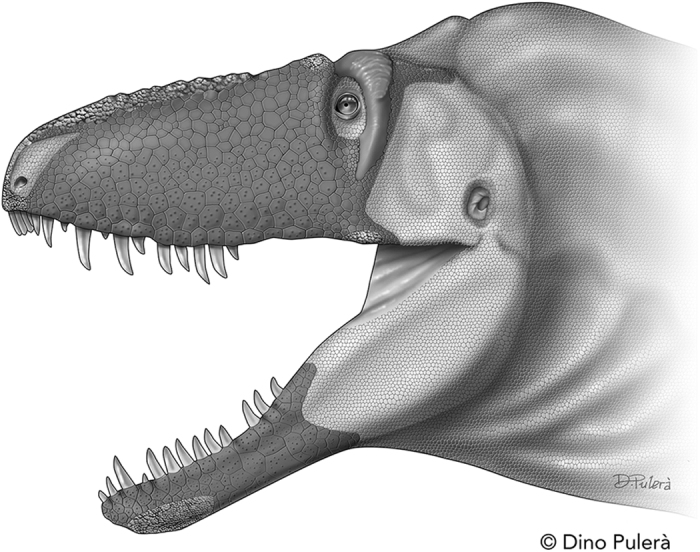
The craniofacial epidermis of *Daspletosaurus horneri* sp. nov., based on comparison with its closest living relatives, crocodylians and birds. Bone texture indicates large zones of large, flat scales and subordinate regions of armor-like skin and cornified epidermis; integumentary sense organs occur on the flat scales that cover the densest regions of neurovascular foramina. The region outside of the crocodylian-like skin is reconstructed with small scales after fossilized skin impressions of tyrannosaurids. This figure is not covered by the CC BY licence. Illustration © Dino Pulerà. All rights reserved, used with permission.

Although tyrannosaurids, crocodylians, and birds share neurovascular foramina that are densely clustered at the front of the jaws and form rows along the oral margin, the smooth texture of the snout in birds is sharply different from the hummocky texture of the facial bones of crocodylians and tyrannosaurids. The coarse surface of crocodylians is covered by many flat scales^29^, whereas in birds the rhamphotheca (beak) covered the smooth surface of the snout and jaws. The coarse texture shared between tyrannosaurids and crocodylians indicates a primary covering of flat scales on the face of the nonavian dinosaurs (Fig. 4).

In archosaurs, a scaly integument appears to be correlated with the multiple rows of foramina seen in crocodylians and tyrannosaurids, whereas in birds the foramina deep to the beak are limited to the jaw tips, the caudal end of the maxilla, and in a row along the side of the lower jaw. A similar localization of foramina is seen in ornithischian dinosaurs, whose jaws and oral margins were sheathed by beaks ahead of the tooth row^39^.

Unlike crocodylians, the alveolar row of foramina in the dentary of derived tyrannosauroids and birds occurs in a common groove that extends for much of the length of the bone. This groove is not seen in crocodylians, although caudodorsally extending sulci extend from the foramina. In birds the groove is covered by the rhamphotheca, and the ventral branches of the rictal vessels and the external branch of the mandibular nerve lie in the groove^40^. The groove in tyrannosauroids is shallowly inset, unlike the sharply inset condition that is seen in birds. The presence of the groove in tyrannosauroids indicates that the ventral branches of the rictal vessels evolved prior to, and might have been prerequisites for, the later appearance of the beak (in terms of the stepwise vascular changes leading from nonbeak to beak), at least on the lower jaw. The caudal end of the groove fades out in birds, a condition that is also seen in mature tyrannosauroids^41^.

Variation in the coarse zone of the face in tyrannosaurids indicates a variety of epidermal types, in addition to flat scales. The rostral surface of the premaxilla, dorsum of the nasals, dorsolateral surface of the lacrimal, cornual process of the jugal, and the rostroventrolatral surface of the dentary, bear small bony papillae, which indicate regions of armor-like skin^29^ (Fig. 4, see Supplementary Discussion S3). Finally, the coarse and rim-like edges of the postorbital horn, and its smooth central region, indicate a cornified sheath-like covering on its surface and part of the postorbital bar (Fig. 4).

## Discussion

### Anagenesis

A hypothesis of anagenesis amongst tyrannosaurids (*D. torosus* ->*D. horneri* ->*T. rex*) is defensible if: (1) the taxa are sister species or a phylogenetically successive series of species, (2) the species are stratigraphically sequential, (3) the phylogenetic relationships do not conflict with their stratigraphic sequence, and the taxa (4) are from the same landmass or adjacent landmasses that had connections that do not conflict with their chronological sequence.

Our new phylogeny (Fig. 2A) shows that *D. torosus* and *D. horneri* are sister species, whereas *T. rex* is nested in a separate clade. This topology is different from the phylogenetic arrangement of previous workers^1^, where *D. torosus* is the sister species of a clade composed of *D. horneri* and *T. rex*. In our phylogeny, *T. rex* is separated from the *Daspletosaurus* clade by two phylogenetically- and almost certainly stratigraphically-successive^4^ sister species, *Zhuchengtyrannus magnus* and *T. bataar*.

Chronostratigraphic and lithostratigraphic correlation between the TMF and Dinosaur Park Formation (DPF) suggests that the DPF correlates with the upper 220 m of the TMF^42^,^43^,^44^,^45^. In-progress high-precision U-Pb dating of the four ash beds spanning the top of the Oldman Formation, the DPF and the lower part of the Bearpaw Formation (BPF) indicates an age range between 76.69 Ma–74.26 Ma (internal error <±30 ka) for this stratal package^46^. Given that uppermost date of 74.26 Ma is from the lower BPF and these workers have identified a slowdown in sedimentation rates for the upper 22 meters of the DPF-BPF transition^46^, the age of the top of the DPF is likely no younger than 74.5 Ma. Because *D. torosus* specimens are restricted to the lower two-thirds of the DPF (~76.7–75.2 Ma), and *D. horneri* specimens are limited to the uppermost part of the TMF^45^ (~75.1–74.4 Ma) there appears to be little or no stratigraphic overlap between *Daspletosaurus* specimens from these two field areas. New high-precision CA-TIMS U-Pb dating is underway and promises to better resolve stratigraphic uncertainties.

Therefore, *D. torosus* and *D. horneri* meet the primary criteria for anagenesis: They are sister species, stratigraphically successive, and are from the Northern Rocky Mountain Region. In contrast, the divergence between the *Daspletosaurus* clade on the one hand, and the *Z. magnus* + *Tyrannosaurus* clade on the other, was the result of a cladogenetic (lineage-splitting) event, since they do not form a continuous series of stratigraphically sequential taxa. Therefore, *T. rex* was not a continuation of the anagenetic *Daspletosaurus* lineage^1^.

The *Z. magnus* + *Tyrannosaurus* clade consists of a pair of sister species (*T. bataar* + *T. rex*) and a sister taxon that extends from the preceding node (*Z. magnus*). This topology is consistent with a hypothesis of anagenesis: *Z. magnus* lived no less than 73.5 Ma^47^, and *T. rex* lived between 66.0 and ~67.2–67.4 Ma^48^. The age of the Nemegt Formation, from which *T. bataar* has been collected, is less than 75.0 Ma based on radiometric dating of the underlying Barun Goyot Formation^49^. Therefore, if *T. bataar* is intermediate in age between *Z. magnus* and *T. rex*, then the chronological sequence of these tyrannosaurs is also consistent with anagenesis. The time gap (~6.1–6.3 Myrs) that separates *Z. magnus* and *T. rex* greatly exceeds that between *D. torosus* and *D. horneri*; therefore, the hypothesis of anagenesis from *Z. magnus* to *T. rex* will be tested as new tyrannosaur specimens are collected from that interval in Asia and included in new phylogenetic analyses.

This is also the case for the two sister species of *Albertosaurus*, which are stratigraphically successive and from the same geographic region, in northern Laramidia^37^,^50^. If the Asian sister species *Alioramus remotus* and *A. sinensis* are shown to be stratigraphically successive (the taxa are widely separated geographically, and neither is constrained by radiometric dates^5^,^10^), then they meet the criteria as well, but in the absence of those data the decision between anagenesis and cladogenesis is equivocal.

Based on this approach, the evidence for anagenesis might be widespread among other dinosaur lineages with high-quality fossil records. If so, then anagenesis was an important contributor towards the generation of species diversity, in addition to cladogenesis, and in some instances cladogenesis might be an artifact of an incomplete fossil record.

### Ontogenetic tooth count reduction

*D. horneri* shows another occurrence of growth related reduction in maxillary tooth count among tyrannosaurids, which starts in juveniles (AMNH FARB 5477) with 15 maxillary teeth, increases to 17 tooth positions in subadults (MOR 590), and then decreases to 15 in adults (MOR 1130). In contrast, the number of dentary teeth is constant, where 17 alveoli are seen in juveniles (MOR 553S/7.19.0.97), subadults (MOR 590) and adults (MOR 1130).

The pattern of tooth count increase followed by a decrease in *D. horneri* may result from individual variation. However, this general pattern is also seen in the maxilla of *D. torosus*, where the least mature specimen (TMP 1994.143.0001) has a count of 13 teeth that increases in more mature specimens to 15 (AMNH FARB 5346) or 16 (MOR 395), which is followed by a decrease in the most mature specimens (CMN 8506) to a minimum of 14.

The phenomenon of growth related tooth count reduction is seen in the maxilla and dentary of *T. rex*^26^,^28^. The pattern of an initial increase to a later decrease is also seen in the recently published data for *T. rex* of Brown *et al*.^51^, where an increase in tooth count of the dentary (from 16 to 17) is seen in the two least mature specimens (CMNH 7541, BMRP 2002.4.1). Work in progress by one of us (TDC) finds that the smaller CMNH 7541 is the less mature specimen of the two. An increase is potentially also seen in the maxilla, where the tooth count for one specimen (BMRP 2002.4.1) is different between sides (left: 16, right: 15), but the authors only included the lower count in their data set^51^. Including the higher count would result in an increase from 15 to 16 teeth, followed by a decrease in number with increasing size, as seen in their data. If the tooth counts reflect ontogeny, and allowing for slightly different rates of tooth increase on each side of the jaw, it appears that an initial increase in tooth count followed by a decrease in number is typical of derived tyrannosaurines.

### Ontogenetic facial texture reduction

There is an ontogenetic reduction in the coarseness of the facial texture from subadult (MOR 590) to adult (MOR 1130), especially of the nasal bones that form the top of the snout, where the texture is reduced. Examination of specimens of *T. rex* reveals the same pattern, where the snout is coarse in relatively young adults (AMNH FARB 5027), whereas it is smoother in relatively old adults (LACM 23844)^28^. This reduction in texture that is seen among tyrannosaurines is similar to the reduction in cephalic ornamentation that also occurs in other dinosaurs, including ceratopsids^52^,^53^,^54^ and pachycephalosaurians^55^. It is possible that ornament reduction in adults is an ancestral feature for dinosaurs. A complete list of synontomorphies (growth-related features) and an extended discussion of the growth series can be found in the Supplementary synontomorphy list and Discussion S4.

### Quadratojugal foramen

It has been claimed that the presence of a large pneumatic foramen in the lateral surface of the quadratojugal is unique to the contentious tyrannosaurid taxon *Nanotyrannus lancensis*^56^, the holotype of which has been revised to represent a juvenile *T. rex*^26^,^28^. However, this feature is also seen in the adult specimen of *D. horneri*, and in an isolated tyrannosaurid quadratojugal from the upper Campanian strata of southern Alberta (CMN 57080). Although our phylogenetic analysis shows that the quadratojugal foramen evolved independently in *D. horneri* and *T. rex*, the additional occurrence in the unidentified taxon from Alberta indicates that the foramen might be synapomorphic for derived tyrannosaurines.

### Phylogenetic implications

The phylogenetic analysis places *D. horneri* as the sister species of *D. torosus*, indicating a monophyly of the known species within *Daspletosaurus*, in contrast to a recent hypothesis that the clade is paraphyletic^4^. We attribute the difference in results to the absence of the lacrimal, frontal, and vomer characters from the data set of Loewen *et al*.^4^. Therefore, the *Daspletosaurus* lineage traces back to the interval between the emplacement of the WIS at 99.5 Ma, as represented by the Thermopolis Shale^57^, and approximately 80 million years ago, the age of the oldest tyrannosaurines from the American West^4^.

### Biogeographic implications

The stratigraphic distribution of specimens discussed above shows that *Daspletosaurus* spp. inhabited northern Laramidia (what is now southern Alberta and northern Montana) for a minimum duration of ~2.3 Ma. This areal distribution makes *Daspletosaurus* one of the most widely distributed Late Cretaceous tyrannosaurid clades in Laramidia before the arrival of *T. rex*; in contrast, the distribution of other taxa is marked by high endemism, where species are limited to local basins of deposition^4^. In Alberta, *D. torosus* was sympatric with another large tyrannosaurid, *Albertosaurus libratus*^37^, but in Montana, at present, no sympatric tyrannosaurids are known.

Dispersal of tyrannosaurines from Laramidia to Asia must have occurred in that span (99.5 to ~80.0 Ma), to account for the presence in Asia of the relatively basal *Alioramus* and derived *Tyrannosaurus* (both from the Nemegt Formation, ~75.0–66.0 Ma^49^), and the derived *Z. magnus* (Wangshi Group, 73.5+ Ma^47^). Also, there is no evidence for faunal exchange between the landmasses from 80 to 75.7 Ma, so the dispersal had to occur prior to this point. Since *Daspletosaurus* is the sister species of the *Zhuchengtyrannus* + *Tyrannosaurus* clade, both had to arise before the dispersal event. From these observations we predict that species of the *Daspletosaurus* lineage will be present in rocks several Myrs older than the oldest existing examples of individuals from that lineage (from the DPF, ~76.7 Ma).

### Soft tissue interpretations

In tyrannosaurids, a scaly facial integument in association with multiple rows of neurovascular foramina on the snout and jaws serves as a reliable proxy for tactile sensitivity in these giant predatory dinosaurs. In crocodylians the craniomandibular foramina convey hundreds of afferent branches from the trigeminal nerve (N. V) in a density that transmits high resolution tactile sensations from the skin, making their snouts more sensitive than human fingertips^58^. The density of foramina in crocodylians maps with the distribution of the integumentary sensory organs (ISOs) in the skin of the head, where they are densest at the rostral end of the jaws and along the oral margins adjacent to the teeth, and sparsest on the sides of the jaws and top of the snout. Given the nearly identical arrangement and density of foramina to crocodylians, we can now infer that tyrannosaurids possessed ISOs (Fig. 4) to transmit trigeminal innervation from the facial skin, as in seen in crocodylians and comparable mechanoreceptors of other terrestrial tetrapods (e.g., monotremes, moles, toads, frogs, snakes, ducks)^58^.

If our soft–tissue inferences regarding the presence of flat facial scales and ISOs are correct, then behavioral inferences for tyrannosaurids can be drawn based on comparison with crocodylians^59^. Tyrannosaurids had a highly sensitive facial tactile system that functioned in prey capture, and object identification and manipulation, given the skeletal similarities with crocodylians^58^,^59^. In crocodylians the bony casements around the nerve branches protect them from injuries sustained during communal feeding while maintaining highly sensitive skin^58^; given the similarity in foramen morphology, this protective function was present in tyrannosaurids (whose bony oral margins often show lesions), showing that this multipurpose cephalic tactile system was not limited to life in an aquatic environment.

Assuming that crocodylians are suitable models for dinosaur behavior, and that tyrannosaurids were primarily terrestrial, tactile stimulation, such as rubbing, was probably more important in tyrannosaurid agonistic behavior than detecting water borne vibrations^59^. As in crocodylians, female tyrannosaurids would have relied upon ISOs on the snout for detecting the optimal temperature of a nest site, and for maintaining nest temperature and the nest materials; also, ISOs would have aided adult tyrannosaurids in harmlessly picking up eggs and nestlings and, in courtship, tyrannosaurids might have rubbed their sensitive faces together as a vital part of pre-copulatory play^59^.

## Methods

### Character dataset

We included *D. horneri* in an updated data matrix that is based on the most recent tyrannosauroid phylogeny^11^, which analyzed 366 characters, and we contributed another 20 new characters to the data matrix, for a total of 386 (see Supplementary Phylogenetic character list and Tables S5 and S6). We also included *Timurlengia euotica*^7^ and an undescribed species from the Iren Dabasu Formation that is currently under study by one of us (TDC), for a total of 28 ingroup taxa. Unlike some other analyses^4^, ours does not include *Bagaraatan ostromi* or *Alectrosaurus olseni*, but we include the referred material of *Teratophoneus curriei*. We take the view that *Alioramus altai* is synonymous with *A. remotus*, which we coded together into a single taxonomic unit. We regard *Raptorex kriegsteini* as a juvenile tyrannosaurine, which we excluded from the analysis.

### Parsimony analysis

We built the data matrix in MacClade 4 v.4.08^60^; we analyzed the data in a branch-and-bound search using PAUP* v. 4.0b10^61^. Ordering of characters followed reference^11^. As in the preceding analyses, *Allosaurus* sp., Maniraptora, Ornithomimosauria, and *Compsognathus longipes* were included as the outgroup taxa. The common components of the MPTs were summarized in a strict consensus and in a 50% majority rule consensus, which had the same topology (Fig. 2A); Bremer decay and bootstrap values we used to assess clade support (see Supplementary Fig. S3).

### Ontogeny

We reconstructed a hypothetical growth series for *D. horneri*, based on a cladistic analysis of 164 characters coded for 5 specimens (see Supplementary Data S1). We built the data matrix in MacClade 4 v.4.08^60^; we analyzed the data in a branch-and-bound search using PAUP* v. 4.0b10^61^ with the characters unordered and unweighted. An all-zero artificial embryo was included to optimize the characters on the topology.

### Data archiving

All primary data, including the geochronological analysis, phylogenetic character list and character-taxon matrix, ontogenetic character list and character-specimen matrix, are available in the supplementary information and Extended Data.

## Additional Information

**How to cite this article**: Carr, T. D. *et al*. A new tyrannosaur with evidence for anagenesis and crocodile-like facial sensory system. *Sci. Rep.* **7**, 44942; doi: 10.1038/srep44942 (2017).

**Publisher's note:** Springer Nature remains neutral with regard to jurisdictional claims in published maps and institutional affiliations.

## Supplementary Material

Supplementary Information

Supplementary Table S4

Supplementary Table S5

Supplementary Table S6

## References

[b1] Horner J. R., Varricchio D. J. & Goodwin M. B. Marine transgressions and the evolution of Cretaceous dinosaurs. Nature 358, 59–61 (1992).

[b2] Carr T. D., Williamson T. E., Britt B. B. & Stadtman K. Evidence for high taxonomic diversity and morphologic tyrannosauroid diversity in the Late Cretaceous (Late Campanian) of the American Southwest and a new short-skulled tyrannosaurid from the Kaiparowits Formation of Utah. Naturwissen. 98(3), 241, doi: 10.1007/s00114-011-0762-7 (2011). 21253683

[b3] Brusatte S. L. . Tyrannosaur paleobiology: new research on ancient exemplar organisms. Science 329, 1481–1485 (2010). 20847260 10.1126/science.1193304

[b4] Loewen M. A., Irmis R. B., Sertich J. J. W., Currie P. J. & Sampson S. D. Tyrant dinosaur evolution tracks the rise and fall of Late Cretaceous oceans. PLoS One 8(11), e79420, doi: 10.1371/journal.pone.0079420 (2013). 24223179 PMC3819173

[b5] Brusatte S. L., Carr T. D., Erickson G. M., Bever G. S. & Norell M. A. A long-snouted, multihorned tyrannosaurid from the Late Cretaceous of Mongolia. Proc. Nat. Acad. Sci. USA 106, 17261–17266 (2009). 19805035 10.1073/pnas.0906911106 PMC2765207

[b6] Fiorillo A. R. & Tykowski R. S. A diminutive new tyrannosaur from the top of the world. PLoS One 9(3), e91287, doi: 10.1371/journal.pone.0091287 (2014). 24621577 PMC3951350

[b7] Brusatte S. L., Averianov A., Sues H.-D. & Butler I. B. New tyrannosaur from the mid-Cretaceous of Uzbekistan clarifies the evolution of giant body sizes and advanced senses in tyrant dinosaurs. Proc. Nat. Acad. Sci. USA 113(13), 3447, doi: 10.1073/0nas.1600140113 (2016). 26976562 PMC4822578

[b8] Carr T. D. & Williamson T. E. *Bistahieversor sealeyi*, gen. et sp. nov., a new tyrannosauroid from New Mexico and the origin of deep snouts in Tyrannosauroidea. J. Vert. Paleontol. 30, 1–16 (2010).

[b9] Carr T. D., Williamson T. E. & Schwimmer D. R. A new genus and species of tyrannosauroid from the Late Cretaceous (Middle Campanian) Demopolis Formation of Alabama. J. Vert. Paleontol. 25, 119–143 (2005).

[b10] Lü . A new clade of Asian Late Cretaceous long-snouted tyrannosaurids. Nature comm., doi: 10.1038/ncomms4788 (2014). 24807588

[b11] Brusatte S. L. & Carr T. D. The phylogeny and evolutionary history of tyrannosauroid dinosaurs. Sci. Reports 6, 20252, doi: 10.1038/srep20252 (2016). PMC4735739 26830019

[b12] Wagner P. J. & Erwin D. H. In New approaches to speciation in the fossil record (eds Erwin D. H., Anstey R. L.) 87–121 (Columbia Univ. Press, 1995).

[b13] Bell M. A. Implications of a fossil stickleback assemblage for Darwinian gradualism. Jour. Fish Bio. 75, 1977–1999 (2009). 20738668 10.1111/j.1095-8649.2009.02416.x

[b14] Gingerich P. D. Species in the fossil record: concepts, trends, and transition. Paleobiol. 11, 27–41 (1985).

[b15] Wood R. M. & Mayden R. L. Speciation and anagenesis in the genus *Cyprinella* of Mexico (Teleostei: Cyprinidiae): a case study of Model III allopatric speciation. Rev. In Fish Bio. & Fisheries 12, 253–271 (2002).

[b16] López-Sepúlveda P. . Progressive migration and anagenesis in Drimys confertifolia of the Juan Fernández Archipelago, Chile. Jour. Plant Res. 128, 73–90 (2015). 25292282 10.1007/s10265-014-0666-7 PMC4300435

[b17] Stuessy T. F. . Anagenetic evolution in island plants. Jour. Biogeogr. 33, 1259–1265 (2006).

[b18] Freedman-Fowler E. A. & Horner J. R. A new brachylophosaurin hadrosaur (Dinosauria: Ornithischia) with an intermediate nasal crest from the Campanian Judith River Formation of northcentral Montana. PLoS One 10, doi: 10.1371/journal.pone.0141304 (2015). PMC4641681 26560175

[b19] Scannella J. B., Fowler D. W., Goodwin M. B. & Horner J. R. Evolutionary trends in *Triceratops* from the Hell Creek Formation, Montana. PNAS 111, 10245–10250 (2014). 24982159 10.1073/pnas.1313334111 PMC4104892

[b20] Gould S. J. The Structure of Evolutionary Theory 1–1433 (Harvard Univ. Press, 2002).

[b21] Liow L. H. In Encyclopedia of Life Sciences (eds), doi: 10.1002/9780470015902.a0001666.pub2 (J. Wiley & Sons, 2010).

[b22] Podani J. R. Tree thinking, time and topology: comments on the interpretation of tree diagrams in evolutionary/phylogenetic systematics. Cladistics 29, 315–327 (2013). 10.1111/j.1096-0031.2012.00423.x 34818822

[b23] Vaux F., Trewick S. A. & Morgan-Richards M. Lineages, splits and divergence challenge whether the terms anagenesis and cladogenesis are necessary. Biol. Jour. Linn. Soc. 117, doi: 10.111/bij.12665 (2015).

[b24] Campione N. E. & Evans D. C. Cranial growth and variation in edmontosaurs (Dinosauria: Hadrosauridae): implications for Latest Cretaceous megaherbivore diversity in North America. PLoS One 6, e25186 (2011). 21969872 10.1371/journal.pone.0025186 PMC3182183

[b25] Strotz L. C. & Allen A. P. Assessing the role of cladogenesis in macroevolution by integrating fossil and molecular evidence. PNAS 110, 2904–2909 (2013). 23378632 10.1073/pnas.1208302110 PMC3581934

[b26] Carr T. D. Craniofacial ontogeny in Tyrannosauridae (Dinosauria: Coelurosauria). J. Vert. Paleontol. 19, 497–520 (1999).

[b27] Carr T. D. A taxonomic assessment of the type series of *Albertosaurus sarcophagus* and the identity of Tyrannosauridae (Dinosauria, Coelurosauria) in the *Albertosaurus* bonebed from the Horseshoe Canyon Formation (Campanian-Maastrichtian, Late Cretaceous). Can. J. Earth Sci. 47, 1213–1226 (2010).

[b28] Carr T. D. & Williamson T. E. Diversity of late Maastrichtian Tyrannosauridae (Dinosauria: Theropoda) from western North America. Zool. J. Linn. Soc. 142, 479–523 (2004).

[b29] Hieronymus T. L., Witmer L. M., Tanke D. H. & Currie P. J. The facial integument of centrosaurine ceratopsids: morphological and histological correlates of novel skin structures. Anat. Record 292, 1370–1396 (2009). 10.1002/ar.20985 19711467

[b30] Witmer L. M. The evolution of the antorbital cavity of archosaurs: a study in soft-tissue reconstruction in the fossil record with an analysis of the function of pneumaticity. J. Vert. Paleontol. 17, 1–73 (1997).

[b31] Hieronymus T. L. & Witmer L. M. Homology and evolution of avian compound rhamphothecae. Auk 127(3), 590–604 (2010).

[b32] Rogers R. R., Swisher C. C. III & Horner J. R. 40Ar/39Ar age correlation of the nonmarine Two Medicine Formation (Upper Cretaceous), northwestern Montana, USA. Can. Jour. of Earth Sci. 30, 1066–1075 (1993).

[b33] Lorenz J. C. & Gavin W. Geology of the Two Medicine Formation and the sedimentology of a dinosaur nesting ground. Montana Geological Society Field Guide 175–186 (1984).

[b34] Roberts E. M., Sampson S. D., Deino A. D., Buchwaldt R. & Bowring S. A. In Advances in Late Cretaceous Western Interior Paleontology and Geology (eds Titus A., Loewen M. A.) 85–106 (Indiana Univ. Press, 2013).

[b35] Scherzer B. A. & Varricchio D. J. Taphonomy of a juvenile lambeosaurine bonebed from the Two Medicine formation (Campanian) of Montana, United States. Palaios 25, 780–795 (2010).

[b36] Holtz T. R. In Mesozoic Vertebrate Life (eds Carpenter K. & Tanke D.) 64–83 (Indiana Univ. Press, 2001).

[b37] Currie P. J. Cranial anatomy of tyrannosaurid dinosaurs from the late Cretaceous Alberta, Canada. Acta Palaeontol. Pol. 48, 191–226 (2003).

[b38] Currie P. J. Allometric growth in tyrannosaurids (Dinosauria: Theropoda) from the Upper Cretaceous of North America. Can. J. Earth Sci. 40, 651–665 (2004).

[b39] Papp M. J. A Critical Appraisal of Buccal Soft-Tissue Anatomy in Ornithischian Dinosaurs. Unpublished MSc Dissertation, Ohio University, Athens, 229 pp (2000).

[b40] Sedlmayr J. C. Anatomy, Evolution, and Functional Significance of Cephalic Vasculature in Archosauria. Unpublished PhD Dissertation, Ohio University, Athens, 398 pp (2002).

[b41] Brusatte . Dentary groove morphology does not distinguish ‘*Nanotyrannus’* as a valid taxon of tyrannosaurid dinosaur. Comment on: “Distribution of the dentary groove of theropod dinosaurs: implications for theropod phylogeny and the validity of the genus *Nanotyrannus* Bakker *et al*. 1988”. Cret. Res., doi: 10.1016/j.cretres.2016.02.007 (2016).

[b42] Rogers R. R. Sequence analysis of the Upper Cretaceous Two Medicine and Judith River Formations, Montana: nonmarine response to the Claggett and Bearpaw marine cycles. J. Sed. Res. 68, 615–631 (1998).

[b43] Roberts E. M., Deino A. L. & Chan M. A. ^40^Ar/^39^Ar age of the Kaiparowits Formation, southern Utah, and correlation of contemporaneous Campanian strata and vertebrate faunas along the margin of the Western Interior Basin. Cret. Res. 26, 307–318 (2005).

[b44] Eberth D. A. In Dinosaur Provincial Park: A Spectacular Ancient Ecosystem Revealed (eds Currie P. J. & Koppelhus E. B.) 54–82 (Indiana Univ. Press, 2005).

[b45] Currie P. J. & Russell D. A. In Dinosaur Provincial Park: A Spectacular Ancient Ecosystem Revealed (eds Currie P. J., Koppelhus E. B.) 202–220 (Indiana Univ. Press, 2005).

[b46] Eberth D. A., Ramezani J. R., Roberts E. M. & Bowring S. A. New TIMS U-Pb geochronology from the Belly River Group (Upper Cretaceous) at Dinosaur Provincial Park, Alberta, Canada, and implications for dinosaur biostratigraphy of the Western Interior Basin. Geol. Soc. Am. Abs. Programs 48(7), doi: 10.1130/abs/2016AM-281038 (2016).

[b47] Hone D. W. E. . A new, large tyrannosaurine theropod from the Upper Cretaceous of China. Cret. Res. 32, 495–503 (2011).

[b48] Hicks J. F., Johnson K. R., Obradovich J. D., Tauxe L. & Clark D. In The Hell Creek Formation and the Cretaceous-Tertiary Boundary in the Northern Great Plains: An Integrated Continental Record of the End of the Cretaceous (eds Hartman J. H., Johnson K. R., Nichols D. J.) 35–55 (USGS Spec. Pap. 261, 2002).

[b49] Shuvalov V. F. In The Age of Dinosaurs In Russia and Mongolia (eds Benton M. J., Shoshkin M. A., Unwin D. M., Kurochkin E. N.) 256–278 (Cambridge Univ. Press, 2000).

[b50] Bell P. R. & Currie P. J. *Albertosaurus* (Dinosauria: Theropoda) material from an *Edmontosaurus* bonebed (Horseshoe Canyon Formation) near Edmonton: clarification of palaeogeographic distribution. Can. Jour. Earth Sci. 51(11), 1052, doi: 10.1139/cjes-2014-0050 (2014).

[b51] Brown C. M. . Tooth counts through growth in diapsid reptiles: implications for interpreting individual and size-related variation in the fossil record. J. of Anat., doi: 10.1111/joa.12280 (2015). PMC4386932 25689039

[b52] Horner J. R. & Goodwin M. B. Major cranial changes during *Triceratops* ontogeny. Proc. Royal Soc. B: Biol. Sci. 273, 2757, doi: 10.1098/rspb.2006.3643 (2006). PMC1635501 17015322

[b53] Frederickson J. A. & Tumarkin-Deratzian A. R. Craniofacial ontogeny in *Centrosaurus apertus*. PeerJ 2, e252, doi: 10.7717/peerj.252 (2014). 24688836 PMC3933270

[b54] Scannella J. B. & Horner J. R. ‘*Nedoceratops’*: an example of a transitional morphology. PLoS One 6(12), e28705, doi: 10.1371/journal.pone.0028705 (2011). 22194891 PMC3241274

[b55] Horner J. R. & Goodwin M. B. Extreme cranial ontogeny in the Upper Cretaceous dinosaur *Pachycephalosaurus*. PLoS One 4(1), ?, doi: 10.1371/journal.pone.0007626 (2009). PMC2762616 19859556

[b56] Larson P. In Tyrannosaurid Paleobiology (eds Parrish M. J., Molnar R. E., Currie P. J. & Koppelhus E. B.) 15–53 (Indiana Univ. Press, 2013).

[b57] Kirschbaum M. A. & Roberts L. N. R. In Petroleum Systems and Geologic Assessment of Oil and Gas in the Southwestern Wyoming Province, Wyoming, Colorado, and Utah 1–31 (U.S. Geol. Surv. Dig. Data Ser. DDS-69-D, 2005).

[b58] Leitch D. B. & Catania K. C. Structure, innervation and response properties of integumentary sensory organs in crocodilians. J. Exp. Bio. 215(23), 4217, doi: 10.1242/jeb.076836 (2012). 23136155 PMC4074209

[b59] Brazaitis P. & M. E. Watanabe Crocodilian behavior: a window to dinosaur behavior? Historical Biology 23, 73–90 (2011).

[b60] Maddison D. R. & Maddison W. P. MacClade 4: Analysis of phylogeny and character evolution. Version 4.08a. http://macclade.org (2005). 10.1159/000156416 2606395

[b61] Swofford D. L. PAUP*. Phylogenetic Analysis Using Parsimony (*and Other Methods). Version 4. Sinauer Associates, Sunderland, USA. http://paup.sc.fsu.edu (2002).

