# Supplementary material for: A new tyrannosaur with evidence for anagenesis and crocodile-like facial sensory system
Source: Sci Rep. 2017 Mar 30;7:44942. doi: 10.1038/srep44942 (PMC5372470; doi:10.1038/srep44942)
Supplement: Supplementary Information [file srep44942-s1.pdf]

Supplementary Information for:

A new tyrannosaur with evidence for anagenesis and crocodile-like facial sensory system

Thomas D. Carr[1]*, David J. Varricchio[2], Jayc C. Sedlmayr[3], Eric M. Roberts[4], Jason R. Moore[5].
[1]Carthage College, 2001 Alford Park Drive, Kenosha, WI 53140
[2]Department of Earth Sciences, Montana State University, P.O. Box 173480, Bozeman, MT 59717-3480
[3]School of Medicine, Louisiana State University, 1901 Perdido Street, New Orleans, LA 70112
[4]Geosciences, College of Science and Engineering, James Cook University, Townsville, QLD 4811, Australia
[5]Honors College, University of New Mexico, Albuquerque, NM 87131
*Correspondence to: tcarr@carthage.edu

1. Figure S1. U-Pb LA-ICPMS dating of zircon from a volcanic ash bed (bentonite) from the upper Two Medicine Formation.
2. Figure S2. Diagnostic characters of *Daspletosaurus horneri* sp. nov.
3. Figure S3. Strict consensus and 50% majority rule consensus tree of 18 equally parsimonious trees.
4. Figure S4. Ontogram for *Daspletosaurus horneri* sp. nov.
5. Discussion S1. U-Pb Results for the MOR 1130 Locality.
6. Discussion S2. Diagnostic Characters of *Daspletosaurus horneri.*
7. Discussion S3. Superficial Craniomandibular Soft Tissues in Tyrannosaurids.
8. Discussion S4. Growth Series.
9. Phylogenetic Character List.
10. Unambiguously Optimized Synapomorphies List.
11. Ontogenetic Character List.
12. Unambiguously Optimized Synontomorphies List.
13. References.
14. Data S1. Ontogenetic Specimen-Character Matrix.
15. Table S1. Craniomandibular Measurements of *Daspletosaurus horneri* sp. nov.
16. Table S2. Vertebral Measurements of *Daspletosaurus horneri* sp. nov.
17. Table S3. Appendicular Measurements of *Daspletosaurus horneri* sp. nov.
18. Table S4: DVTM-1 Isotope ratio tables. Includes data table, colour Terra-Wasserberg Concordia diagram, colour Weighted Average Age diagram, colour Sorted Weighted Avg. diagram, and DVTM-1 U-Pb data.
19. Table S5: Phylogenetic data matrix table; characters 1-255.
20. Table S6: Phylogenetic data matrix table; characters 256-386.

1. Figure S1. Results of U-Pb LA-ICPMS dating of zircon from a volcanic ash bed (DVTM-1) from the upper Two Medicine Formation. The (ordered) weighted mean age for sample DVTM-1 is calculated and shown in (A) and the lower intercept solution for the sample is shown on the Terra-Wasserberg plot in (B).

A

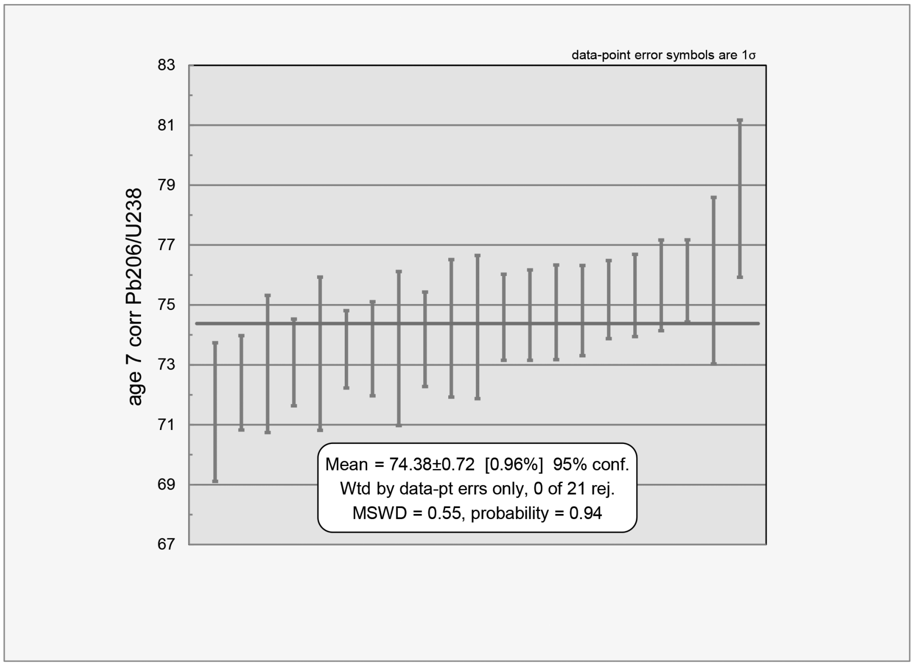

B

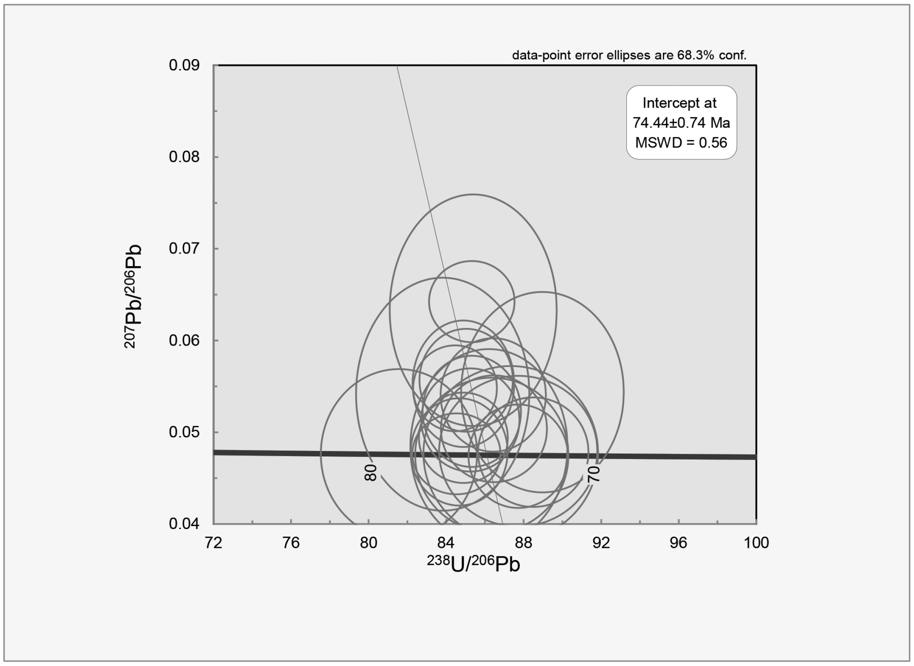

2. Figure S2. Comparison of selected diagnostic characters of *Daspletosaurus horneri* sp. nov. with *D. torosus*. Scale bars in centimeters, where shown. Orientation of some images are reversed for ease of comparison.

A. The rostral end of the snout in *D. horneri* is mediolaterally wider than in its closest relatives, which has extended the narrow condition of the teeth and interdental plates laterally. The first two plates in *D. horneri* are narrow, in contrast to the wide condition of *D. torosus*. The 'truncated' condition refers to the vertical and straight mesial edge of a plate, in contrast to the triangular margin of a wide plate.

B. In dorsal view, the dentary of a juvenile *D. horneri* (MOR 553S/7.19.0.97) curves laterally, which reflects the wide rostral end of the upper jaw, whereas the ramus is straight in juvenile *D. torosus* (TMP 1994.143.0001).

C. The rostral limit of the promaxillary sinus (rostral line) and the choana (caudal line); in *D. horneri* (MOR 590) the sinus does not extend as far rostrally as is seen in *D. torosus* (CMN 8506), and the rostral edge of the choana is positioned further caudally than in *D. torosus*.

D. The inflation of the dorsal surface of the lacrimal, where it does not reach the medial edge of the bone in *D. horneri* (MOR 590), whereas it extends medially *D. torosus* (CMN 8506).

E. In medial view, the medial pneumatic recess of the lacrimal in *D. horneri* (MOR 1130) is a small slot, whereas in *D. torosus* (CMN 8506) it is a large, circular opening.

F. The upper half of the orbital margin of the lacrimal in *D. horneri* (MOR 1130) is concave, whereas it is convex in *D. torosus* (CMN 8506).

G. In ventral view, the pneumatic recess in the ceiling of the squamosal in *D. horneri* (MOR 1130) is completely undercut around its margins, whereas in *D. torosus* (CMN 8506) its rostromedial corner is not undercut.

H. The rostral margin of the dorsotemporal fossa in *D. horneri* (MOR 590) is sinuous, whereas it is nearly straight in *D. torosus* (CMN 8506).

I. The joint surface for the squamosal covers the base of the caudolateral process of the parietal in *D. horneri* (MOR 590), whereas only its ventral half is covered in *D. torosus* (CMN 8506).

J. In lateral view, the tympanic ridge extends from the laterosphenoid onto the prootic in *D. horneri* (MOR 590), whereas it fades before reaching the prootic in *D. torosus* (CMN 8506).

K. In lateral view, the shaft of the quadratojugal is penetrated by a large foramen in *D. horneri* (MOR 1130), whereas in *D. torosus* (CMN 8506) a foramen is absent. Photograph of MOR 1130 courtesy of Scott A. Williams.

L. The notch between the basal tuber is shallow (less than 40% the depth of the basal tubera in *D. horneri* (MOR 1130), whereas it is tall in *D. torosus* (CMN 8506).

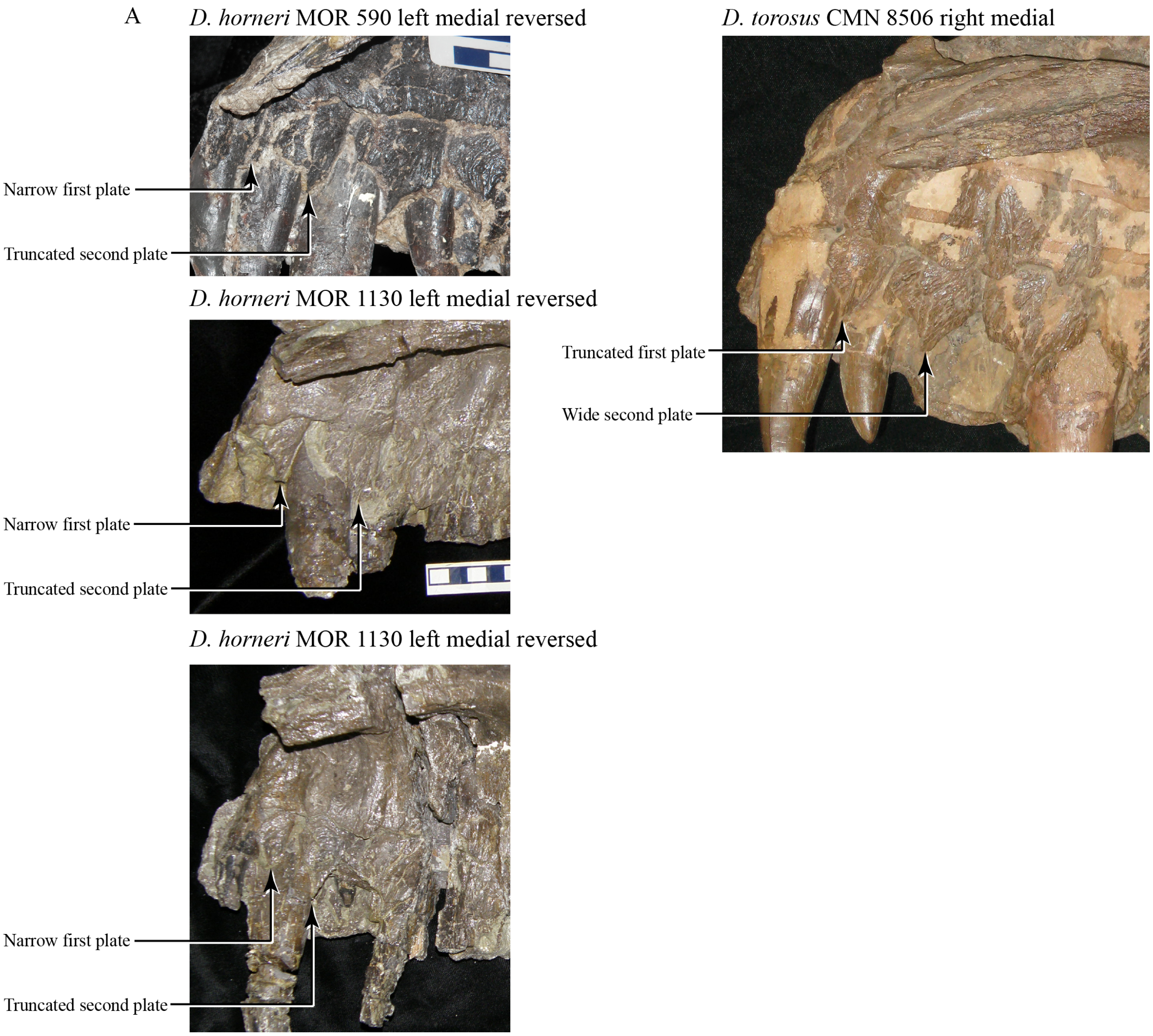
A
D. horneri MOR 590 left medial reversed
Narrow first plate
Truncated second plate
D. horneri MOR 1130 left medial reversed
Narrow first plate
Truncated second plate
D. horneri MOR 1130 left medial reversed
Narrow first plate
Truncated second plate
D. torosus CMN 8506 right medial
Truncated first plate
Wide second plate

B *D. horneri* MOR 553S/7.19.0.97 left dorsal

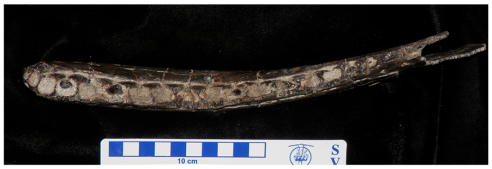

D. torosus TMP 1994.143.0001 dorsal right reversed

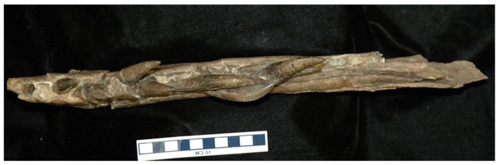

C *D. horneri* MOR 590 left reversed

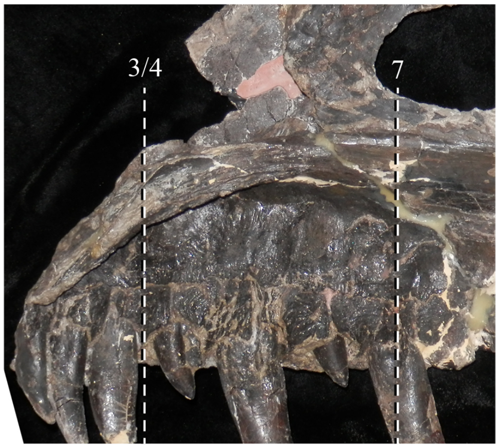

D. torosus CMN 8506 left reversed

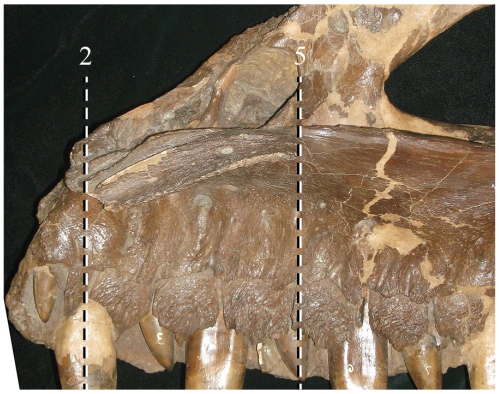

D *D. horneri* MOR 590 left

Inflation does not reach medial edge of bone

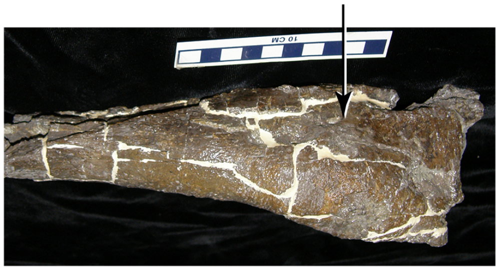

D. torosus CMN 8506 left

Inflation reaches medial edge of bone

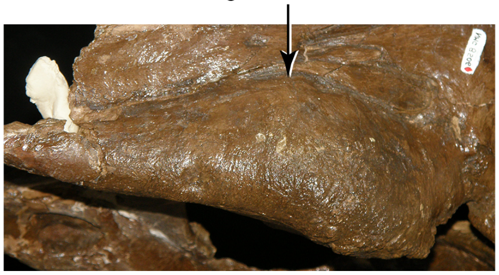

E *D. horneri* MOR 1130 left reversed

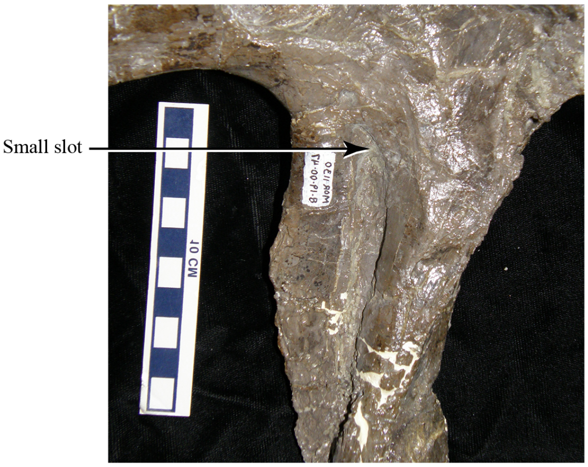

D. torosus CMN 8506 right

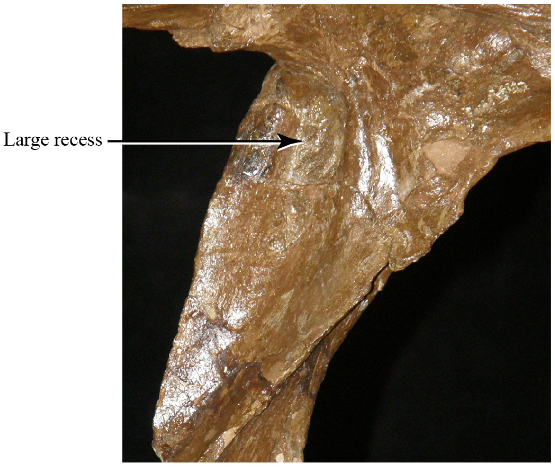

F *D. horneri* MOR 1130 left

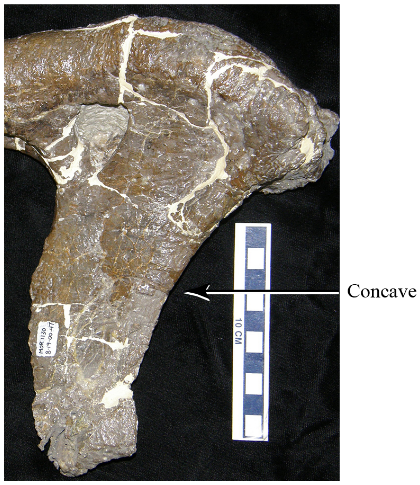

D. torosus CMN 8506 left

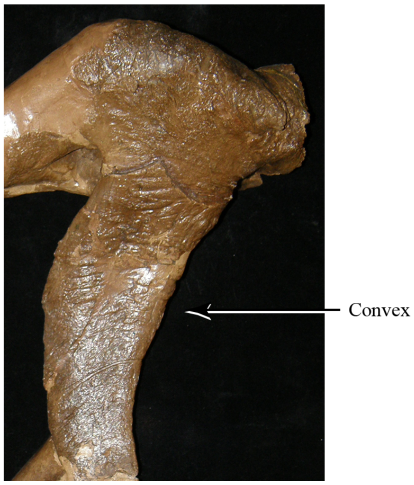

G *D. horneri* MOR 1130 right reversed

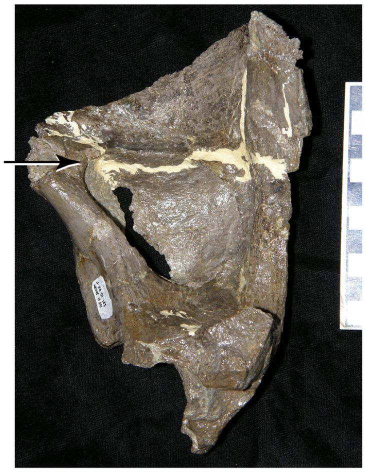

D. torosus CMN 8506 left

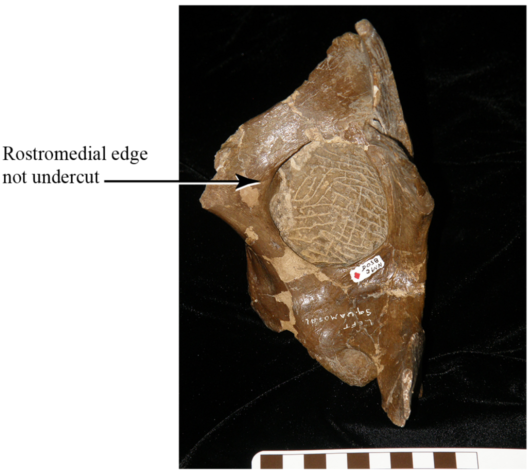

H *D. horneri* MOR 590

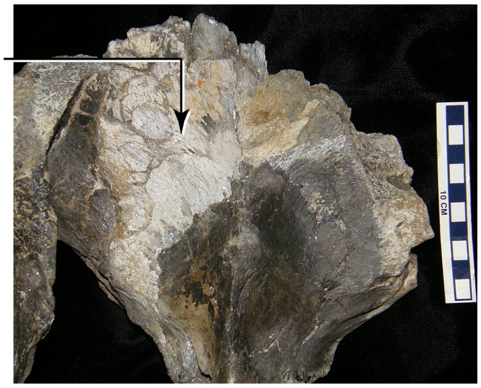

D. torosus CMN 8506

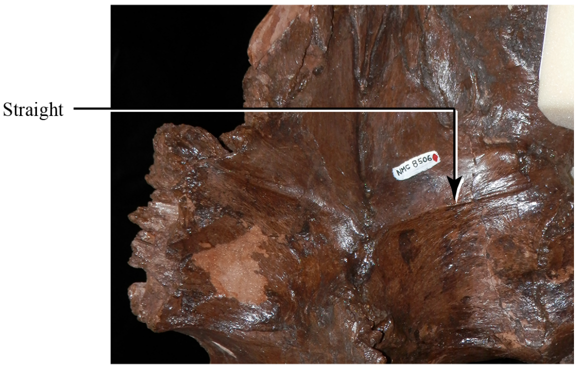

I *D. horneri* MOR 590 right reversed

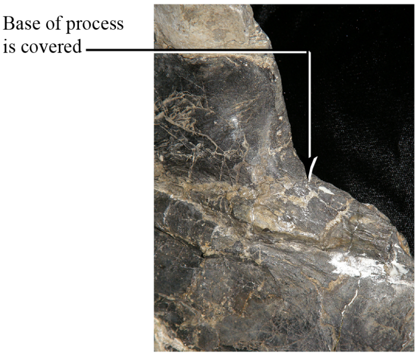

D. torosus CMN 8506 left

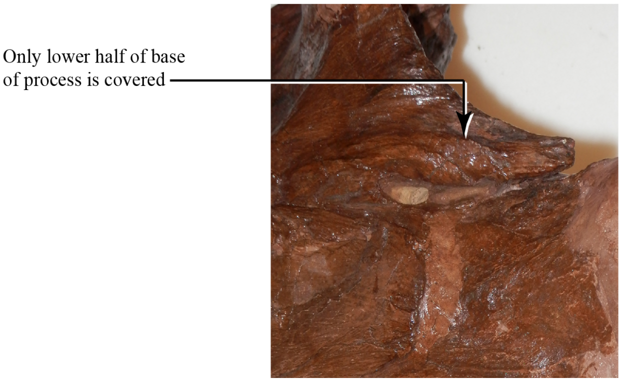

J *D. horneri* MOR 590 right reversed

tympanic ridge extends onto prootic

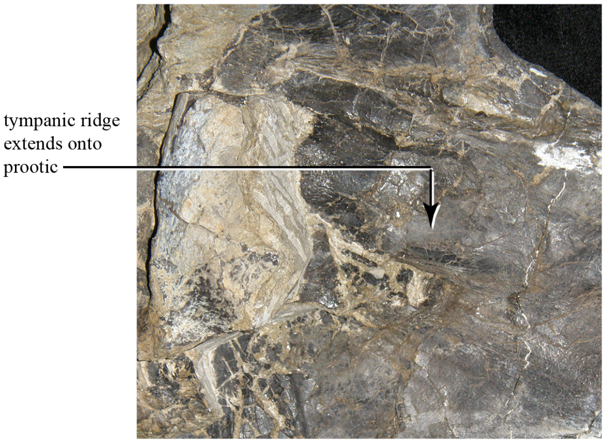

D. torosus CMN 8506 left

tympanic ridge does not extend onto prootic

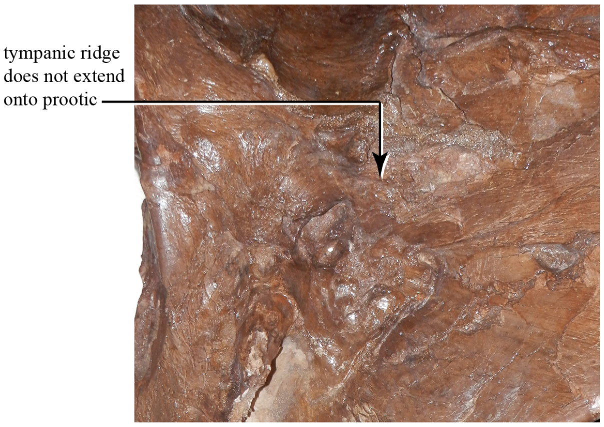

K *D. horneri* MOR 1130 right reversed

Foramen

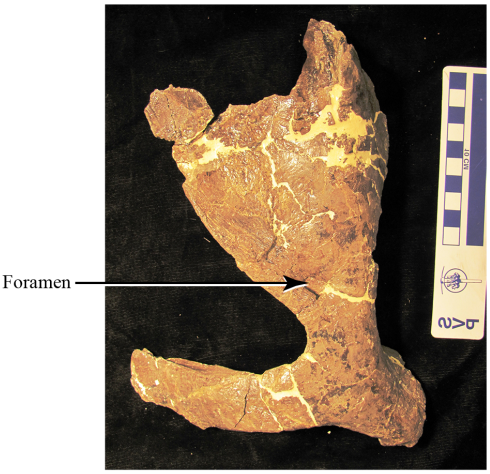

D. torosus CMN 8506 left

Imperforate

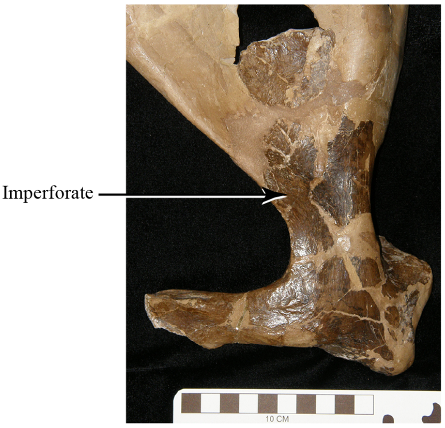

L *D. horneri* MOR 1130

Short notch

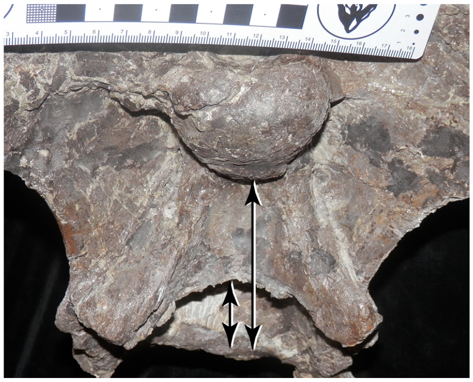

D. torosus CMN 8506 caudal

Tall notch

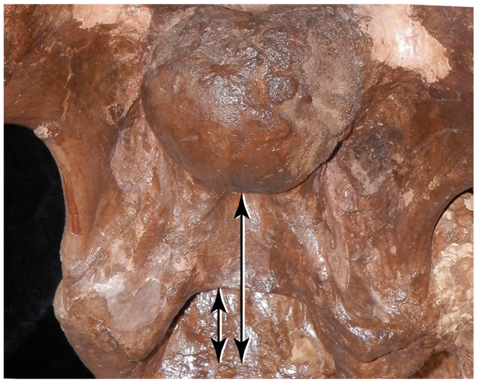

3. Figure S3. Topology of the strict consensus tree that summarizes 18 equally parsimonious trees; a 50% majority rule consensus tree results in the same topology. Numbers three and less indicate Bremer support values, numbers greater than three are bootstrap values (1000 replicates in TNT, Goloboff et al., 2008); nodes without bootstrap numbers have less than 50% support.

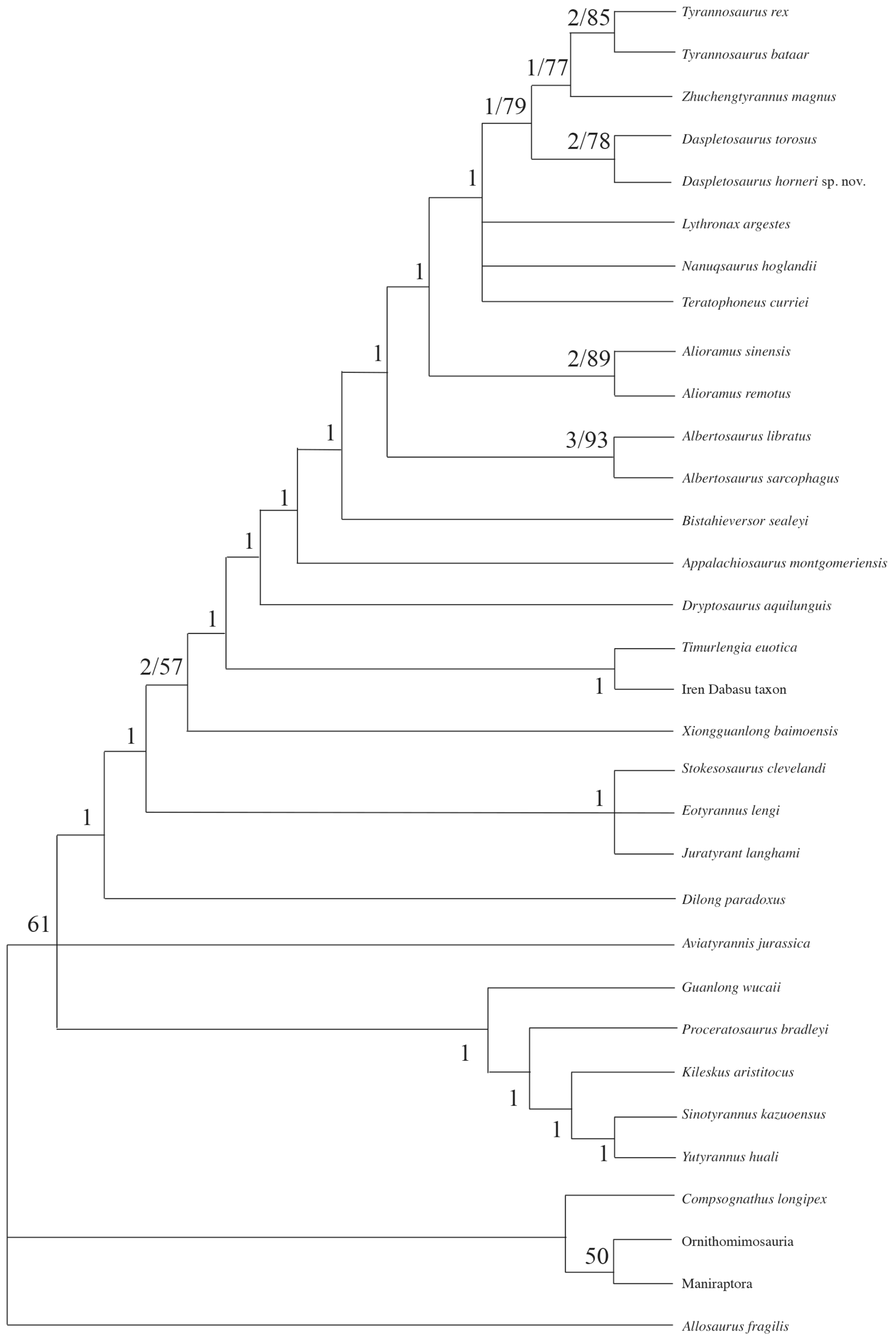

4. Figure S4. Ontogram for *Daspletosaurus horneri* sp.nov.

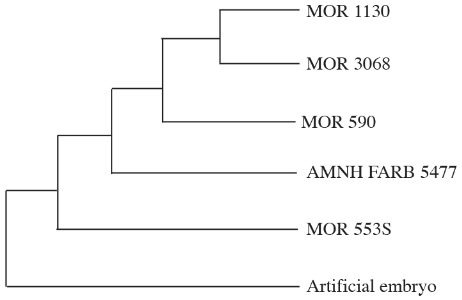

5. Discussion S1. U-Pb geochronology for the MOR 1130 locality
The MOR 1130 *Daspletosaurus* locality is located in a relatively well-exposed stratigraphic section along the Sun River in Teton County, Montana. Although a relatively continuous stratigraphic section is exposed along the Sun River and the nearby Pishkun Canal, this area is located in part of the Montana foreland basin known as the disturbed belt, and complex folding and faulting makes stratigraphic assessments challenging in this area. However, based on geologic mapping and measuring of sections in this area (e.g., Scherzer and Varricchio, 2010), MOR 1130 is placed near the top of the Two Medicine Formation. In order to confirm this assertion, a previously undated volcanic ashbed (DVTM-1) located 5.9 m below MOR 1130 was dated herein.

Sample Preparation

The sample was carefully collected to avoid contamination, and repeatedly washed and decanted in the lab to remove the clay-sized fraction. For this particular sample, the purity of the sample and lack of reworking of this bentonite was demonstrated by the lack of a detrital sand and silt component as part of the coarse-grained fraction. The coarse-grained (sand and coarse-silt sized) mineral separate was washed a final time in an ultrasonic bath, followed by density separation using lithium adjusted to a specific gravity of 2.85-2.87. The heavy mineral fraction was then washed, dried, and run through a Frantz magnetic separator at progressively higher magnetic currents of 0.5, 0.8, 1.3, and 1.5, set at a constant 10° side slope. The non-magnetic (>1.5) heavy mineral separates were then handpicking for zircon and only the clearest, most euhedral acicular zircons were selected for U-Pb dating. Roughly 60 zircons were mounted in a 25 mm epoxy resin puck, polished to expose their mid-sections and imaged using a Jeol JSM5410LV scanning electron microscope with attached cathodoluminescence detector in order to document microstructures, cracks, inclusions and other complexities.

U-Pb dating of zircon via laser ablation inductively coupled mass-spectrometry

All work was done at the Advanced Analytical Centre of James Cook University, using a Coherent GeolasPro 193nm ArF Excimer laser ablation system connected to a Bruker 820-MS (formerly Varian 820-MS). The ablation cell was connected to the Bruker 820-MS via Tygon tubing and a 3-way mixing bulb (volume ~5 cm^3). The standard cylindrical sample cell was used througout the study, but with a custom-designed polycarbonate insert to reduce the effective volume to 4 cm^3 (see Tucker et al., 2013). This insert combined with the mixing bulb provides both a very stable time-resolved signal and rapid signal washout.
The Bruker 820-MS employs an ion mirror design, which reflects the ion beam exiting the skimmer cone by 90° and focusses this into the mass analyzer. Non-ionized large particles and neutrals, as well as partially ionized particles, are not reflected and extracted by a pump located behind the mirror. In this way, the electrostatic mirror acts as a particle size filter to admit only fully atomized and ionized particles into the quardupole mass filter and detector. The advantage of this unique configuration is that it facilitates tuning of the ICP-MS to minimise instrumental mass fractionation focussing on the key ratio of Pb/U, as described below. The instrumental parameters and operating conditions are provided in Tucker et al. (2013).
All instrument tuning was performed using a 5 Hz repetition rate, 44 μm beam aperture and 6 J/cm^2 energy density, as determined by energy meter at the ablation site. Under these conditions, the ablation rate for NIST 610 and zircon was about 0.1 μm per laser pulse and 0.06 μm per laser pulse respectively. Tuning was achieved by iteratively adjusting the He carrier gas, Ar sampling

gas, sheath gas flow rate, RF Power, 1st, 2nd, 3rd Extraction lens and corner lens voltage to achieve $^{238}U/^{232}Th$ ratio ~1, ThO/Th < 1% typically 0.5% and $^{206}Pb/^{238}U$ ~ 0.22 in NIST610. Tuning the instrument towards the 'true' $^{206}Pb/^{238}U$ ratio thus minimizes the magnitude of the total Pb/U fractionation correction applied zircon analyses, thus reducing the inherent uncertainties in this correction procedure where there are large age differences between standard and sample zircons. Using this technique improved the accuracy and reproducibility of zircon U-Pb isotope analysis in our laboratory (See Tucker et al., 2013). For sample analysis, the total measurement time was set at 65 seconds. The first 30 seconds was for gas blank measurement (laser firing but with the shutter closed), with the shutter opened to allow sample ablation for the final 35 seconds, standard bracketing was used throughout the study to correct for remaining elemental fractionation and mass bias. Forty-eight grains were chosen for analysis using the optimized LA-ICP-MS tuning method outlined above, using a 32 μm beam diameter was used for sample analysis. The primary zircon standard GJ-1 (608.5 ± 0.4 Ma, Jackson et al., 2004) was used along with four different secondary standards, including Temora-2 [TEM-2] (416.8 Ma, Black et al., 2003), Plesovice (337.13 +-0.37 Ma, Sláma et al., 2008), Qinghu (159.5 +-0.2 Ma), and AusZ2 (38.8963+-0.00044 Ma) to monitor the accuracy and reproducibility of the dating. Following tuning of the instrument, each set of five unknown analyses were packaged between paired analyses of the both the primary and secondary zircons. All standard analyses were within 2% of the expected ages, and most were within 1% of the expected age. NIST 610 or 612 was analyzed at the beginning and end of each session, and at least once in between, for the purpose of calibrating Th and U concentrations.

Results

Data processing and reduction was performed using the Glitter software package (Van Achterbergh et al., 2001). All time-resolved isotope signals were filtered for signal spikes related to inclusions and fractures, and then background and sample intervals were evaluated to assess fractionation patterns and to identify the most stable and representative isotope ratios. U-Pb LA-ICP-MS zircon analyses were performed and all inherited older zircons and highly discordant analyses were filtered out prior to age calculation. Age calculations based on measured isotope ratios were completed using Isoplot/Ex version 4.15 and the Age7 function in isoplot for correction of common Pb was performed (Ludwig, 2012) (Table S1). $^{206}Pb/^{238}U$ ages are utilized because of the young age of the sample. A weighted mean age of 74.38 +-0.72 Ma (1σ) with an MSWD of 0.55 was calculated for DVTM-1 (Figure S1A). This is comparable with the lower intercept solution of 74.44 +-0.74 Ma (MSWD=0.56) for this sample (Figure S1B).
The age of DVTM-1 fits the stratigraphic superposition of the site and is consistent with published Ar/Ar dates for the Two Medicine in the type section (Rogers et al., 1993). Moreover, U-Pb detrital zircon geochronology of the Two Medicine Formation is ongoing in the study area (Roberts et al., 2011), and the maximum depositional ages documented for samples from the upper Two Medicine Formation in the study area are closely consistent with the age reported here for DVTM-1.

References

Black, L.P., Kamo, S.L., Allen, C.M., Davis, D.W., Aleinikoff, J.N., Valley, J.W., Mundil, R., Campbell, I.H., Korsch, R.J., Williams, I.S., Foudoulis, C., 2004. Improved Pb-206/U-218 microprobe geochronology by the monitoring of a trace-element-related matrix effect; SHRIMP,

ID-TIMS, ELA-ICP-MS and oxygen isotope documentation for a series of zircon standards. Chemical Geology 205, 115-140.

Jackson, S.E., Pearson, N.J., Griffin, W.L., Belousova, E.A., 2004. The application of laser ablation-inductively coupled plasma-mass spectrometry to in situ U–Pb zircon geochronology. Chemical Geology 211, 47-69.

Kennedy, A.K., Wotzlaw, J.F., Schaltegger, U., Crowley, J.L., Schmitz, M., 2014. Eocene zircon reference material for microanalysis of U-Th-Pb isotopes and trace elements. The Canadian Mineralogist 52: DOI:10.3749/canmin.52.3.409.

Li, X.H., Tang, G.Q., Gong, B., Yang, Y.H., Hou, K.J., Li, Q.L., Liu, Y., Li, Q.X., 2013, Qinghu zircon: A working reference for microbeam analysis of U-Pb age and Hf and O isotopes. Chinese Science Bulletin 58, 4647-4654.

Ludwig, K.R., 2012. User's Manual for Isoplot 3.75. A Geochronological Toolkit for Microsoft Excel: Berkley Geochronology Centre Special Publication No.5.

Roberts, E. M., Hendrix, M. S., and Jinnah, Z. A., 2011. Combining tephrostratigraphy with sandstone provenance and detrital zircon geochronoonology to assess along-strike continuity of alluvial sequence stratigraphic patterns in the Campanian Western Interior Basin, from western Montana to southern Utah: Geological Society of America Abstracts with Programs 43, 600A.

Scherzer, B.A., and Varricchio, D.J., 2010. Taphonomy of a juvenile Lambeosaurine bonebed from the Two Medicine formation (Campanian) of Montana, United States. Palaios 25, 780-795.
Sláma, J., Kosler, J., Condon, D.J., Crowley, J.L., et al., 2008. Plešovice zircon — A new natural reference material for U-Pb and Hf isotopic microanalysis. Chemical Geology 249, 1-35.

Tucker, R.T., Roberts, E.M., Hu, Y., Kemp, A.I.S., and Salisbury, S.W. 2013. Detrital zircon age constraints for the Winton Formation, Queensland: contextualizing Australia's Late Cretaceous dinosaur faunas. *Gondwana Research*, *24* 2: 767-779. doi:10.1016/j.gr.2012.12.009.

Van Achterbergh, E., Ryan, C.G., Jackson, S.E., Griffin, W.L. 2001. Data reduction software for LA-ICP-MS. In: P. Sylvester (Ed.), Laser-ablation-ICPMS in the Earth sciences: principles and applications. Mineral. Assoc. Canada, Short Course Series, vol. 29, p. 239-243.

6. Discussion S2. Diagnostic Characters of *Daspletosaurus horneri.*

A. We provide here a comparison of the diagnostic characters of *Daspletosaurus horneri* (sp. nov.) with the condition seen in other derived tyrannosauroids.

Wide dental arcade and laterally curved dentary—In *D. horneri* the rostral end of the snout appears to be widened relative to the condition seen in other derived tyrannosauroids, where the first interdental plate of maxilla is mesiodistally narrow and identical to the plates that separate the premaxillary teeth, and the second plate is truncated in having a vertical distal edge and a triangular mesial edge. This extreme difference in shape implies that the rostral end of the snout is widened to the side and the incisiform shape of the interdental plates is carried laterally. In other derived tyrannosauroids, the first plate is wide and truncated, and the second plate has the typical wide and pentagonal form of the rest of the plates of the dentary (*Albertosaurus libratus*, *A. sarcophagus*, *Alioramus remotus*, *D. torosus*). In some taxa, the first two plates are greatly expanded in size (*Tyrannosaurus bataar*, *T. rex*).

In concert with this widening of the upper jaw, the dentary in *D. horneri* when viewed from above is distinctly curved laterally in juveniles and adults. In contrast, the dentary is straight or only slightly curved when viewed dorsally in other taxa (*Albertosaurus libratus*, *A. sarcophagus*, *Alioramus remotus*, *Lythronax argestes*, *Teratophoneus curriei*, *D. torosus*, *Tyrannosaurus rex*). A strong curvature might be present in *T. bataar*.

Promaxillary sinus stopping between alveoli 3 and 4—In *D. horneri* the promaxillary sinus pinches out rostrally between the level of alveoli 3 and 4. In other derived tyrannosauroids, the sinus pinches out further rostrally, above alveolus 1 (*Albertosaurus libratus*, *A. sarcophagus*, *Raptorex kriegsteini*, *T. rex*), 2 (*D. torosus*), or 3 (*Alioramus remotus*).

Rostral end of the choana on the maxilla above alveolus 7—In *D. horneri* the rostral end of the choana in juveniles is positioned above alveolus 4 (AMNH FARB 5477). However, in subadult (MOR 590) and adult (MOR 1130) specimens it has a caudal position (above alveolus 7) when compared to other derived tyrannosauroids, where it is positioned above alveolus 4 (*Albertosaurus sarcophagus*) and 5 (*A. libratus*, *D. torosus*, *R. kriegsteini*. *T. rex*). Variation is seen in *T. rex*, where the rostral end of the choana is on occasion positioned above alveolus 9.

Inflated dorsal surface of the lacrimal not reaching the medial edge of the bone—In *D. horneri* the inflated dorsum of the lacrimal, opposite the cornual process stops short of the medial edge of the bone, where it flattens medially, level with the flat dorsal surface of the snout (prefrontal, nasal). This condition is also seen in *Alioramus remotus*. In contrast, the dorsum of the lacrimal is maximally inflated such that the convex surface reaches the medial edge of the bone in several derived tyrannosauroids (*Albertosaurus libratus*, *A. sarcophagus*, *D. torosus*, *T. rex*).

Medial pneumatic recess of the lacrimal tall and narrow slot—*D. horneri* is unusual among derived tyrannosaurines in that the medial pneumatic recess of the lacrimal is a narrow and small slot-like opening. In contrast, the recess is a large, round opening in its closest relatives (*Alioramus remotus*, *D. torosus*, *T. bataar*, *T. rex*). The opening is absent from *Bistahieversor sealeyi*, the *Albertosaurus* clade, and *Teratophoneus curriei*, whereas a rostromedially directed opening is seen in the Iren Dabasu taxon and *R. kriegsteini*.

Concave upper half of orbital margin of the lacrimal—In *D. horneri* the upper half of the orbital margin of the lacrimal follows a shallow concave curve. In contrast, this region is gently convex and flange-like in other derived tyrannosauroids (*D. torosus*, *Tyrannosaurus bataar*, *T. rex*). In other taxa, the margin is concave, but is interrupted by the large and convex suborbital

ligament scar (*B. sealeyi*, *Albertosaurus libratus*, *A. sarcophagus*, *Teratophoneus curriei*, *R. kriegsteini*).

Entire circumference of the pneumatic recess of the squamosal is undercut and clearly defined—The recess is entirely undercut in *D. horneri* and *Tyrannosaurus bataar*, whereas in other taxa at least one corner of the recess is not undercut, where the recess and the ceiling of the bone are joined together (*D. torosus*, *R. kriegsteni*, *T. rex*).

Sinuous rostral edge in dorsal view of the dorsotemporal fossa on the frontal—Although weathered in both mature specimens (MOR 590, MOR 113), the rostral margin of the dorsotemporal fossa in *D. horneri* is sinuous as it winds its way from the sagittal midline to the orbital notch. This condition is seen in other derived tyrannosauroids (*Albertosaurus libratus*, *L. argestes*, *R. kriegsteini*). In contrast, the margin is straightened in *D. torosus*. The margin is variable in *T. rex*, where it is might be sinuous, straightened, or rostrally convex, and it may extend rostrolaterally or caudolaterally from the midline. The rostrally convex condition is seen in *T. bataar*.

Joint surface for the squamosal on the parietal covers the base of the caudolateral process—In *D. horneri* the joint surface for the squamosal covers the base of the caudolateral process. This condition is also seen in *Albertosaurus libratus*, *A. sarcophagus*, and *T. rex*. In contrast, the joint surface covers only the ventral half of the base in other derived tyrannosauroids (*A. remotus*, *D. torosus*, *T. bataar*).

Tympanic ridge extends onto the prootic—In *D. horneri* the tympanic ridge that bounds the lateral tympanic space dorsally extends caudally onto the prootic such that it also bulges laterally above the caudal tympanic recess. This condition is also seen in *Albertosaurus libratus*, *A. sarcophagus*, *T. bataar* and *T. rex*. In contrast, the ridge is stout in *Alioramus remotus* and *D. torosus*, where it fades nearly immediately and does not continue onto the prootic as an eave-like structure.

Pneumatic foramen penetrating the lateral surface of the quadratojugal—This feature is seen in adult *D. horneri*, subadult *T. rex*, and and an unidentified taxon from the Dinosaur Park succession from southern Alberta. This foramen is not seen in Iren Dabasu taxon, *Bistahiversor sealeyi*, *Albertosaurus libratus*, *A. sarcophagus*, *Alioramus remotus*, *D. torosus*, or *T. bataar*.

Shallow notch between the basal tubera—The notch is relatively shallow in *D. horneri* (less than 40% the total height of the basioccipital below the occipital condyle. A shallow notch is also seen in *Albertosaurus libratus*. The tall condition, where the notch is greater than 40%, is seen in *D. torosus*. Variation is present in *T. rex* where both extremes in height are seen, which ranges from 21% to 45%). A tall notch is considered present in *Bistahieversor sealeyi*, *A. sarcophagus*, *Alioramus remotus*, and *T. bataar* (Bever et al., 2013).

Short epipophyses of the anterior cervicals—In *D. horneri*, the epipophyses of the anterior and mid cervicals are stout and project only a short distance past the postzygapophyses, if at all. This condition is also seen in *T. bataar* (Maleev, 1974). In contrast, the epipophyses are long and extend past the postzygapophyses in the anteriormost cervicals of *Albertosaurus libratus*, *Teratophoneus curriei*, and *Tyrannosaurus rex*.

The humerus is ~34% the length of the femur—In *D. horneri*, the humerus is one third (MOR 590) the length of the femur. A similar ratio is seen in *A. libratus* (TMP 1986.144.0001: 36%; CMN 2120: 31%; Lambe, 1917), *A. sarcophagus* (CMN 11315: 34%; Russell, 1970), and *Teratophoneus curriei* (BYU 8120/9396: 32%). The humerus to femur ratio of *D. torosus* is unknown owing to the absence of a femur from the holotype; so using the skull and ilium as a proxy for femur length, the ratio was in the range of 34% to 32%, respectively. Therefore, it may

turn out that the ratio of *D. horneri* is not unique once comparable bones are found in a *D. torosus* specimen. The bone is shorter in *Tyrannosaurus bataar* (PIN 552-1: 26%) and *T. rex* (FMNH PR2081: 29%; Brochu, 2003).

B. We list here, bone-by-bone, characters that distinguish large subadult and adult specimens of *D. horneri* from those of *D. torosus*. Characters seen only in *D. horneri* as listed first, followed by characters seen in *D. horneri* and other tyrannosaurids, except *D. torosus*.

Maxilla – unique *D. horneri* characters:

1. Promaxillary sinus stops between alveoli 3 and 4 (reaches alveolus 1 in *D. torosus*).
2. Rostral end of the choana on the maxilla is above alveolus 7 (stops above alveolus 5 in *D. torosus*).

Maxilla characters seen in *D. horneri* and other tyrannosaurids, except *D. torosus*:

1. The maxillary fenestra is as tall as long (also in *Bistahieversor*, *Albertosaurus libratus*, *Tyrannosaurus rex*; longer than tall in *D. torosus*).
2. Caudal end of trough medial to tooth root bulges is limited to the maxillary antrum (also in *A. libratus*, *Raptorex*; extends caudal to the antrum in *D. torosus*).
3. Jugal ramus diverges from the body of the maxilla several alveoli ahead of the last alveolus (also seen in *A. sarcophagus*, *Lythronax*; diverges at the last alveolus in *D. torosus*)
4. Joint surface for the palatine stops below the caudal edge of the postantral strut (also seen in *A. libratus*, *A. sarcophagus*, *Alioramus altai*, *Raptorex*, *T. rex*; extends below the strut in *D. torosus*).
5. Caudodorsal margin of the maxillary fenestra is convex (also seen in *Albertosaurus libratus*, *A. sarcophagus*, *Lythronax*, *Alioramus altai*, *Raptorex*, *T. rex*; straight or concave in *D. torosus*).

Lacrimal – unique *D. horneri* characters:

1. Inflated dorsal surface of the dorsal ramus of the lacrimal does not reach the medial edge of the bone (reaches the medial edge in *D. torosus*).
2. Medial pneumatic recess of the lacrimal is a tall and narrow slot (a large round opening in *D. torosus*).
3. Upper half of orbital margin is concave (convex in *D. torosus*).

Lacrimal characters seen in *D. horneri* and other tyrannosaurids, except *D. torosus*:

1. Angle between dorsal and ventral rami of the lacrimal is 80-90 degrees (also seen in *Bistahieversor*, *Albertosaurus libratus*, *A. sarcophagus*, *Alioramus altai*, *Teratophoneus*, *Raptorex*; less than 80 degrees in *D. torosus*).
2. Widest point across the lacrimal in dorsal view is caudal to the ventral ramus (also seen in *Tyrannosaurus rex*; widest point is above the ventral ramus in *D. torosus*).
3. Frontal process of the lacrimal is imperforate (also seen in *Alioramus altai*, *Raptorex*; perforate in *D. torosus*).
4. Dorsoventrally low cornual process of the lacrimal (also seen in *Albertosaurus libratus*, *A. sarcophagus*, *Teratophoneus*; tall in *D. torosus*).
5. The strut below the lacrimal pneumatic recess does not extend sharply rostromedially away from the ventral ramus (also seen in *Bistahieversor*, *Albertosaurus libratus*, *A. sarcophagus*, *Alioramus altai*, *Teratophoneus*, *Raptorex*, *Tyrannosaurus bataar*, *T. rex*; the strut extends sharply rostromedially in *D. torosus*).
6. Rostral ramus of the lacrimal is shorter than the ventral ramus (also seen in *Bistahieversor*, *Albertosaurus libratus*, *T. rex*; the reverse is seen in *D. torosus*).

Jugal—Characters seen in *D. horneri* and other tyrannosaurids, except *D. torosus*:
1. Inflation of jugal does not extend below the jugal pneumatic recess (also seen in *Alioramus altai*, *Raptorex*, and in some specimens of *Albertosaurus* spp. and *T. rex*; extends below the recess in *D. torosus*).
2. Distance between the split between the quadratojugal processes of the jugal and the point where the joint surface for the quadratojugal process extends off the ventral margin of the bone is long (also seen in *Albertosaurus libratus*, *A. sarcophagus*, *Alioramus altai*, and in some specimens of *T. rex*; distance is short in *D. torosus*).
3. Ventral half of the joint surface for the quadratojugal joint surface is positioned lateral to the dorsal quadratojugal process (also seen in *Albertosaurus libratus*, *A. sarcophagus*, and in some specimens of *T. rex*; positioned medial to the process in *D. torosus*).
4. Proximal end of the dorsal quadratojugal process of the jugal is horizontally oriented (seen in some *T. rex* specimens; low caudodorsal angle in *D. torosus*).
5. Lateral maxillary process of the jugal is blocked from view medially (also seen in *A. libratus*, *A. sarcophagus*, and in some specimens of *T. rex*; exposed to medial view in *D. torosus*).
6. Caudal edge of the antorbital fossa extends rostrodorsally from the jugal pneumatic recess (also seen in some *A. libratus* and *A. sarcophagus* specimens, *Alioramus altai*, *Raptorex*, *T. rex*; extends caudodorsally in almost all *D. torosus* specimens).

Postorbital—Characters seen in *D. horneri* and other tyrannosaurids, except *D. torosus*:
1. Dorsal margin of the squamosal process is gently concave (also seen in *Bistahieversor*, *Albertosaurus libratus*, *A. sarcophagus*, and in some specimens of *T. rex*; margin is convex in *D. torosus*).
2. Spur above the joint surface for the jugal in lateral view is absent (also seen in *A. libratus*, *Alioramus altai*, and some specimens of *T. rex*; present in *D. torosus*).

Squamosal – unique *D. horneri* characters:
1. The entire circumference of the pneumatic recess is undercut and clearly defined (rostromedial margin is not undercut in *D. torosus*).
2. Rostral edge of the dorsotemporal fenestra is sinuous in dorsal view (concave in *D. torosus*).
3. Medial process of the bone reaches the laterosphenoid (does not reach the laterosphenoid in *D. torosus*).

Squamosal characters seen in *D. horneri* and other tyrannosaurids, except *D. torosus*:
1. Strut medial to the pneumatic recess is narrow (also seen in *Alioramus altai*, *Teratophoneus*, and in some *Tyrannosaurus rex* specimens; wide in *D. torosus*).
2. A flange extends lateroventrally from the ventral postorbital process (also seen in *A. altai*, subadult *T. rex*; flange is absent from *D. torosus*).
3. Tip of the quadratojugal ramus is dorsoventrally shallow in lateral view (also seen in *Bistahieversor*, *Albertosaurus libratus*, *A. sarcophagus*, subadult *T. rex*; deep in *D. torosus*).
4. Dorsal margin of the joint surface for the quadratojugal is stabilized by a slot (also seen in some *T. rex* specimens; slot is absent from *D. torosus*).

Quadratojugal—Characters seen in *D. horneri* and other tyrannosaurids, except *D. torosus*:
1. Large foramen penetrates the lateral surface of the shaft (seen in subadult *T. rex*; not seen in *D. torosus*).

2. Caudal margin of the ventral quadrate process is not notched (also seen in most *T. rex* specimens; deeply notched in *D. torosus*).
3. Upper half of rostral margin of the dorsal joint surface for the quadrate is gently concave (also seen in *Alioramus altai*, and in some specimens of *T. rex*; gently convex in *D. torosus*).
4. Ventral margin of the jugal process extends sharply rostrodorsally in lateral view (also seen in *Albertosaurus sarcophagus*; does not abruptly extend rostrodorsally in *D. torosus*).
5. Only the proximal part of the dorsal joint surface for the jugal can be seen on the jugal process in lateral view (also seen in *A. sarcophagus*, and in some specimens of *T. rex*; the entire joint surface can be seen in *D. torosus*).
6. Concavity is present between jugal process and ventral quadrate process in lateral view (also seen in *Alioramus altai*, and in some *T. rex*; absent from *D. torosus*).

Quadrate—Characters seen in *D. horneri* and other tyrannosaurids, except *D. torosus*:
1. Medial fossa of the orbital process is deep and the caudoventral corner is undercut (also seen in *Albertosaurus sarcophagus*, subadult *T. rex*; fossa is shallow and the caudoventral corner is not undercut from *D. torosus*).
2. Rostral margin of the orbital process extends at a steep rostroventral angle (also seen in *Alioramus altai*, *Teratophoneus*, and in some specimens of *Tyrannosaurus rex*; extends at a low rostroventral angle in *D. torosus*).
3. Ventral margin of the orbital process extends rostrodorsally along a steep angle (also seen in *Albertosaurus sarcophagus* and in some *T. rex* specimens; low angle in *D. torosus*).
4. Rostroventral margin of the orbital process extends along a steep 45-degree angle (also seen in *A. sarcophagus*, *Alioramus altai*; extends along a low angle in *D. torosus*).
5. A groove does not separate the medial mandibular condyle from the mandibular process medially in caudal view (also seen in *T. rex* and in some *Albertosaurus sarcophagus* specimens; groove separates the condyle laterally in *D. torosus*).
6. Dorsal extent of the pneumatic recess extends onto the medial surface of the orbital process as a shallow fossa (also seen in *A. sarcophagus*, *Alioramus altai*, and in some *T. rex* specimens; recess does not extend onto the orbital process in *D. torosus*).
7. Maximum length of the mandibular process is through the medial half of the lateral condyle in ventral view (also seen in some *T. rex* specimens; lateral half of the medial condyle in *D. torosus*).
8. Subordinate pneumatic fossae are present rostral to the pneumatic recess (also seen in some *T. rex* specimens; absent from *D. torosus*).

Frontal—Frontal characters seen in *D. horneri* and other tyrannosaurids, except *D. torosus*:
1. Sagittal foramen is located opposite the midlength of the postorbital buttress (also seen in *Nanuqsaurus*; located at the level of the transition between the postorbital buttress and the caudal shelf in *D. torosus*).
2. Subcutaneous surface of the interorbital region reaches the sagittal foramen (also seen in *Nanuqsaurus* and subadult *T. rex*; subcutaneous surface and foramen are separated by the dorsotemporal fossa in *D. torosus*).
3. The orbital surface extends at a low laterodorsal angle in lateral view (also seen in *Albertosaurus libratus*, *A. sarcophagus*, *Teratophoneus*, *Nanuqsaurus*, *Raptorex*, and in some *Tyrannosaurus rex* specimens; steep angle in *D. torosus*).

4. Frontals are separated caudally on the midline by a prominent wedge of the parietal (also seen in *A. libratus*, *A. sarcophagus*, *Nanuqsaurus*, *Raptorex*, *Alioramus altai*, and in some *T. rex* specimens; wedge is not as rostrally long in *D. torosus*).

Parietal – unique *D. horneri* characters:
1. Joint surface for the squamosal is deeply excavated and excavates the bone above the caudolateral process (joint surface does not excavate the bone above the caudolateral process in *D. torosus*).

Parietal characters seen in *D. horneri* and other tyrannosaurids, except *D. torosus*:
1. The parietal overlaps the frontals rostrally (also seen in some *T. rex* specimens; contact is abutting and the frontal overlaps the parietal in *D. torosus*).
2. Midline strut on caudal surface bifurcates before contacting the supraoccipital (might be present in some *T. rex* specimens; strut does not bifurcate in *D. torosus*).

Braincase – unique *D. horneri* characters:
1. Caudal surface of the ventral plate of the basioccipital is deeply concave (shallowly concave in *D. torosus*).
2. Tympanic ridge extends onto the prootic (does not reach the prootic in *D. torosus*).
3. Dorsomedial to ventrolateral ridge crosses the orbital surface of the laterosphenoid (absent from *D. torosus*).

Braincase characters seen in *D. horneri* and other tyrannosaurids, except *D. torosus*:
1. Tip of tuberal crest does not reach the oval scar complex (also seen in *Teratophoneus*, and some *Tyrannosaurus rex* specimens, almost certainly seen in *Alioramus altai*; excavated by the oval scar in *D. torosus*).
2. Basioccipital contribution to the basisphenoid recess is limited to the midregion (also seen in *Albertosaurus libratus*, *A. sarcophagus*, *Alioramus altai*, and in some *T. rex* specimens; forms the caudolateral corner of the recess in *D. torosus*).
3. In caudal view the basisphenoid extends laterally as a tab like process beside the basal tuber (also seen in *T. rex*; tab like process is absent from *D. torosus*).
4. CNs III and IV exit the laterosphenoid separately (also seen in *Albertosaurus sarcophagus*, *Alioramus remotus* (Kurzanov, 1974), and in most *T. rex* specimens; exit from a common fossa in *D. torosus*).
5. Foramen of CN IV is much smaller than that of CN III (also seen in *T. rex*; the reverse is seen in *D. torosus*).
6. Dorsal half of the laterosphenoidoprootic suture forms a ridge (also seen in *T. bataar* and in some *T. rex* specimens; flat in *D. torosus*).
7. Laterosphenoidoprootic stuture is caudally convex (also seen in some specimens of *Albertosaurus sarcophagus* and *T. rex*; sigmoid in *D. torosus*).
8. The external surface at the laterosphenoidoparietal suture is convex (also seen in *A. sarcophagus* and *T. bataar*, and in some specimens of *Albertosaurus libratus* and *T. rex*; concave in *D. torosus*).

Palate—Characters seen in *D. horneri* and other tyrannosaurids, except *D. torosus*:
1. The dorsal ramus of the palatine in subadults extends dorsally (also seen in *Albertosaurus libratus*; extends rostrodorsally in *D. torosus*).

2. Lateral flange of the palatine is positioned below the rostral end of the caudal pneumatic recess (also seen in *Alioramus altai* and in some specimens of *T. rex*; positioned caudal to the rostral end of the recess in *D. torosus*).
3. Rostral pneumatic recess is deeply excavated (also seen in *Albertosaurus libratus*, *A. sarcophagus*, *Alioramus altai*, some specimens of *T. rex*; shallow in adults of *D. torosus*).
4. Pneumatic recesses are widely separated from each other (also seen in *Alioramus altai*, some *T. rex* specimens; close together or overlapping in *D. torosus*).
5. Caudal pneumatic recess exceeds the height of the maxillary ramus (also seen in *Bistahieversor*, and some specimens of *Albertosaurus sarcophagus* and *T. rex*; at most is as tall as the ramus in *D. torosus*).
6. The rostral and caudal pneumatic recesses of the palatine are connected to each other internally (also seen in *Alioramus altai* and in some specimens of *T. rex*; separated by a partition in *D. torosus*).
7. Pneumatic recesses that penetrate the medial surface of the palatine are absent (also seen in *Albertosaurus libratus*, *A. sarcophagus*, *Alioramus altai*, some specimens of *T. rex*; present in *D. torosus*).
8. Joint surface on the caudolateral process of the palatine extends rostrally below the caudal pneumatic recess (also seen in *Albertosaurus sarcophagus*, *Alioramus altai*; stops far caudal to the recess in *D. torosus*).
9. One pneumatic recess is present in the ectopterygoid (also seen in *Alioramus altai*, *T. bataar*, and in some specimens of *T. rex*; two are present in *D. torosus*).
10. Caudal process of the ectopterygoid is penetrated by an accessory pneumatic foramen (seen in some specimens of *T. rex*; absent from *D. torosus*).

Mandibular ramus – unique *D. horneri* characters:
1. A wide shelf separates the joint surface for the angular from the caudal surangular foramen (shelf is absent from *D. torosus*).
Mandibular characters seen in *D. horneri* and other tyrannosaurids, except *D. torosus*:
1. Surangular shelf does not overhang the caudal surangular foramen (also seen in *Albertosaurus libratus*, *Alioramus altai*, *Raptorex*, seen in some *T. rex* specimens; overhangs the foramen in *D. torosus*).
2. Caudal surangular foramen does not extend to the ventral surface of the surangular shelf (also seen in *Albertosaurus libratus*; foramen reaches the shelf in *D. torosus*).
3. Dorsal margin of the joint surface for the intercoronoid reaches the ventral margin of the medial bar (also seen in *A. libratus*, *Raptorex*, *T. bataar*, and in some specimens of *T. rex*; dorsal margin fades before reaching the ventral margin in *D. torosus*).
4. The joint surface for the angular extends behind the level of the caudal surangular foramen (also seen in *Bistahieversor*, *A. libratus*, *Lythronax*, *Alioramus remotus*, *A. altai*, *Raptorex*, *T. rex*; stops at the caudal edge of the foramen in *D. torosus*).
5. Seventeen (17) dental alveoli (also seen in subadult *T. rex*; 16 in *D. torosus*).

7. Discussion S3. Discussion of the Superficial Craniomandibular Soft Tissues of Tyrannosaurids

Goals & Methods. The excellent quality of preservation of these specimens (MOR 553S/7.19.0.97, MOR 590, MOR 1130) permits us to assess the type of soft tissue that covered the face (premaxilla, maxilla, nasal, lacrimal, jugal, postorbital, squamosal, dentary). In *D. horneri*, and in all derived tyrannosauroids (*Bistahieversor* + Tyrannosauridae), the subcutaneous texture is coarse and shows a hierarchy of textures. In order to identify the soft tissue covering that produced this complex surface, we compared the condition of tyrannosaurids with that of crocodylians (*Alligator*) and birds (*Struthio*, *Anser*, *Anas*, *Cygnus*), and we applied the results of Hieronymus et al. (2009) and Hieronymus and Witmer (2010) to deduce the soft tissues that produced the osteological features.

Snout & orbital region. In tyrannosaurids, large neurovascular foramina penetrate the subcutaneous surface from which deep, sharply inset sulci extend. Based on comparisons with crocodylians and birds, the foramina that emerge onto the lateral surface of the snout and jaws correspond to: the sensory dorsal and ventral branches of the medial ophthalmic nerve (N. V_1), internal maxillary artery, maxillary division of trigeminal nerve (N. V_2), dorsal alveolar vessels and nerve, ventral subnarial vessels and nerve, lateral paravestibular neurovascular bundle, lateral premaxillary neurovascular bundle, rostral premaxillary neurovascular bundle, and median palatine artery (Papp, 2000; Sedlmayr, 2002). The openings that emerge through the dorsum of the snout correspond to the lateral nasal and medial nasal vessels and twigs of the ophthalmic ramus of the trigeminal nerve (N. V_1) (Sedlmayr, 2002).

In form, the foramina and their sulci correspond to a tangential rugosity profile (Hieronymus et al., 2009), where the foramina are oriented oblique to the external surface of the bone. The grooves usually bifurcate several times as they extend across the bone, producing wide swaths and series of V-shaped grooves, which produce a hummocky rugosity profile between them (Hieronymus et al., 2009). The grooves tend to be ordered and anastomosing in orientation, and they are numerous and closely spaced.

On top of the primary texture there is an overprint of fine grooves and ridges and, finally, a tertiary texture coarsens the fine ridges. These correspond to a projecting rugosity profile (Hieronymus et al., 2009). In some areas of the skull (nasals, lacrimals, maxillae) the secondary and tertiary textures are elaborated into large, coarse ridges, cusps, and papillae.

The texture has a sharp boundary with the smooth temporal region on the skull that is marked by a raised lip on the postorbital, whereas the transition from coarse to smooth is gradational and without relief on the lower jaw. As such, the coarse texture is limited to the rostral part of the facial skeleton ahead of the temporal region. Tyrannosaurids bear three or four pairs of ornamental horns, which occur within the boundaries of the coarse region. The surface of the temporal region is generally smooth or textured by fine ridges and grooves.

Although tyrannosaurids, crocodylians, and birds share neurovascular foramina that are densely clustered at the front of the jaws and form rows along the oral margins, a sharp difference in texture separates birds from their closest relatives. The texture in crocodylians is identical to that of tyrannosaurids, except that the entire face of crocodylians is coarse in texture. In contrast, the texture deep to the beak in birds is smooth, aside from sharply incised neurovascular sulci. The osteological texture between the primary neurovascular sulci of tyrannosaurids compares best with the hummocky texture that is consistent with overlying scales as is seen in living crocodylians (Hieronymus et al., 2009).

Postorbital bone. In tyrannosaurids a lip-like transition is seen on the postorbital between the textured and nontextured surfaces; this abrupt, stepped transition indicates the presence of a cornified sheath (Hieronymus et al., 2009) on at least the postorbital that graded rostrally into the scaly covering on the rest of the face. A localized sheath is supported by the observation in *Tyrannosaurus* (AMNH FARB 5027, MPC-D 107/2) where a pair of osteoderms are situated above the orbit that fuse to the underlying bones; osteoderms are associated with overlying scales (Hieronymus et al., 2009) and therefore the caudal margin of the caudal osteoderm marks the rostral limit of the cornified sheath. Should similar osteoderms be seen in other tyrannosaurids then the rostral limit of the sheath can be identified.

The cornual process of the postorbital in *Daspletosaurus* and other tyrannosaurids often has a coarse and warty dorsal and caudal surface, and a smooth lateral surface. This arrangement indicates the presence of a projecting skin structure such as a horn (Hieronymus et al., 2009). Indeed, the undercut caudal and dorsal edge of the cornual process (which extends rostrally along the frontal process) corresponds to the lip-like edge of bony structures that support cornified sheaths (Hieronymus et al., 2009). Based on these lines of evidence, we conclude that at least part of the postorbital cornual process of tyrannosaurids supported a cornified sheath.

Nasal bone. Tyrannosaurids bear a series of low bumps along the dorsal midline of the nasal bones; these bumps almost certainly correspond to individual overlying scales, as is seen in centrosaurine ceratopsians (Hieronymus et al., 2009). In some tyrannosaurids (*Alioramus remotus*), the bumps are quite tall, but they do not possess the basal rims that indicate a cornified sheath; therefore, those bumps also mark the position of overlying scales. In *D. horneri* and other tyrannosaurids, the nasal rugosity increases with maturity (see 'Armor-like dermis' below), but then it becomes smooth in the most mature specimens. A similar ontogenetic process is seen in centrosaurines, from supraorbital horns to supraorbital pits, but that change reflects the transition from a cornified sheath to a cornified boss; the process in tyrannosaurids does not show evidence for a change in the overlying epidermal tissue since the difference is only a decrease in relief.

Gingiva. Texture is also informative regarding the soft tissues that covered the oral margins of tyrannosaurids. The foramina along the margin of the jaws emit stout (upper jaw) or long (lower jaw) sulci that often fade before reaching the oral margin. This arrangement produces a smooth zone between the alveolar foramina and the edge of the jaw, which is also seen in crocodylians. In tyrannosaurids vertical rows of minute foramina extend perpendicular to the tooth row in the smooth zone. These subordinate rows of foramina are also seen in crocodylians and in mammals (*Equus*); which may be an osteological correlate of gingiva for Amniota.

Rhamphotheca. Birds are similar to tyrannosaurids and crocodylians in that the surface deep to the beak is penetrated by large foramina that emit sharp-edged, long and branching sulci, but an overprint of a secondary or tertiary texture is not seen and the beak-bearing surface is otherwise smooth (Papp, 2000; Hieronymus et al., 2009). Also, externally the foramina are low in number and are concentrated at the rostral and caudal extremes of the upper jaw (Papp, 2000). Therefore, we reject the hypothesis that tyrannosaurids bore beaks, and we fail to reject the hypothesis that their faces had a scaly covering comparable to crocodylians, a cornified sheath on the postorbital, and armor-like dermis on the lacrimal, nasal, and dentary (see below).

In addition to a coarse subcutaneous texture, tyrannosaurids and crocodylians share other features that are not seen in birds; these include neurovascular foramina that penetrate through the top of the snout, and the caudalmost foramen of the alveolar row of the maxilla is large and emits a long, deeply incised, and caudally extending sulcus that excavates the side of the

caudalmost tip of the bone. These differences from birds almost certainly reflect a lack of specialized blood supply to the snout, the large size of the jaws, and presence of teeth.

Osteological correlates of rhamphothecal plates, such as the bony groove deep to the nasolabial groove along the premaxillomaxillary suture that is seen in modern birds and the extinct Cretaceous birds *Hesperornis* and *Ichthyornis* (Hieronymus and Witmer, 2010), are not seen in tyrannosaurids or crocodylians. On the lower jaw, the mentolabial groove is a correlate in birds that marks the separation between the plates of the rhamphotheca of the lower jaw; this correlate is not seen in tyrannosaurids, crocodylians, or *Velociraptor* (AMNH FARB 6515), but it is seen in the extinct birds *Hesperornis* and *Ichthyornis* (Hieronymus and Witmer, 2010).

Papp (2000) identified osteological correlates for the presence of a rhamphotheca, namely numerous neurovascular foramina and sulci, and sulci, ridges (e.g., tomial ridges), or knobs within the beak region. Although numerous neurovascular foramina are seen in crocodylians and nonavian theropods, the associated sulci, ridges, and knobs are not. We emphasize here that the rugose sculptured texture on the ventral surface of the dentary by itself is not evidence for a rhamphotheca since a beak is absent from crocodylians and some nonavian dinosaurs, whereas beaks are seen in birds and some nonavian dinosaurs (Papp, 2000).

We therefore conclude that rhamphothecae were absent from nonavian theropods, if a compound rhamphotheca is the primitive condition (Hieronymus and Witmer, 2010). Also, the groove that unites the primary alveolar row of foramina in the dentary of tyrannosaurids, *Velociraptor*, and modern birds, is not a correlate of the infralabial groove, which is a novel feature limited to cassowaries, anatoideans, and some procellariiformeans (Hieronymus and Witmer, 2010). It therefore appears that the compound rhamphotheca evolved between the divergence of deinonychosaurians and the appearance of *Hesperornis*.

Alveolar groove. In crocodylians, nonavian theropods, and birds, a primary row of foramina extends along the lateral surface of the dentary parallel to the dorsal edge of the bone. These foramina correspond to the sensory intramandibular branch of the mandibular nerve (N. V_3), branches of the external cutaneous vessels, and the ventral alveolar artery (Papp, 2000; Sedlmayr, 2002). As in crocodylians, multiple rows of foramina are seen on the lateral surface of the dentary, including the ventrolateral surface; this is unlike birds, in which only a single lateral row is seen, aside from the rostral tip of the lower jaw (Papp, 2000).

Tyrannosaurids are similar to birds in that the alveolar row of foramina in the dentary occurs in a common groove that extends for much of the length of the bone; this common groove is not seen in crocodylians, although long, caudodorsally extending sulci extend from the foramina. In birds this groove is covered by the rhamphotheca, and the ventral rictal vessels and the external branch of the mandibular nerve lie in the groove (Sedlmayr, 2002); we regard the groove as a possible osteological correlate for those structures.

In tyrannosaurids the groove is not tangential, whereas it is undercut in more highly derived, bird-like theropods such as *Velociraptor* and birds (*Struthio*, *Anser*, *Anas*); we therefore suggest that the dentary of tyrannosaurids was covered by epidermal scales such as is seen in crocodylians. Ergo, the groove is an osteological correlate of the neurovasculature and not of the overlying skin, and indicates that this change to the jaw supply preceded the evolution of the rhamphotheca. The presence of a deep groove in *Velociraptor* suggests that the rhamphotheca had first evolved on the mandible before the upper jaw, and beaked jaws arose long before tooth loss. In texture the subcutaneous surface of the dentary in tyrannosaurids is identical to that of the upper jaw (but see below), which is consistent with the hypothesis of a scaly epidermis.

Armor-like dermis. In subadult and adult tyrannosaurids, the rostral surface of the premaxilla and the lateral surface of the dentary at the rostral half of the bone is coarsened by small pustule-like bumps. This texture is associated with armor-like dermis (Hieronymus et al., 2009), which would have protected against abrasion and scrapes while eating a carcass. This papillate texture is also seen on the dorsolateral surface of the lacrimal, which often bears a horn or swelling and indicates the presence of armor-like dermis and not a superimposed scale. Also, rugose 'burrs' and papillae are not infrequently seen on the coarse nasal bones of tyrannosaurids, which might mark localized 'warts' of armor-like dermis on the top of the snout.

Summary. In conclusion, we hypothesize that the coarse region of the face (upper and lower jaws) in tyrannosaurids was covered by flat epidermal scales as is seen in crocodylians. Unlike crocodylians, tyrannosaurids have a variety of other epidermal structures that include armor-like dermis on the cornual processes of the jugal and lacrimal, the top of the snout, and side of the lower jaw, and cornified epidermis on the postorbital cornual process. These hypotheses of epidermal identity can be tested with a microscopy-based examination for their corresponding histological correlates (Hieronymus et al. 2009) and by the discovery of fossils that preserve craniofacial integument either as fossilized skin, or, as natural molds or casts.

8. Discussion S4. Growth Series

We compared the growth series with the hierarchy of tyrannosaurine synapomorphies to assess the presence of recapitulation of phylogenetic characters during growth. We found that characters at the level of Tyrannosaurinae and the *Daspletosaurus* + (*Zhuchengtyrannus* + *Tyrannosaurus*) clade appear without congruence throughout the growth series. This noncongruence between growth and phylogeny almost certainly reflects the incomplete condition of the subadult specimens (represented by single bones) and the significantly more complete skulls of the mature specimens.

9. Phylogenetic Character List
Listed here are the phylogenetic characters, with descriptions of their states, which were used to resolve the ingroup phylogenetic relationships of Tyrannosauroidea. Much of the wording is unaltered from Brusatte and Carr (2016), but only essential notes have been retained. Characters that are new in this study are identified in boldface and start with the word "new"; the abbreviation "var." indicates character descriptions that are modified from their original source. Red text indicates departures from the wording of Brusatte and Carr (2016).

GENERAL FEATURES (1 character)
1) Body, size: small, where the skull, femur, or ilium is less than 750 mm long (0); large, where the skull, femur, or ilium is greater than 750 mm in length (1) (var. Carr and Williamson, 2010:273. Note: *Alioramus remotus* and *A. sinensis* were coded based on femur length (which are less than 750 mm), given that their skulls are apomorphically long (Lu et al., 2014). ORDERED

GENERAL SKULL FEATURES (9 characters)
2) Skull, occipital region, orientation, caudal view: caudal (0), caudoventral (1) (Holtz, 2001:65; Currie et al., 2003:63; Carr and Williamson 2010:3).

3) Skull, general shape, lateral view: long and low, length to height ratio greater than 3.2 (0); deep, length to height ratio less than 3.2 (1). Note: Length is premaxilla to quadrate condyle length; height is maximum height of the upper jaw, not counting any cranial crests.

4) Skull, rostrocaudal length, lateral view: less (0) or greater than (1) 40% trunk length (Sereno et al. 2009). Trunk length is from the anterior extremity of the pectoral girdle to the posterior extremity of the pelvic girdle (Sereno et al., 2009).

5) External naris, length relative to skull length, lateral view: less (0) or greater than (1) 20% of skull length. For incomplete skulls: the length of the bony naris is significantly less than that of the internal antorbital fenestra (0) or approximately the same length (1) (Carr and Williamson 2010:54; Brusatte and Carr, 2016:4).

6) Skull, bony naris, position of caudal margin, lateral view: rostral to external antorbital fenestra (0); caudal to or level with the rostral margin of the external antorbital fenestra, but rostral to the maxillary fenestra (1); caudal to the rostral margin of the external antorbital fenestra, and caudal to the rostral margin of the maxillary fenestra (2) (Carr and Williamson, 2010:53; Loewen et al., 2013:2; Brusatte and Carr, 2016:323). ORDERED.

7) Orbital fenestra, shape, lateral view: round (0); more than 10% taller dorsoventrally than long rostrocaudally, but less than twice as tall as long (1); dorsoventrally tall, more than twice as tall as long (2) (Loewen et al., 2013:4; Brusatte and Carr, 2016:324). ORDERED.

8) Orbital fenestra, size, lateral view: large, minimum rostrocaudal length at least three times the minimum rostrocaudal length of the laterotemporal fenestra (0); small, minimum rostrocaudal length less than three times the minimum rostrocaudal length of the laterotemporal fenestra (1) (Loewen et al., 2013:5; Brusatte and Carr, 2016:325).

9) Orbital fenestra, orientation, dorsal view: laterally directed, rostrocaudal axis of the fenestra is parallel to the sagittal plane (0); rostrolaterally directed, orbit is directed rostrally at greater than 20 degrees from the sagittal plane (1) (Holtz, 2001:105; Loewen et al., 2013:6; Brusatte and Carr, 2016:326).

10) Laterotemporal fenestra, orientation of long axis relative to long axis of orbit, lateral view: caudodorsal (0); approximately parallel (1) (Brusatte et al., 2010:5; Loewen et al., 2013:8; Brusatte and Carr, 2016:5). Note: the score of *Lythronax* and *Teratophoneus* score is changed from ? to 1 in light of new information from Loewen et al (2013). The orientation of the postorbital process of the jugal provides sufficient information with which to score this character, where a vertical process indicates a parallel orientation for the fenestra and a caudodorsally extending process indicates a nonparallel orientation.

PREMAXILLA (9 characters)

11) Premaxilla, nasal processes of opposing premaxillae, orientation, dorsal view: divergent from each other, with small process of nasals fitting in between them (0); apposed to each other to their tips (1) (Carr, 1999; Holtz, 2001:106; Currie et al., 2003:51; Carr and Williamson 2010:5; Loewen et al., 2013:17; Brusatte and Carr, 2016:6).

12) Premaxilla, deep foramen or fossa in the base of the nasal process, within the rostroventral corner of the narial fossa, lateral view: absent (0), present (1) (Carr and Williamson, 2010:6; Loewen et al., 2013:19; Brusatte and Carr, 2016:7).

13) Premaxilla, main body, dorsoventral height, lateral view: less than or equal to (0), between 1-1.9 times (1), greater than 2 times (2) rostrocaudal length (Sereno et al., 2009:1; Carr and Williamson, 2010:7; Brusatte et al., 2010:8; Loewen et al., 2013:22; Brusatte and Carr, 2016:8). ORDERED.

14) Premaxilla, maxillary process, orientation, lateral view: mostly laterally (and resultantly widely visible in lateral view) (0), dorsolaterally (facing almost equally dorsally and laterally) (1), dorsally (and resultantly mostly hidden in lateral view) (2) (Carr and Williamson 2010:9; Brusatte et al., 2010:9; Loewen et al., 2013:14; Brusatte and Carr, 2016:9). ORDERED.

15) Premaxilla, orientation of tooth row, ventral view: strongly parasagittally (rostrocaudally) (0); first two teeth oriented mediolaterally and third and fourth teeth oriented parasagittally (1); entire tooth row oriented mediolaterally and all teeth visible in rostral view (2) (Holtz, 2001:2; Currie et al., 2003:52; Carr and Williamson 2010:11; Brusatte et al., 2010:10; Loewen et al., 2013:13; Brusatte and Carr, 2010:10). ORDERED.

16) Premaxilla, narial fossa ventral to bony naris, form, lateral view: shallowly excavated (0); deeply excavated, rostral margin invaginated as a deep groove (1) (Brusatte et al., 2010:11; Loewen et al., 2013:20; Brusatte and Carr, 2016:11).

17) Premaxilla, narial fossa, extent, lateral view: limited to region immediately ventral to bony naris (0); extensive, covers most of main body of premaxilla (1) (Brusatte et al., 2010:12;

Loewen et al., 2013:21; Brusatte and Carr, 2016:12).

18) Premaxilla, rostral margin, shape, lateral view: smoothly curved and extending caudodorsally, angle between ventral margin of premaxilla and rostral margin is less than 90 degrees (0); smoothly curved and extending vertically or slightly rostrodorsally, angle between ventral margin of bone and rostral margin is equal to or greater than 90 degrees (1); oriented vertically or slightly rostrodorsally, with a distinct inflection point between nearly vertical rostral region and more horizontal dorsal region (2) (Carr and Williamson, 2010:4; Brusatte et al., 2010:13; Loewen et al., 2013:15, 16; Brusatte and Carr, 2016:13). Note: code for *D. horneri* is changed from 2 to ?. ORDERED.

19) Premaxilla, palatal process, dorsoventral position, medial view: immediately above interdental plates (0), separated from interdental plates by deep lingual surface of premaxilla (1) (Brusatte et al., 2010:14; Loewen et al., 2013:24; Brusatte and Carr, 2016:14).

MAXILLA (33 characters)

20) Maxilla, promaxillary fenestra, exposure, lateral view: visible (0), concealed by the lateral lamina of the ascending ramus (fenestra faces completely rostrally) (1) (Russell, 1970; Witmer, 1997; Holtz, 2001:40; Currie et al., 2003:35; Carr and Williamson 2010:13; Brusatte et al., 2010:15; Brusatte and Carr, 2016:15).

21) Maxilla, promaxillary fenestra, dorsoventral position, lateral view: rostral margin of antorbital fossa (0), extreme rostroventral corner of antorbital fossa (1) (var. Holtz, 2001:93; Brusatte et al., 2010:16; Brusatte and Carr, 2016:16).

22) Maxilla, maxillary fenestra, rostrocaudal position, lateral view: caudal to (0) or partially overlapped laterally by (1) the rostral margin of the antorbital fossa (=lateral lamina of maxilla) (Holtz, 2001:42; Currie et al., 2003:30; Sereno et al. 2009: 12; Carr and Williamson 2010:14, 15; Brusatte et al., 2010:17; Loewen et al., 2013:35; Brusatte and Carr, 2016:17).

23) Maxilla, maxillary fenestra, dorsoventral position, lateral view: dorsal to (0) or abuts (1) ventral margin of antorbital fossa (Sereno et al. 2009:13; Carr and Williamson, 2010:17; Brusatte et al., 2010:18; Loewen et al., 2013:36; Brusatte and Carr, 2016:18).

24) Maxilla, maxillary fenestra, rostrocaudal length compared to the distance between the rostral margins of the antorbital fossa and fenestra, lateral view: less than half (0), greater than half (1), greater than half and also greater than half of the length of the eyeball-bearing portion of the orbit (2) (var. Holtz, 2001:41; Currie et al., 2003:31; Sereno et al. 2009:11; Carr and Williamson 2010:16; Brusatte et al., 2010:19; Brusatte and Carr, 2016:19). ORDERED.

25) NEW Maxilla, maxillary fenestra, size in juveniles compared to the condition seen in adults, lateral view: large (0), small (1).

26) Maxilla, maxillary fenestra, ratio of maximum rostrocaudal length to maximum dorsoventral height, lateral view: less than (0) or greater than (1) 1.9 (Lu et al., 2014: 316; Brusatte and Carr, 2016:314).

27) Maxilla, maxillary fenestra, dorsoventral position within maxillary antrum, medial view: does not (0) or does (1) abut dorsal border of the antrum in medial view (Brusatte et al., 2010:20; Brusatte and Carr, 2016:20).

28) Maxilla, maxillary fenestra, caudal margin, shape, lateral view: rounded (0); V-shaped (1) (Brochu, 2003; Loewen et al., 2013:34; Brusatte and Carr, 2016:327).

29) Maxilla, secondary fossa above maxillary fenestra within the external antorbital fenestra, presence, lateral view: absent (0), present (1) (Loewen et al., 2013:38; Brusatte and Carr, 2016:329).

30) Maxilla, antorbital fossa, contact with nasal, lateral view: reaches (0) or does not reach (1) the nasal suture (Carr, 1999; Holtz, 2001:49; var. Currie et al., 2003:34, 36, 37; Carr and Williamson, 2010:19; Brusatte et al., 2010:21; Brusatte and Carr, 2016:21).

31) Maxilla, interfenestral strut, rostrocaudal length, lateral view: greater than (0) or less than (1) 50% long axis of maxillary fenestra (Carr and Williamson, 2010:21; Brusatte et al., 2010:22; Brusatte and Carr, 2016:22).

32) Maxilla, interfenestral strut, base, perforating fenestra, presence, lateral view: absent (0), present (1) (Carr and Williamson, 2004; Loewen et al., 2013:27; Brusatte and Carr, 2016:328).

33) NEW Maxilla, antorbital fossa above the interfenestral strut, deep fossa or perforation, presence, lateral view: absent (0), present (1) (Carr and Williamson, 2004).

34) Maxilla, main body, dorsoventral height at midlength of internal antorbital fenestra, lateral view: less than (0) 16%, between 16-22% (1), or greater than (2) 22% depth of skull (Carr and Williamson, 2010:23; Brusatte et al., 2010:23; var. Loewen et al., 2013:31; Brusatte and Carr, 2016:23). ORDERED.

35) Maxilla, antorbital fossa, trend of dorsoventral height along horizontal ramus below the internal antorbital fenestra, lateral view: uniform (0), decreases as it extends caudally (1) (Currie et al., 2003:28; Carr and Williamson 2010:24; Brusatte et al., 2010:24; Brusatte and Carr, 2016:24).

36) Maxilla, subcutaneous flange bordering the antorbital fossa laterally on the caudal end of the main body, resulting in a channel formed between the flange (=dorsally extended lateral lamina) and the fossa, lateral view: absent (0), present (1) (Carr and Williamson 2010:25; Brusatte et al., 2010:25; Brusatte and Carr, 2016:25).

37) Maxilla, dorsolateral process, coverage by antorbital fossa, lateral view: process absent (0), process covered by subcutaneous surface only (1), ventral half of process covered by antorbital fossa (2), process completely covered by fossa (3) (var. Currie et al., 2003:34; Carr and Williamson 2010:27; Brusatte et al., 2010:26; Brusatte and Carr, 2016:26). ORDERED.

38) Maxilla, antorbital fossa, rostral limit, lateral view: does not extend ahead of the boundary of the external antorbital fenestra (0), extends ahead of the boundary as a narrow region of smooth surface texture ahead of the rostral margin of antorbital fossa onto the subcutaneous surface (1) (Carr and Williamson 2010:29; Brusatte et al., 2010:27; Brusatte and Carr, 2016:27).

39) Maxilla, ventral margin of the rostral region of the bone, profile: straight (0), convex (1) (Holtz, 2001:44; Sereno et al. 2009:14; Carr and Williamson 2010:29; Brusatte et al., 2010:28; Loewen et al., 2013:43; Brusatte and Carr, 2016:28).

40) Maxilla, joint surface for the palatine, dorsoventral height, medial view: shallow, does not conceal the tooth root bulges from view (0); tall, conceals tooth root bulges from view (1) (Carr, 1999; Currie et al., 2003:32; Carr and Williamson 2010:30; Brusatte et al., 2010:29; Brusatte and Carr, 2016:29).

41) Maxilla, rostral ramus, concave step in rostral margin of maxilla, presence, lateral view: absent (0), present (1) (Carr and Williamson, 2010:23; Brusatte et al., 2010:30; Loewen et al., 2013:29; Brusatte and Carr, 2016:30).

42) Maxilla, form of contact with nasal in subadult to adult specimens, lateral view: smooth (0), weakly scalloped (1), deeply scalloped with interlocking transverse ridges on both bones (2). (Currie et al., 2003:60; Sereno et al. 2009:15; Brusatte et al., 2010:31; Loewen et al., 2013:39; Brusatte and Carr, 2016:31). ORDERED.

43) Maxilla, subcutaneous surface texture, form, lateral view A: random foramina and shallow grooves and ridges (0); deep, prominent, dorsoventrally trending grooves and ridges (1); extremely coarse and deep sulci rostral to the antorbital fossa (2) (Carr and Williamson, 2010:28; Brusatte et al., 2010:32; Loewen et al., 2013:40; Brusatte and Carr, 2016:32).

44) NEW Maxilla, subcutaneous surface texture, form, lateral view B: deep fossae and prominent ridges absent (0), deep fossae and prominent ridges present (1).

45) Maxilla, swollen rim separating antorbital fossa and subcutaneous surface, presence, lateral view: present (0); absent (1) (Brusatte et al., 2010:33; Brusatte and Carr, 2016:33).

46) Maxilla, size of ascending ramus, rostrocaudal chord directly above maxillary fenestra compared to dorsoventral depth of maxilla below rostral edge of the internal antorbital fenestra, lateral view: greater than 1.75 times (ascending ramus large) (0), less than 1.60 times (ascending ramus small) (1) (Brusatte et al., 2010:34; Brusatte and Carr, 2016:34).

47) NEW Maxilla, maxillary flange (convex emargination above the antorbital fossa that laps onto the nasal), presence, lateral view: absent (0), present (1).

48) Maxilla, caudal region of the main body (portion including the final 3-5 teeth and rostral to the jugal process), shape, lateral view: maintains a relatively constant dorsoventral depth (0); tapers in depth caudally (1) (Brusatte et al., 2010:35; Brusatte and Carr, 2016:35).

49) Maxilla, primary row of neurovascular foramina, form, lateral view: continues as a row caudally (0); transitions into a sharp groove, paralleling the antorbital fossa rim caudally (1) (Brusatte et al., 2010:36; Loewen et al., 2013:41; Brusatte and Carr, 2016:36).

50) Maxilla, antorbital fossa, extent on main body under midpoint of the internal antorbital fenestra, lateral view: covers more than 65% (0), between 50-60% (1), or less than 45% (2) of the depth of the bone (Brusatte et al., 2010:37; Loewen et al., 2013:42; Brusatte and Carr, 2016:37). ORDERED.

51) Maxilla, palatal shelf, shape, medial view: straight along its rostrocaudal length (0); sigmoid, such that the ventral margin is concave and convex in parts (1) (Loewen et al., 2016:45; Brusatte and Carr, 2016:330).

52) Maxilla, palatal shelf, dental pits, presence, medial view: absent (0), present (1) (Loewen et al., 2013:46; Brusatte and Carr, 2016:331). We changed the score for *Appalachiosaurus* from ? to 1, following the description of Carr et al. (2005).

NASAL (12 characters)

53) Nasals, shape of dorsal surface, dorsal view: flat or slightly convex (0); convex (vaulted) rostrally, above and immediately caudal to the external naris (1); vaulted along most of their length (2) (Currie et al., 2003:41; Sereno et al., 2009:5; Carr and Williamson, 2010:36; Brusatte et al., 2010:38; Loewen et al., 2013:54; Brusatte and Carr, 2016:38). ORDERED.

54) Nasal, premaxillary processes, extent of their apposition to each other on the midline, dorsal view: apposed for nearly their entire length, may abruptly separate from each other at their tips (0); not apposed for most of their length, and therefore do not abruptly separate at their tips (1) (Brusatte and Carr, 2016:320).

55) Nasals, midline crest on dorsal surface, lateral and dorsal views: absent (0); present (1) (Carr and Williamson, 2010:48-52; Brusatte et al., 2010:39; Loewen et al., 2013:49; Brusatte and Carr, 2016:39).

56) Nasal, external texture of mid section of bone, condition, lateral and dorsal views: smooth to slightly rugose (0), pronounced rugosities and associated vascular foramina present (1) (Bakker et al., 1988; Holtz, 2001:46; Sereno et al. 2009:7; Carr and Williamson, 2010:47; Brusatte et al., 2010:40; Loewen et al., 2013: 52, 53; Brusatte and Carr, 2016:40). We changed the score of ? to 1 for *Teratophoneus*; the texture of the nasal splits caudally and extends onto the dorsum of the lacrimal, which can then be used to infer the texture of the nasals.

57) Nasal, series of pronounced, discrete rugosities on dorsal surface of middle portion of bone, posterior to the external naris, presence, lateral and dorsal views: absent (0); present (1) (Bakker et al., 1988; var. Holtz, 2001:90; Lu et al., 2014:315; Brusatte and Carr, 2016:313).

58) Nasal, shape, dorsal view: expands in width caudally (0); relatively constant width along the length of the bone, due to subparallel lateral sides (1); tapers in width caudally (2) (Russell, 1970; Currie et al., 2003:39; Sereno et al., 2009:6; Brusatte et al., 2010:41;

Loewen et al., 2013:47; Brusatte and Carr, 2016:41).

59) Nasal, frontal process, mediolateral width, dorsal view: unconstricted (0); constricted, less than ½ width of widest point of nasal (1) (Russell, 1970; Holtz, 2001:47; Currie et al., 2003:39; Carr and Williamson, 2010:40; Brusatte et al., 2010:42; Loewen et al., 2013:57; Brusatte and Carr, 2016:42).

60) Nasal, internal pneumatic recess, presence, lateral view: present (0), absent (1) (Carr and Williamson, 2010:38; Brusatte et al., 2010:43; Loewen et al., 2013:50; Brusatte and Carr, 2016:43).

61) Nasal, caudolateral process that underlaps the rostrodorsal process of the lacrimal, lateral view: absent (0); present (1) (Currie et al., 2003:38; Sereno et al., 2009:4; Carr and Williamson, 2010:39; Brusatte et al., 2010:44; Loewen et al., 2013:59; Brusatte and Carr, 2016:44).

62) Nasal, narial fossa, extent on premaxillary process, lateral and dorsal views: limited to ventral margin of process (0); covers entire process, and thus meets opposite fossa on dorsal midline (1) (Sereno et al., 2009:2; Brusatte et al., 2010:45; Brusatte and Carr, 2016:45).

63) Nasal, medial processes of frontal articulation, shape, dorsal view: processes absent (0), lanceolate (1), tapered (2) (Carr and Williamson, 2010:43; Brusatte et al., 2010:46; Brusatte and Carr, 2016:46).

64) Nasal, thin, low, and laterally projecting crest at the corner where lateral and dorsal surfaces meet, presence, lateral and dorsal views: absent (0), present (1) (Brusatte et al., 2010:47; Loewen et al., 2013:51; Brusatte and Carr, 2016:47).

LACRIMAL (25 characters)

65) Lacrimal, shape, lateral view: inverted L (0), 7 shaped (1) (Holtz, 2001:54; Currie et al., 2003:23; Carr and Williamson, 2010:54; Brusatte et al., 2010:48; Loewen et al., 2013:61; Brusatte and Carr, 2016:48).

66) Lacrimal, cornual process on dorsal surface of bone, presence: absent (0), present (1) (var. Currie et al., 2003:24; Carr and Williamson, 2010:55; Brusatte et al., 2010:49; Loewen et al., 2013:63; Brusatte and Carr, 2016:316).

67) Lacrimal, cornual process on dorsal surface, form, lateral view: absent (0); present as a broad, shallow, dorsally convex, laterally overhanging swelling across most of the length of the rostral ramus (1); present as a discrete conical projection (2); small, conical, smooth projection that rises 2-3 millimeters from skull roof (3) (Bakker et al., 1988; var. Holtz, 2001:51, 52; var. Currie et al., 2003:24; Sereno et al., 2009:18; Carr and Williamson, 2010:56; Brusatte et al., 2010:49; Loewen et al., 2013:66; Brusatte and Carr, 2016:49).

68) Lacrimal, cornual process, distinct apex, presence, lateral view: smoothly rounded (0), discrete apex present (1) (var. Holtz, 2001:51; var. Currie et al., 2003:24; Carr and Williamson, 2010:56; Brusatte et al., 2010:50; Brusatte and Carr, 2016:50).

69) Lacrimal, cornual process, height, lateral view: tall, greater than 60% height of antorbital fossa below it (0); short, less than 60% of the height of the fossa below it (1) (var. Loewen et al., 2013:64; Brusatte and Carr, 2016:359).

70) Lacrimal, cornual process, position of apex, lateral view: dorsal to ventral ramus (0); rostral to ventral ramus (1) (Holtz, 2001:53; Sereno et al., 2009:19; Carr and Williamson, 2010:58; Brusatte et al., 2010:51; Loewen et al., 2013:67; Brusatte and Carr, 2016:51).

71) Lacrimal, cornual process, form, rostral view: not inflated (1); inflated, such that it appears swollen (1) (Brusatte et al., 2010:49; var. Loewen et al., 2013:66; var. Brusatte and Carr, 2016:49).

72) Lacrimal, accessory caudally extending cornual process on the lateral surface between the cornual process and the supraorbital ramus, presence, lateral view: absent (0), present (1) (Carr and Williamson, 2010:70; Brusatte and Carr, 2016:360).

73) Lacrimal, extent of pneumaticity: absent in that the lacrimal pneumatic recess is not seen (0); limited, partially hollows bone in the region where the anterior and ventral rami meet (1); extensive, completely hollows bone (2) (Sereno et al., 2009:16; Brusatte et al., 2010:52; Loewen et al., 2013:70; Brusatte and Carr, 2016:52). ORDERED.

74. Lacrimal, rostral ramus, length relative to the ventral ramus: shorter than the ventral ramus (0), longer than the ventral ramus (1) (Loewen et al., 2013:62).

75) Lacrimal, rostral ramus, inflation, lateral and dorsal views: not inflated (0); inflated by pneumatic recess (1) (Holtz, 2001:50; Sereno et al., 2009:17; Carr and Williamson, 2010:63; Brusatte et al., 2010:53; Brusatte and Carr, 2016:53).

76) Lacrimal, lacrimal pneumatic recess, size, lateral view: small, rostral end located approximately at the same level as the rostral end of the ventral ramus (0); large, rostral end located far ahead of the ventral ramus (1) (Carr and Williamson, 2010:50; Brusatte et al., 54; Loewen et al., 2013:75; Brusatte and Carr, 2016:54).

77) Lacrimal, transition between antorbital fossa and the subcutaneous surface of the ventral ramus, form, lateral view: surfaces are continuous with each other (0); fossa is deeply inset, forming a ridge along the subcutaneous surface (1) (Carr and Williamson 2010:61; Brusatte et al., 2010:55; Brusatte and Carr, 2016:55).

78) Lacrimal, accessory external openings for lacrimal recess on the rostral ramus, lateral view: absent (0); present and proximally located (1); present and distally located (2) (var. Holtz, 2001:99; Carr and Williamson, 2010:66; Brusatte et al., 2010:56; Loewen et al., 2013:71, 72; Brusatte and Carr, 2016:56). Note: we retain the multistate coding of Brusatte et al. (2010) that was changed to binary in Brusatte and Carr (2016).

79) Lacrimal, pneumatic recess opening internally onto medial surface of bone as a discrete

pneumatic fenestra, presence, medial view: absent (0), present (1). (Carr and Williamson 2010:67; Brusatte et al., 2010:57; Loewen et al., 2013:73; Brusatte and Carr, 2016:57).

80) Lacrimal, pneumatic recess opening internally onto medial surface of bone, position, medial view: faces rostrally (0); situated on the medial surface (1) (Carr and Williamson 2010:68).

81) Lacrimal, rostrodorsal process of rostral ramus for articulation with maxilla and nasal, presence and size, lateral view: absent or small (0), present and long (1) (var. Currie et al., 2003:59; Carr and Williamson, 2010:64; Brusatte et al., 2010:58; Brusatte and Carr, 2016:58).

82) Lacrimal, supraorbital process, size and shape, lateral view: absent or very short (0); present, long and gracile, dorsoventrally shallow (1); present, long and robust, dorsoventrally tall (2) (Loewen et al., 2013:68; Brusatte and Carr, 2016:333). ORDERED.

83) Lacrimal, ventral ramus, subocular process, presence, lateral view: absent (0), present and subtle (1), present and not subtle (2) (Carr and Williamson, 2010:69; Loewen et al., 2013:81; Brusatte and Carr, 2016:334). ORDERED.

84) Lacrimal, ventral ramus, rostroventral ala, dorsoventral height, lateral view: greater (0) or less than (1) half of the dorsoventral depth of the ramus (Carr and Williamson, 2010:71; Brusatte et al. 2010:59; Brusatte and Carr, 2016:59).

85) Lacrimal, orbitonasal ridge on medial surface, position: rostral (0) or adjacent to or contacting (1) caudal margin of ventral ramus (Carr and Williamson, 2010:68; Brusatte et al., 2010:60; Loewen et al., 2013:79; Brusatte and Carr, 2016:60).

86) Joint surface for the frontal, form, medial view: dorsoventrally flat (0), conical (1) (Brusatte et al., 2010:61; Brusatte and Carr, 2016:61).

87) Lacrimal, supraorbital process for articulation with frontal, inflation, presence, medial view: absent (0), present (1) (Brusatte et al., 2010:62; Loewen et al., 2013:74; Brusatte and Carr, 2016:62).

88) Lacrimal, rostroventral ala, length, lateral view: covers greater (0) or less than (1) 60% of rostrocaudal length along the contact with the jugal (Brusatte et al., 2010:63; Loewen et al., 2013:80; Brusatte and Carr, 2016:63).

89) Lacrimal, maxillary process of rostral ramus, exposure, lateral view: both dorsal and ventral margins visible (0), dorsal margin concealed by subcutaneous surface above antorbital fossa and only ventral margin visible (1) (Carr and Williamson, 2010:65; Brusatte et al., 2010:64; Brusatte and Carr, 2016:64).

Jugal (20 characters)

90) Jugal, maxillary ramus, depth: shallow, not expanded relative to suborbital portion of bone (0), deep, expanded relative to suborbital portion of bone (1) (Carr, 1999; Currie et al., 2003:16; Carr and Williamson, 2010:72; Brusatte et al., 2010:65; Loewen et al., 2013:135;

Brusatte and Carr, 2016:65).

91) Jugal, antorbital fossa, extent on maxillary ramus, lateral view: edge of fossa undercut and continues caudodorsal to jugal recess (0), fossa edge does not extend past the jugal recess (1) (Russell, 1970; Holtz, 2001:55; Carr and Williamson, 2010:74; Brusatte et al., 2010:66; Loewen et al., 2013:133; Brusatte and Carr, 2016:66).

92) Jugal, pneumatic recess, location relative to ventral ramus of lacrimal, lateral view: ventral (0), rostral (1) (Carr and Williamson, 2010:75; Brusatte et al., 2010:67; var. Loewen et al., 2013:134; Brusatte and Carr, 2016:67).

93) Jugal, pneumatic recess, orientation of long axis, lateral view: approximately horizontal (0), inclined at approximately 45 degrees relative to the ventral skull margin (1) (Currie et al., 2003:19; Carr and Williamson, 2010:77; Brusatte et al., 2010:68; Loewen et al., 2013:132; Brusatte and Carr, 2016:68).

94) Jugal, secondary fossa, presence, lateral view: absent (0), present (1) (Carr and Williamson, 2010:78).

95) Jugal, secondary fossa for pneumatic recess, dorsoventral position relative to recess, lateral view: ventral (0), dorsal (1) (Carr and Williamson, 2010:79; Brusatte et al., 2010:69; Brusatte and Carr, 2016:69).

96) Jugal, suture with lacrimal, angle of the posterior half of the contact, lateral view: low (0); steep (1) (Carr and Williamson, 2010:80; Brusatte et al., 2010:70; Loewen et al., 2013:136; Brusatte and Carr, 2016:70).

97) Jugal, fossa on lateral surface of postorbital ramus, depth inset into bone, lateral view: shallow (0); deep (1) (Sereno et al., 2009:24; Carr and Williamson, 2010:81; Brusatte et al., 2010:71; Brusatte and Carr, 2016:71).

98) Jugal, articulation with postorbital, form of ventral extremity of suture, lateral view: tapering scarf joint (0); shallow interlocking notch for postorbital (1); deep interlocking notch for postorbital, defined by a flange rostral to the notch on the jugal (2) (Currie et al., 2003:21; Sereno et al., 2009:26; Carr and Williamson, 2010:82; Brusatte et al., 2010:72; Loewen et al., 2013:137; Brusatte and Carr, 2016:72). ORDERED.

99) Jugal, articulation with postorbital, extent of scarf joint on lateral surface of postorbital ramus, lateral view: limited, occupies less than 50% of rostrocaudal length of the process (0); extensive, occupies approximately 50-75% of the rostrocaudal length of the process (1) (Brusatte et al., 2010:73; Brusatte and Carr, 2016:73).

100) Jugal, articulation with postorbital, braced by a pronounced ridge on the lateral surface of the postorbital ramus that borders the postorbital posteriorly, lateral view: absent (0), present (1) (Brusatte et al., 2010:74; Brusatte and Carr, 2016:74).

101) Jugal, postorbital ramus, orientation relative to ventral margin of jugal, lateral view: approximately perpendicular (0); caudodorsal (obtuse angle between the long axis of the process and the ventral margin) (1) (Brusatte et al., 2010:75; Loewen et al., 2013:140; Brusatte and Carr, 2016:75).

102) Jugal, postorbital process, basal rostrocaudal length relative to suborbital height, lateral view: rostrocaudally short, less than 100% suborbital height (0); rostrocaudally long, greater than 100% suborbital height (1) (var. Currie et al., 2003:20; Loewen et al., 2013:138; Brusatte and Carr, 2016:342).

103) Jugal, cornual process, presence, lateral view: absent (0); present (1); present and distinctive (mediolaterally wide and heavily rugose (2) (Carr, 1999; Currie et al., 2003:18; Carr and Williamson, 2010:83; Brusatte et al., 2010:76; Loewen et al., 2013:145; Brusatte and Carr, 2016:76). ORDERED.

104) Jugal, ornamentation on the lateral surface, form, lateral view: absent (0), rugose thickening present (1), pronounced laterally directed horn or boss (Loewen et al., 2013:143; Brusatte and Carr, 2016:343). ORDERED.

105) Jugal, dorsal quadratojugal process, orientation, lateral view: horizontal (0), caudodorsal (1) (Carr and Williamson, 2010:89; Brusatte et al., 2010:77; Loewen et al., 2013:144; Brusatte and Carr, 2016:77).

106) Jugal, ventral quadratojugal process, slope of joint surface, angle, lateral view: approximately rostrocaudally oriented, angled less than 45 degrees from horizontal (0); angled rostrodorsally at greater than 45 degrees from horizontal (1) (Carr and Williamson, 2010:88; Brusatte et al., 2010:78; Brusatte and Carr, 2016:78).

107) Jugal, margin of the orbital fenestra, shape, lateral view: weakly concave, approximately level with lacrimojugal suture (0); U-shaped, extends ventral to lacrimal-jugal suture (1) (var. Currie et al., 2003:22; Sereno et al., 2009: 26; Brusatte et al., 2010:79; Loewen et al., 2013:141; Brusatte and Carr, 2016:79).

108) Jugal, margin of the orbital fenestra in juveniles, form, lateral view: straight (0), concave (1) (Brusatte and Carr, 2016:361).

109) Jugal, raised rim on the lateral surface, paralleling the ventral margin of the bone and rostrally confluent with the antorbital fossa rim of the maxilla, presence, lateral view: absent (0); present (1) (Brusatte et al., 2010:80; Loewen et al., 2013:145; Brusatte and Carr, 2016:80).

Postorbital (11 characters)

110) Postorbital, cornual process, presence and form, lateral view: absent (0); limited to rugose rim at caudodorsal corner of orbit (1); present as a rugose, convex boss (2) (Holtz, 2001:56; Sereno et al., 2009:29; Brusatte et al., 2010:81; Loewen et al., 2013:110). ORDERED.

111) Postorbital, cornual process, dorsoventral position, lateral view: separated from dorsal

margin of postorbital by a smooth, convex region (0); approaches or extends past dorsal margin of bone (1) (Carr and Williamson, 2010:92: Brusatte et al., 2010:82; Brusatte and Carr, 2016:82).

112) Postorbital, cornual process, position relative to orbital fenestra, lateral view: located at orbital margin (0), located caudodorsal to orbital margin (1) (var. Currie et al., 2003:48; Carr and Williamson, 2010:93; Brusatte et al., 2010:83; Loewen et al., 2013:111; Brusatte and Carr, 2016:83).

113) Postorbital, cornual process, position relative to laterotemporal fenestra, lateral view: does not approach fenestra (0), closely approaches fenestra (1) (Carr and Williamson, 2010:91).

114) Postorbital, squamosal ramus, form of caudodorsal margin, lateral view: uninterrupted convex arc or marked by very shallow concave notch (0), emarginated by squamosal (discrete concave notch within the margin) (1) (Carr and Williamson, 2010:97; Brusatte et al., 2010:84; Loewen et al., 2013:113; Brusatte and Carr, 2016:84).

115) Postorbital, squamosal ramus, extent relative to the laterotemporal fenestra, lateral view: reaches or extends caudal to (0) or terminates rostral to (1) caudal margin of laterotemporal fenestra (Sereno et al. 2009:28; Carr and Williamson, 2010:98; Brusatte et al., 2010:85; Loewen et al., 2013:114; Brusatte and Carr, 2016:85).

116) Postorbital, subocular process that extends into the orbit, presence and size, lateral view: absent, ventral ramus tapers (0); present and large in adults, small and unpronounced (ventral ramus tongue-shaped) in subadults (1); present and large in sub-adults and adults (2) (Holtz, 2001:57; Currie et al., 2003:47; Sereno et al., 2009:30; Carr and Williamson, 2010:99; Brusatte et al., 2010:86; Loewen et al., 2013:109; Brusatte and Carr, 2016:86). ORDERED.

117) Postorbital, subocular process, dorsoventral position, lateral view: at ventral end of ventral process (0); flangelike, separated from ventral tip of ventral process by a notch (1) (Carr and Williamson 2010:100; Brusatte et al., 2010:87; Loewen et al., 2013:109; Brusatte and Carr, 2016:87).

118) Postorbital, rostral ramus, form, lateral view: short and stout, long axis is approximately half the length of the ventral ramus and the thickness at the base is approximately the same as the thickness of the midpoint of the ventral ramus (0); long and slender, long axis is greater than 60% of the length of the ventral ramus and the thickness at the base is approximately half that of the midpoint of the ventral ramus (1) (Li et al., 2010:252; Brusatte et al., 2010:88; Loewen et al., 2013:104; Brusatte and Carr, 2016:88).

119) Postorbital, rostral ramus, shape, lateral view: dorsoventrally tall, rostral tip is greater than half the thickness of the ventral process (0); dorsoventrally shallow, rostral tip is less than 30% of the rostrocaudal thickness of the ventral process at midlength (1) (Loewen et al., 2013:105; Brusatte and Carr, 2016:338).

120) Postorbital, jugal ramus, rostrocaudal length at midheight, lateral view: approximately the same width as (0) or substantially wider than (1) ventral ramus of the lacrimal (Li et al., 2010:

268; Brusatte et al., 2010:89; Loewen et al., 2013:107; Brusatte and Carr, 2016:89).

PALPEBRAL BONES (1 character)
121) Palpebral bones, presence, lateral and dorsal views: absent (0); present, one caps the caudal end of the lacrimal and a second extends from the lacrimal onto the lateral surface of the postorbital above the orbital fenestra (1) (Holtz, 2001:58; Carr and Williamson, 2010:102; Loewen et al., 2013:116; Brusatte and Carr, 2016:339).

SQUAMOSAL (10 characters)
122) Squamosal, lateral ridge delimiting dorsotemporal fossa, form, dorsal view: ridge unpronounced or undivided (0), divided into a pair of ridges (1) (Carr and Williamson 2010:104; Brusatte et al., 2010:90; Brusatte and Carr, 2016:90).

123) Squamosal, dorsotemporal fossa, surface morphology, dorsal view: flat or concave (0), convex (1) (Carr and Williamson, 2010:105; Brusatte et al., 2010:91; Loewen et al., 2013:121; Brusatte and Carr, 2016:91).

124) Squamosal, quadratojugal process, long axis, orientation, lateral view: dorsoventral or slightly oblique (0), rostrocaudal (1) (Carr and Williamson 2010:109; Brusatte et al., 2010:92; Loewen et al., 2013:122; Brusatte and Carr, 2016:92).

125) Squamosal, quadratojugal process, morphology of anterior tip in those taxa with a horizontal process, lateral view: tapered point (0), squared off (1). (Carr and Williamson, 2010:107, 108; Brusatte et al., 2010:93; Loewen et al., 2013:124; Brusatte and Carr, 2016:93).

126) Squamosal, quadratojugal process, flange that is covered laterally by the quadratojugal, dorsoventral depth of entire process compared to portion of process that is exposed when in articulation with quadratojugal, lateral view: flange absent (0), thinner (1), substantially thicker (2) (Carr and Williamson, 2010:110; Brusatte et al., 2010:94; Loewen et al., 2013:125; Brusatte and Carr, 2016:94). ORDERED.

127) Squamosal, caudal process, inflation by squamosal recess, presence, lateral and caudal views: absent (0), present (1) (Carr and Williamson, 2010:112; Brusatte et al., 2010:95; Brusatte and Carr, 2016:95).

128) Squamosal, caudal process, length of the long axis compared to the long axis of the quadratojugal process, length, lateral view: long, approximately 1/3-1/2 (0); short, approximately 1/6 (1) (Carr and Williamson 2010:113; Brusatte et al., 2010:96; Loewen et al., 2013:129; Brusatte and Carr, 2016:96).

129) Squamosal, postorbital process, flange that extends dorsal to the squamosal process of the postorbital, lateral view: absent (0), present (1) (Sereno et al., 2009:32; Brusatte et al., 2010:97; Brusatte and Carr, 2016:97).

130) Squamosal, postorbital process, rostral tip, position, lateral view: at or ahead of the rostral margin of the laterotemporal fenestra (0), caudal to the rostral margin of the fenestra (1) (Carr

and Williamson, 2010:96; Loewen et al., 2013:117; Brusatte and Carr, 2016:340).

131) Squamosal, pneumaticity, presence, ventral view: pneumatic recess absent, but instead a fossa is seen in the ceiling of the bone (0); recess is present, in which the edges of the recess are undercut and lead into a pneumatic space that hollows out the bone (1) (Holtz, 2001:4; Currie et al., 2003:68; Carr and Williamson, 2010:111; Loewen et al., 2013:126; Brusatte and Carr, 2016:341).

QUADRATOJUGAL (9 characters)
132) Quadratojugal and squamosal, constriction of laterotemporal fenestra: absent, rostral margin of both bones are approximately vertical (0); present, convex kink along the suture between the two bones that projects into the fenestra, constricting it to approximately one half of its maximum rostrocaudal length (1); present, dorsal region of quadratojugal moderately expanded rostrocaudally relative to the remainder of the bone, constricting fenestra to approximately one half of its maximum rostrocaudal length (2); present, dorsal region of quadratojugal expanded rostrocaudally by at least twice the minimum rostrocaudal dimension of the bone, forming a flange that meets the ventral ramus of the squamosal to nearly divide the fenestra (3) (Bakker et al., 1988; Holtz, 2001:4; var. Currie et al., 2003:56, 69; var. Sereno et al., 2009:35; Carr and Williamson, 2010:114; Brusatte et al., 2010:98; Loewen et al., 2013:152; Brusatte and Carr, 2016:98). ORDERED.

133) Quadratojugal, dorsal process, ridge along rostral margin of lateral surface, presence and form, lateral view: absent (0); present, subtle and fades in strength dorsally (1); present, robust and extends to the dorsal margin of the bone (2) (Carr and Williamson, 2010:115; Brusatte et al., 2010:99; Loewen et al., 20013:152; Brusatte and Carr, 2016:99). ORDERED.

134) Quadratojugal, jugal articulation, form, lateral and dorsal views: dorsal quadratojugal process of jugal does not (0) or does (1) approach the base of the quadratojugal (caudoventral corner of the laterotemporal fenestra) (Carr and Williamson, 2010:118; Brusatte et al., 2010:100; Loewen et al., 2013:146; Brusatte and Carr, 2016:100).

135) Quadratojugal, rostral process for articulation with jugal, form of rostral region, lateral view: tapered (0); rounded (1); squared off or double pronged (2) (Currie et al., 2003:57; Sereno et al., 2009:34; Carr and Williamson, 2010:119; Brusatte et al., 2010:101; Loewen et al., 2013:144; Brusatte and Carr, 2016:101).

136) Quadratojugal, rostral process, extent, lateral view: terminates caudal (0) or level with or rostral (1) to rostral margin of laterotemporal fenestra (Sereno et al., 2009:36; Carr and Williamson, 2010:86; Brusatte et al., 2010:102; Brusatte and Carr, 102).

137) Quadratojugal, curvature of bone, caudal view: mediolaterally narrow and flat (0); caudal region flexed so that it curves caudally, thus delimiting the lateral edge of a deep pocket that borders the quadrate foramen laterally (1) (Li et al., 2010:281; Brusatte et al. 2010:103; Brusatte and Carr, 2016:103).

138) Quadratojugal, ventral quadrate process, presence, lateral and caudal views: absent, the bone is an inverse L-shape (0); present, the bone is the shape of an inverted T (1) (Carpenter et al., 2005; Carr and Williamson, 2010:120; Brusatte et al., 2010:104; Loewen et al., 2016:153; Brusatte and Carr, 2016:104).

139) Quadratojugal, ventral quadrate process, length and orientation, lateral and caudal views: short, oriented mostly laterally (0); long, wraps onto the caudal surface of the quadrate condyles (1) (Brusatte et al., 2010:104; Loewen et al., 2013:154; Brusatte and Carr, 2016:104).

140) NEW Quadratojugal, shaft, pneumatic foramen, presence, lateral view: absent (0), present at some point during ontogeny (1).

QUADRATE (8 characters)

141) Quadrate, quadrate foramen, size, caudal view: small, long axis approximately 10% of the dorsoventral height of the quadrate shaft (0); large, long axis greater than 20% of the dorsoventral height of the quadrate shaft (1) (Currie et al., 2003:53; Carr and Williamson, 2010:123; Brusatte et al., 2010:105; Loewen et al., 2013:155; Brusatte and Carr, 2016:105).

142) Quadrate, quadrate foramen, position of ventral edge of the foramen from the ventral edge of the quadrate: more than twice the dorsoventral depth of the quadrate condyles in caudal view (0), twice the depth of the condyles or less (1) (Loewen et al., 2013:158; Brusatte and Carr, 2016:345).

143) Quadrate, pneumaticity, presence, rostral view: absent (0); present, deep recess on the rostral surface where the pterygoid wing and condyles meet (1) (Molnar, 1974; Currie et al., 2003:55; Carr and Williamson, 2010:124; Brusatte et al., 2010:106; Loewen et al., 2013:159; Brusatte and Carr, 2016:106).

144) Quadrate, form of pneumatization, rostral and caudal views: large single pneumatic foramen on caudal or caudomedial surface of shaft present (0), absent (1) (Gold et al., 2013; Brusatte and Carr, 2016:318).

145) Quadrate, mandibular condyles, position relative to occipital condyle when skull is in articulation, lateral view: approximately aligned (0); completely caudal (1) (Sereno et al., 2009:42; Carr and Williamson, 2010:125; Brusatte et al., 2010:107; Loewen et al., 2013:161; Brusatte and Carr, 2016:107).

146) Quadrate, quadratojugal articulation, extent on lateral surface of lateral condyle, lateral and caudal views: limited, occupies only part of the surface (0); extensive, covers entire lateral surface and extends dorsally to partially enclose quadrate foramen laterally (1) (Brusatte et al., 2010:108; Brusatte and Carr, 2016:108).

147) Quadrate, articular surface for quadratojugal on quadrate lateral condyle, orientation of medial margin where quadratojugal wraps around quadrate, caudal view: vertical or dorsomedial (0); dorsolateral (1) (Carr and Williamson, 2010:122; Brusatte et al., 2010: 109; Brusatte and Carr, 2016:109).

148) Quadrate, orbital process, medial surface, presence and depth, medial view: absent (0); shallowly inset (1); deeply excavated (2) (Loewen et al., 2013:160; Brusatte and Carr, 2016:346). ORDERED.

PREFRONTAL (2 characters)
~~148) Prefrontal, contacts nasal, dorsal view: yes (0); no, excluded by frontal-lacrimal contact (1) (Brusatte et al., 2010).~~ Note: this character was discarded because the derived state is only coded for *Guanlong*; an autapomorphy is uninformative regarding ingroup relationships.

149) Prefrontal, exposure, dorsal view: widely exposed, forms much of orbital rim and usually separates or nearly separates frontal and lacrimal (0); reduced, not exposed along the orbital rim and allows for wide contact between frontal and lacrimal (1) (var. Holtz, 2001:5; Currie et al., 2003:50; Sereno et al., 2009:20; var. Carr and Williamson, 2010:128; Brusatte et al., 2010:111; Brusatte and Carr, 2016:111).

150) Prefrontal, ventral process, extent, lateral view: large, extends more than halfway down the ventral ramus of the lacrimal to make an extensive contribution to the preorbital bar (0); reduced or absent, ventral process is a thin flange that is continuous with the crista cranii of the frontal, and does not extend more than approximately 1/4 of the length of the preorbital bar (1) (Carr and Williamson, 2010:130; Brusatte et al., 2010:112; Loewen et al., 2013:84: Brusatte and Carr, 2016:112).

FRONTAL (11 characters)
151) Frontal, shape, dorsal view: triangular (0); caudal end expanded into a rectangular shape, with a small rostral triangle (1) (Holtz, 2001:61; Sereno et al. 2009:38; Brusatte et al., 2010:113; Brusatte and Carr, 2016:113).

152) Frontal, size of single frontal, ratio of rostrocaudal length of exposed portion on skull roof to mediolateral width at midpoint, dorsal view: greater than 2.5 (0), less than 2.0 (1) (Brusatte et al., 2010:114; Loewen et al., 2013:89; Brusatte and Carr, 2016:114).

153) Frontal, prefrontolacrimal process, rostrocaudal length, dorsal view: absent or short (0), long (1) (Brusatte and Carr, 2016:362).

154) Frontal, nasal process, ridge that extends rostrocaudally along the process, presence, dorsal view: absent (0), present (1) (Brusatte and Carr, 2016:363).

155) Frontal, dorsotemporal fossa, rostrocaudal length compared to overall length of exposed portion of frontal on skull roof, dorsal view: less than 30% (0), between 30-50% (1), between 50-60% (2), greater than 60% (3) (Bakker et al., 1988; var. Holtz, 2001:62; Carr and Williamson, 2010:134, 135; Brusatte et al., 2010:115; Loewen et al., 2013:94; Brusatte and Carr, 2016:115). ORDERED.

156) Frontal, dorsotemporal fossa, medial extension, dorsal view: fossa restricted to caudolateral corner of frontal (0); meets opposing fossa at the midline (1) (var. Holtz, 2001:62; var. Currie et

al., 2003:64; Sereno et al., 2009:39; Brusatte et al., 2010:116; Loewen et al., 2013:95; Brusatte and Carr, 2016:116).

157) Frontal, sagittal crest, form, dorsal and lateral views: absent or subtle, only discernable as a slight midline bulge (0); present and pronounced (dorsoventrally tall), single structure (1); present, dorsoventrally tall, and paired (2) (Carr and Williamson, 2010:137; Brusatte et al., 2010:117; Brusatte and Carr, 2016:117).

158) Frontal, sagittal crest, rostrocaudal length, dorsal view: absent or subtle and short, less than 15% length of the frontal (0); extensive, approximately 25% of the length of the frontal (1) (Carr and Williamson, 2010:139; Brusatte et al., 2010:118; Brusatte and Carr, 2016:118).

159) Frontal, postorbital suture, lateral and dorsal views: dorsoventrally shallow and undifferentiated (0); dorsoventrally shallow (approximately 6 times longer than deep) and differentiated into a vertical region rostrally and a horizontal region caudally (1); dorsoventrally deep, approximately twice as long as deep, and subtly differentiated into vertical and horizontal regions (2) (Currie et al., 2003:14; Sereno et al., 2009:37; Carr and Williamson, 2010:143; Brusatte et al., 2010:119; Loewen et al., 2013:96; Brusatte and Carr, 2016:119). ORDERED.

160) Frontal, contribution to orbital rim, dorsal and lateral views: extensive (0), present but limited to a small notch (1), excluded by postorbital-lacrimal contact in large specimens (2), excluded by postorbital-lacrimal articulation and novel palpebral ossification (3) (Sereno et al., 2009:21; Carr and Williamson, 2010:104; Brusatte et al., 2010:120; Loewen et al., 2013:90; Brusatte and Carr, 2016:120). ORDERED.

161) Frontal, mediolateral cross section ahead of the dorsotemporal fossa, dorsal view: flat (0); dorsally convex, in which the contralateral bones extend dorsomedially to the interfrontal suture (1); concave, where the bones extend ventromedially to the Interfrontal suture (2) (Loewen et al., 2013:97; Brusatte and Carr, 2016:336). ORDERED.

PARIETAL (10 characters)
162) Parietofrontal suture, form, dorsal view: extends mediolaterally (0); tab-like wedge from parietal extends anteriorly to overlie frontal on midline (1) (Currie et al., 2003:15; Carr and Williamson, 2010:145; Brusatte et al., 2010:121; Loewen et al., 2013:98; Brusatte and Carr, 2016:121).

163) Parietofrontal peak, presence, lateral view: absent (0), present (1) (Carr and Williamson, 2010:144; Loewen et al., 2013:100; Brusatte and Carr, 2016:335).

164) Parietal, sagittal crest, presence, dorsal view: absent (0), present (1) (Holtz, 2001:6; Carr and Williamson, 2010:146; Brusatte et al., 2010:122; Brusatte and Carr, 2016:122).

165) Parietal, sagittal crest, form, dorsal view: comprised of two parallel crests (0), comprised of a single midline crest (1) (Carr and Williamson, 2010:148; Brusatte et al., 2010:123; Loewen et al., 2013:99; Brusatte and Carr, 2016:123).

166) Parietal, skull table between dorsotemporal fossae, width, dorsal view: broad, 10-30% of the mediolateral width of the fossa (0); extremely reduced, sagittal crest or crests pinched between opposing fossae (1) (Sereno et al., 2009:40; Brusatte et al., 2010:124; Brusatte and Carr, 2016:124).

167) Parietal, sagittal crest, dorsoventral height, dorsal and lateral views: consistent across length of crest (0); peaked anteriorly at frontal-parietal suture (1) (Carr and Williamson, 2010:146; Brusatte et al., 2010:125; Brusatte and Carr, 2016:125).

168) Parietal, sagittal crest, ventral extent, lateral view: extends above the interorbital region (0), extends below the interorbital region (1) (Currie, 2003; var. Loewen et al., 2013:102; Brusatte and Carr, 2016:337).

169) Parietal, nuchal crest, dorsoventral depth, lateral view: as low as or lower (0) or extends higher (1) than the dorsal surface of the interorbital region (Bakker et al., 1988; var. Holtz, 2001:7; Currie et al., 2003:45; Sereno et al., 2009:41; Carr and Williamson, 2010:147; Brusatte et al., 2010:126; Loewen et al., 2013:103; Brusatte and Carr, 2016:126).

170) Parietal, nuchal crest, caudal surface in taxa where the crest extends higher than the dorsum of the interorbital region caudal view: thin but sharp midline ridge (0); mediolaterally wide triangle (apex point ventrally), which sometimes (but not always) has grooves on each side (1) (var. Loewen et al., 2013:167; Brusatte and Carr, 2016:348).

171) Parietals, fusion, dorsal view: unfused (0), fused on the midline in sub-adults and adults (1) (Brusatte et al., 2010:127; Brusatte and Carr, 2016:127).

VOMER (2 characters)
172) Vomer, shape of rostral end, dorsal and ventral views: lanceolate (lateral margins parallel-sided) (0), expanded into a diamond (1) (Holtz, 2001:11, 110; Currie et al., 2003:77; Sereno et al., 2009:45; Carr and Williamson, 2010:174; Brusatte et al., 2010:128; Loewen et al., 2013:207; Brusatte and Carr, 2016:128).

173) NEW Vomer, keel below stem, depth, lateral view: a low ridge (0), a deep keel-like flange (1).

ECTOPTERYGOID (5 characters)
174) Ectopterygoid, extent of internal recess, dorsal and ventral views: does not (0) or does (1) inflate the body of the bone and the pterygoid process (var. Holtz, 2001:69; var. Currie et al., 2003:9; Sereno et al., 2009:44; Carr and Williamson, 2010:176; Brusatte et al., 2010:129; Loewen et al., 2013:225; Brusatte and Carr, 2016:129).

175) Ectopterygoid, jugal process, external pneumatic foramina leading into jugal recess, caudal view: absent (0); present (1) (Carr and Williamson, 2010:178; Brusatte et al., 2010:130; Loewen et al., 2013:228; Brusatte and Carr, 2016:130).

176) Ectopterygoid, jugal process, inflation, presence, all views: absent (0), present (1) (Carr and

Williamson, 2010:179; Brusatte et al., 2010:131; Loewen et al., 2013:229; Brusatte and Carr, 2016:131).

177) Ectopterygoid, external opening of pneumatic recess, shape, ventral view: thin ovoid slot (0); large, round or triangular (1) (Holtz, 2001:68; Currie et al., 2003:10; Carr and Williamson 2010:181; Brusatte et al., 2010:132; Loewen et al., 2013:226; Brusatte and Carr, 2016:132).

178) Ectopterygoid, surface caudally adjacent to external opening of pneumatic recess, form, ventral view: flat, recess grades smoothly into the floor of the laterotemporal fenestra (=subtemporal fenestra) (0); ridge separates foramen from laterotemporal fenestra (=subtemporal fenestra) (1) (Carr and Williamson, 2010:183; Brusatte et al., 2010:133; Loewen et al., 2013:227; Brusatte and Carr, 2016:133).

PALATINE (11 characters)

179) Palatine, vomeropterygoid process, rostrocaudal length of dorsal margin ratio to length of greatest constriction of process neck, lateral view: greater (0) or less than (1) 2.0. (Carr and Williamson, 2010:186; Brusatte et al., 2010:134; Loewen et al., 2013:209; Brusatte and Carr, 2016:133).

180) Palatine, vomeropterygoid process, orientation of neck, lateral view: inclined rostrodorsally (0), vertical (1) (Carr and Williamson, 2010:187; Brusatte et al., 2010:135; Loewen et al., 2013:213; Brusatte and Carr, 2016:135).

181) Palatine, pneumaticity, lateral view: absent (0), present (1) (Carr and Williamson, 2010:190; Brusatte et al., 2010:136; Loewen et al., 2013:215; Brusatte and Carr, 2016:136).

182) Palatine, pneumatic recess, number of external pneumatic openings, lateral view: none (0), one (1), two (2) (Carr and Williamson, 2010:189; Brusatte et al., 2010:137; Loewen et al., 2013:215; Brusatte and Carr, 2016:137).

183) Palatine, primary external opening of palatine recess, location of caudal margin, lateral view: level with or extends caudal to (0) or located far rostral to (1) caudal margin of the vomeropterygoid process neck (Carr and Williamson, 2010:192; Brusatte et al., 2010:138; Loewen et al., 2013:217; Brusatte and Carr, 2016:138).

184) Palatine, primary opening of palatine recess, location of rostral margin, lateral view: level with or caudal to (0) or rostral to (1) rostral margin of the vomeropterygoid process neck (Carr and Williamson, 2010:193; Brusatte et al., 2010:139; Brusatte and Carr, 2016:139).

185) Palatine, jugal process, location of contact surface for lacrimal, lateral view: caudal ("distal"), separated from opening of palatine recess by wide margin (0); rostral ("proximal"), closely approaches opening of palatine recess (1) (Carr and Williamson, 2010:194; Brusatte et al., 2010:140; Loewen et al., 2013:222; Brusatte and Carr, 2016:140).

186) Palatine, maxillary process, joint surface for the maxilla, form, lateral view: flat (0); deeply excavated as a slot, demarcated dorsally by a pronounced lip of bone (1) (Carr and Williamson,

2010:195; Brusatte et al., 2010:141; Loewen et al., 2013:219; Brusatte and Carr, 2016:141).

187) Palatine, inflation of jugal process, lateral and dorsal views: absent (0); present, process visibly inflated (1) (var. Holtz, 2001:70; var. Currie et al., 2003:44; Carr and Williamson, 209:197; Brusatte et al., 2010:142; Loewen et al., 2013:223; Brusatte and Carr, 2016:142).

188) Palatine, maxillary articulation, brace, presence: maxilla abuts lateral surface of maxillary process and rostral region of jugal process (0); contact reinforced by a "brace" at the rostroventral corner of the jugal process, which sits within internal antorbital fossa (1) (Carr and Williamson, 2010:196; Brusatte et al., 2010:143; Loewen et al., 2013:220; Brusatte and Carr, 2016:143).

189) Palatine, maxillary articulation brace, form, lateral view: projects ventrally due to a jugal process that extends further ventrally than the maxillary process, such that there is a discrete corner between the two processes in lateral view (0); projects laterally, with no discrete corner between the smoothly confluent jugal and maxillary processes in lateral view (1) (Brusatte et al., 2010:144; Loewen et al., 2013:221; Brusatte and Carr, 2016:144).

PALATAL OPENINGS (2 characters)

190) Internal choana, shape, lateral, dorsal, medial, and ventral views: rostrocaudally long oval (0), nearly circular (1) (Brusatte et al., 2010:145; Loewen et al., 2013:205; Brusatte and Carr, 2016:145).

191) Suborbital fenestra, shape, ventral view: rostrocaudally long oval (0), nearly circular (1) (Brusatte et al., 2010:146; Loewen et al., 2013:206; Brusatte and Carr, 2016:146).

BRAINCASE (34 characters)

192) Braincase, orientation of occipital surface, caudal view: faces caudally (0) or caudoventrally (1) (Holtz, 2001:65; Currie et al., 2003:63; Carr and Williamson, 2010:3; Sereno et al., 2009:46; Brusatte et al., 2010:147; Loewen et al., 2013:186; Brusatte and Carr, 2016:147).

193) Supraoccipital, contribution to dorsal rim of foramen magnum: forms entire rim (0), makes limited contribution to rim via triangular ventral process (1), completely excluded from rim (2) (Holtz, 2001:66; var. Currie et al., 2003:12; Carr and Williamson 2010:158; Brusatte et al., 2010:148; Loewen et al., 2013:168; Brusatte and Carr, 2016:148).

194) Supraoccipital, form of dorsal margin, caudal view: smoothly convex and undivided (0), divided into two processes ("forked") (1) (Carr and Williamson, 2010:170; Brusatte et al., 2010:149; Loewen et al., 2013:162; Brusatte and Carr, 2016:149).

195) Supraoccipital, form, caudal view: pronounced midline crest with a deep fossa on each side or flat plate that abuts the caudal surface of the parietal (0), flat and expands dorsally into tab-like processes (1) (Bakker et al., 1988; Holtz, 2001:8; Currie et al., 2003:71; Carr and Williamson, 2010:172; Brusatte and Carr 2016:319; Brusatte et al., 2016).

196) NEW Supraoccipital, ventral process, shape, caudal view: tiny and squared off

process (0), process is absent (1), large, diamond-shaped process (2) (Brusatte et al., 2016).

197) NEW Supraoccipital, pneumaticity: apneumatic (0), pneumatic (1) (Brusatte et al., 2016).

198) Supraoccipital, midline ridge on caudal surface, presence, caudal view: ridge is present and strongly demarcated (0), absent (1) (Loewen et al., 2013:166; Brusatte and Carr, 2016:347).

199) Otoccipital, paroccipital process, ventral flange at distal end, caudal and lateral views: absent (0), present (1) (Brusatte et al., 2010:150).

200) Otoccipital, paroccipital processes, deep fossa on caudal surface dorsolateral to the foramen magnum, caudal view: absent (0), present (1) (Brusatte et al., 2010:151; Loewen et al., 2013:177; Brusatte and Carr, 2016:151).

201) Otoccipital, crista tuberalis (=metotic strut), extent, caudal view: limited, mediolateral width across opposing cristae less than one half the dorsoventral depth of the braincase from the dorsal tip of the supraoccipital to the ventral tip of the basal tubera (0); extensive, width greater than one half the braincase depth (1) (Brusatte et al., 2010:152; Loewen et al., 2013:179; Brusatte and Carr, 2016:152).

202) Otoccipital, fossa for cranial nerves X-XII, depth, caudal view: shallow (0), deep (1) (Brusatte et al., 2010:153; Loewen et al., 2013:178; Brusatte and Carr, 2016:153).

203) NEW Otoccipital, otic recess, enclosure, lateral view: not enclosed by the lamina and as a result having a large external opening of the (0), recess enclosed by the superficial lamina of the prootic (1) (Brusatte et al., 2016).

204) NEW Otoccipital, caudal tympanic recess, presence, lateral view: absent (0), present (1) (var. Carr and Williamson, 2010:154).

205) Basioccipital, basal tubera, dorsoventral depth, caudal view: less than (0) or greater than (1) depth of occipital condyle (Brusatte et al., 2010:154; Loewen et al., 2013:192; Brusatte and Carr, 2016:154).

206) Basioccipital, basal tubera, concave notch ventrally between opposing tubera, dorsoventral depth, caudal view: shallow, less than 40% depth of tubera (0); deep, approximately 50% depth of tubera (1) (Brusatte et al., 2010:155; Loewen et al., 2013:194; Brusatte and Carr, 2016:155).

207) Basioccipital, subcondylar recess, depth of pneumatic fossae on caudal surface of basal tubera, caudal view: absent or shallow (0), deep (1) (Carr and Williamson, 2010:166; Brusatte et al., 2010:156; Loewen et al., 2013:188; Brusatte and Carr, 2016:156).

208) Basisphenoid, basipterygoid recess (pneumatic recess above basipterygoid processes on lateral surface of braincase), presence, lateral view: absent or present as shallow pneumatic fossa (0); present as a large rectangular fenestra (1) (Carr and Williamson, 2010:158; Brusatte et

al., 2010:157; Loewen et al., 2013:198; Brusatte and Carr, 2016:157).

209) Basisphenoid, basisphenoid recess, closure and orientation of central axis, caudal views: open and vertical, recess obscured in caudal view (0); open and caudoventral, recess partially visible (1); closed and extremely caudoventral, recess compressed rostrocaudally and widely visible, and basipterygoid processes located beneath the basal tubera (2) (Bakker et al., 1988; Carr, 1999; var. Currie et al., 2003:7; Sereno et al., 2009:50; Carr and Williamson, 2010:161-163; Brusatte et al., 2010:158; Loewen et al., 2013:202; Brusatte and Carr, 2016:158). ORDERED.

210) Basisphenoid, basisphenoid recess, inflation of the ceiling of the recess, ventral view: absent (0); present (1) (Carr and Williamson, 2010:164; Brusatte et al., 2010:159; Loewen et al., 2013:201; Brusatte and Carr, 2016:159).

211) Basisphenoid recess, shape, ventral view: funnel-like, expands in mediolateral width caudally (0); ovoid or circular, no posterior expansion (1) (Brusatte et al., 2010:160; Brusatte and Carr, 2016:160).

212) Basicranium, shape (rectangle defined by positions of both basal tubera and both basipterygoid processes), ventral view: rostrocaudally longer than mediolaterally wide (0), wider than long (1) (Bakker et al., 1988; Currie et al., 2003:8; Sereno et al., 2009:49; Carr and Williamson, 2010:167; Li et al. 2010: 272; Brusatte et al., 2010:161; Brusatte and Carr, 2016:161).

213) Sphenoid rostrum, shape, lateral view: rostrocaudally long, ventral margin is a smooth concave arch (0); dorsoventrally tall, ventral margin is nearly vertical caudally and then abruptly transitions to horizontal trend rostrally (1) (Brusatte et al., 2010:162; Loewen et al., 2013:203; Brusatte and Carr, 2016:162).

214) Prootic, dorsal tympanic recess, presence, lateral view: present (0), absent (1) (Brusatte et al., 2010:163; Loewen et al., 2013:169; Brusatte and Carr, 2016:163).

215) NEW Prootic, prootic fossa, presence, lateral view: absent (0), present (1) (Brusatte et al., 2016).

216) Laterosphenoid, antotic crest separating lateral wall of braincase from orbital and temporal spaces, presence and form, lateral and rostrolateral views: absent or indistinct (0), present and robust and rugose (1) (Carr and Williamson, 2010:152, 155; Brusatte et al., 2010:164; Loewen et al., 2013:182; Brusatte and Carr, 2016:164).

217) Laterosphenoid, antotic crest, form, lateral view: single structure (0), bifurcates ventrally (1) (Brusatte et al., 2010:165; Loewen et al., 2013:183; Brusatte and Carr, 2016:165).

218) Laterosphenoid, concave joint surface on lateral surface for the head of epipterygoid, presence, lateral view: absent or shallow (0); present, deep and rugose (1) (Brusatte et al., 2010:166; Brusatte and Carr, 2016:166).

219) Laterosphenoid, mediolaterally oriented ridge on dorsolateral surface that extends laterally to the edge of the dorsotemporal fenestra, presence, dorsal view: absent (0), present (1) (Carr and Williamson, 2010:150).

220) Laterosphenoid, ventrolateral shelf, presence, lateral view: absent (0), present (1) (Carr and Williamson, 2010:153; Loewen et al., 2013:185; Brusatte and Carr, 2016:349).

221) Ossified sphenethmoid and mesethmoid (when not fossilized, can be inferred by presence of scars on ventral surface of frontal), lateral and ventral views: absent (0), present (1) (Brusatte et al., 2010:167; Brusatte and Carr, 2016:167).

222) NEW Endocast, root of trigeminal nerve, bifurcation, location: splits within prootic such that there is one trigeminal foramen in the endocranial surface (0), splits within the endocranial cavity such that there are two trigeminal foramina in the endocranial surface (1) (Brusatte et al., 2016).

223) NEW Endocast, midbrain flexure, condition: prominent (0), reduced (1) (Brusatte et al., 2016).

224) NEW Endocast, midbrain peak, presence: absent (0), present (1) (Osborn, 1912; Witmer and Ridgley, 2009; Brusatte et al., 2016).

225) NEW Endocast, cochlear duct, length: short (0), long (1) (Witmer and Ridgely, 2009; Brusatte et al., 2016).

LOWER JAW (3 characters)

226) Mandibular ramus, dorsoventral depth of dentary at level of dentary-surangular contact on the dorsal margin of the lower jaw, lateral view: less (0) or greater than (1) 18% of the total rostrocaudal length of the lower jaw (1) (Bakker et al., 1988; var. Holtz, 2001:72; Carr and Williamson, 2010:198; Brusatte et al., 2010:168; Loewen et al., 2013:234; Brusatte and Carr, 2016:168).

227) External mandibular fenestra, dorsoventral depth relative to depth of mandible at midpoint of fenestra, lateral view: greater (0) or less than (1) 25% (Sereno et al., 2009:51; Carr and Williamson, 2010:215; Brusatte et al., 2010:169; Loewen et al., 2013:235; Brusatte and Carr, 2016:169).

228) Lower jaw, articulation, glenoid position relative to level of alveolar margin of dentary, lateral view: level or ventral (0), dorsal (1) (Sereno et al., 2009:54; Brusatte et al., 2010:170; Loewen et al., 2013:237; Brusatte and Carr, 2016:170).

DENTARY (10 characters)

229) Dentary, position of the transition point between the ventral and rostral margins of the bone, lateral view: below alveoli 1-3, rostral margin rounded (0); below alveolus 4, rostral margin nearly straight and projects caudoventrally (1) (Carr and Williamson, 2010:213; Brusatte et al.,

2010:171; Loewen et al., 2013:253; Brusatte and Carr, 2016:171).

230) Dentary, ventrally projecting rugose process ("chin") where the rostral and ventral margins of the dentary meet, lateral and medial views: absent (0); present, visible as a pointed projection in lateral view and convex in medial view, braces dentary symphysis (1) (Brusatte et al., 2010:172; Brusatte and Carr, 2016:172).

231) Dentary, symphysis, texture, medial view: generally smooth (0); strongly rugose and beveled, with interlocking ridges and convexities for articulation with the opposing symphysis (1) (Brusatte et al., 2010:173; Loewen et al., 2013:243; Brusatte and Carr, 2016:173).

232) Dentary, joint surface for splenial along ventral region of dentary ramus below the Meckelian fossa, form, medial view: dorsoventrally shallow and smooth (0), dorsoventrally deep (nearly as deep as anterior depth of fossa) and rugose (1) (Carr, 1999; Brusatte et al., 2010:174; Loewen et al., 2013:252; Brusatte and Carr, 2016:174).

233) Dentary, mesial alveoli, size in comparison to alveoli in middle of tooth row, dorsal view: approximately same size (0), first two alveoli substantially smaller (1), first alveolus substantially smaller (2) (Brusatte et al., 2010:175; Loewen et al., 2013:254; Brusatte and Carr, 2016:175).

234) Dentary, primary neurovascular foramina, arrangement, lateral view: distinct foramina or set into a shallow groove caudally (0), set into a deep and sharp groove across the middle and caudal regions of the dentary (1) (Brusatte et al., 2010:176; Loewen et al., 2013:244; Brusatte and Carr, 2016:176).

235) Dentary, dorsal margin of bone, profile, lateral view: straight (0), strongly concave (1) (Brusatte et al., 2010:177; Loewen et al., 2013:245; Brusatte and Carr, 2016:177).

236) Dentary, Meckelian groove, form, medial view: dorsoventrally deep and shallowly inset into medial surface of bone (0); dorsoventrally shallow and deeply inset into bone, groove appears as a thin, sharp structure (1) (Brusatte et al., 2010:178; Loewen et al., 2013:249; Brusatte and Carr, 2016:178).

237) Dentary, orientation of rostral margin relative to the dorsal margin of the bone, lateral view: approaches 90 degrees (0), approximately 45 degrees or less (1) (Carr and Williamson, 2010; Loewen et al., 2013:253; Brusatte and Carr, 2016:358).

238) NEW Dentary, ventral bar, rugose knob at the rostral end of the bar that is below or caudoventral to the Meckelian foramen: absent (0), present (1).

SURANGULAR (9 characters)
239) Surangular, caudal surangular foramen, size: absent or small foramen (0); large fenestra, approximately 30% depth of caudal end of surangular (1) (Holtz, 2001:12; Currie et al., 2003:73; Sereno et al., 2009:52; Carr and Williamson, 2010:202, 203; Brusatte et al., 2010:179; Loewen et al., 2013:274; Brusatte and Carr, 2016:179).

240) Surangular, surangular shelf on lateral surface, form, lateral view: low ridge (0), prominent ridge that is offset laterally from the bone but dorsoventrally thin (1), prominent shelf that is dorsoventrally deep (2) (Sereno et al., 2009:53; Carr and Williamson, 2010:207; Brusatte et al., 2010:180; Loewen et al., 2013:272; Brusatte and Carr, 2016:180). ORDERED.

241) Surangular, surangular shelf, position and form, lateral view: placed far dorsal to caudal surangular foramen (0); foramen abuts shelf, but shelf projects laterally and does not overhang foramen (1); shelf projects ventrolaterally to overhang foramen (2) (Carr, 1999; Holtz, 2001:74; Currie et al., 2003:74; Carr and Williamson, 2010:206; Brusatte et al., 2010:181; Loewen et al., 2013:275; Brusatte and Carr, 2016:181). ORDERED.

242) Surangular, surangular shelf, orientation relative to the long axis of the lower jaw, lateral view: rostrodorsal (0), rostroventral (1), straight rostrocaudally (2) (Carr, 1999; Brusatte et al., 2010:182; Loewen et al., 2013:273; Brusatte and Carr, 2016:182).

243) Surangular, fossa caudodorsal to caudal surangular foramen: absent (0), present (1) (Brusatte et al., 2010:183; Loewen et al., 2013:276; Brusatte and Carr, 2016:183).

244) Surangular, adductor muscle attachment site dorsal to surangular shelf, orientation, lateral and dorsal views: faces primarily dorsally (0), faces almost equally dorsally and laterally (1), faces primarily laterally (2) (Brusatte et al., 2010:184; Loewen et al., 2013:277; Brusatte and Carr, 2016:184). ORDERED.

245) Surangular, triangular fossa on the lateral surface of the surangular shelf immediately rostroventral to glenoid, lateral view: absent (0), present (1) (Brusatte et al., 2010:185; Brusatte and Carr, 2016:185).

246) Surangular, fossa on the lateral surface of the bone immediately ventral to, and separated from, the glenoid, lateral view: absent (0), present (1) (Brusatte et al., 2010:186; Brusatte and Carr, 2016:186).

247) Surangular, rostrocaudal length of rostral flange (region ahead of rostral margin of the external mandibular fenestra) compared to overall length of surangular, lateral view: less than (0) or greater than (1) 30% (Brusatte et al., 2010:187; Brusatte and Carr, 2016:187).

ANGULAR (1 character)

248) Angular, ventral margin, form, lateral view: smoothly convex (0); rostral region “flexed” relative to caudal region, such that there is a discrete step between them (1) (Carr, 1999; Brusatte et al., 2010:188; Loewen et al., 2013:266; Brusatte and Carr, 2016:188).

ARTICULAR (2 characters)

249) Articular, mediolateral width of jaw muscle attachment site, dorsal and caudal views: less than (0) or equal to or greater than (1) width of glenoid for articulation with quadrate (Rauhut et al., 2010; Brusatte et al., 2010:189; Brusatte and Carr, 2016:189).

250) Articular, wide smooth non-articular region between glenoid and attachment site for depressor mandibular muscles: present (0); absent (1) (Rauhut et al., 2010; Brusatte et al., 2010:190; Loewen et al., 2013:287; Brusatte and Carr, 2016:190).

SPLENIAL (2 characters)
251) Splenial, rostral mylohyoid foramen, shape and size, lateral and medial views: small circular or ovoid opening (0); large, rostrocaudally ovoid shape (1); extremely large, approximately as deep dorsoventrally as the rostral process of the splenial (2) (Sereno et al., 1998; Currie et al., 2003:66; Carr and Williamson 2010:208; Brusatte et al., 2010:191; Loewen et al., 2013:260; Brusatte and Carr, 2016:191). ORDERED.

252) Splenial, dorsal region overlapped medially by prearticular, medial view: absent (0), present (1) (Brusatte et al., 2010:192; Brusatte and Carr, 2016:192).

PREARTICULAR (2 characters)
253) Prearticular, ventral bar, series of ridges on lateral surface to strengthen articulation with angular, lateral view: absent (0), present (1) (Brusatte et al., 2010:193; Loewen et al., 2013:283; Brusatte and Carr, 2016:193).

254) Prearticular, midshaft, cross section, ratio of mediolateral width to dorsoventral height: less than 0.9 (0), 0.9 or greater (1) (Loewen et al., 2013:281; Brusatte and Carr, 2016:350).

INTERCORONOID (2 characters)
255) Intercoronoid ossification, shape, lateral and medial views: long, shallow strip (0); deep, crescentic shape (1) (Brusatte et al., 2010:194; Loewen et al., 2013:256; Brusatte and Carr, 2016:194).

256) Supradentary and coronoid ossifications, form of contact at their zone of fusion, lateral and medial views: smoothly confluent (0), offset by a concave notch (1) (Brusatte et al., 2010:195; Loewen et al., 2013:257; Brusatte and Carr, 2016:195).

DENTITION (9 characters)
257) Premaxillary teeth, position of mesial carina: offset mesial to distal carina on all teeth (teeth ziphodont) (0); rotated distally on premaxillary teeth 1 and 2 (mesial teeth "D-shaped" and distal teeth ziphodont) (1); rotated distally on all teeth (2). (Holtz, 2001:14; Currie et al., 2003:76; Sereno et al., 2009:56-58; Carr and Williamson 2010:217; Brusatte et al., 2010:196; Loewen et al., 2013:290; Brusatte and Carr, 2016:196). ORDERED.

258) Premaxillary tooth crown 4, apicobasal height relative to largest maxillary crown: subequal (0), approximately 50% (1) (var. Holtz, 2001:15; var. Currie et al., 2003:75; var. Carr and Williamson, 2010:216; Sereno et al. 2009:59; Brusatte et al., 2010:197; Brusatte and Carr, 2016:197).

259) Premaxillary teeth, median vertical ridge on lingual surface: absent (0), present as subtle structure in mesial premaxillary teeth (1), present as pronounced structure in all premaxillary teeth (2) (Holtz, 2001:77; Sereno et al. 2009:60; Brusatte et al., 2010:198; Loewen et al.,

2013:291; Brusatte and Carr, 2016:198). ORDERED.

260) Premaxillary teeth, curvature of distal teeth, lingual or labial views: curved (0), straight (1) (Brusatte et al., 2010:199; Loewen et al., 2013:292; Brusatte and Carr, 2016:199).

261) Maxillary teeth, number: 13 or more (0), less than 13 in the largest adult specimens (1) (Bakker et al., 1988; var. Holtz, 2001:45; Sereno et al., 2009:61; Carr and Williamson 2010:218; Brusatte et al., 2010:200; Loewen et al., 2013:298; Brusatte and Carr, 2016:200).

262) Maxillary teeth, carinae, position: symmetrical, with mesial and distal carinae on opposite sides of a lens-shaped crown (0); asymmetrical, with one or both carinae not centered on its side (1) (Loewen et al., 2013:296; Brusatte and Carr, 2016:251).

263) Dentary teeth, number: 17 or less (0), 18 or more (1) (Bakker et al., 1988; var. Holtz, 2001:75; var. Carr and Williamson, 2010:219; Lu et al., 2014; Loewen et al., 2013:300; Brusatte and Carr, 2016:315).

264) Maxillary and dentary teeth, form: ziphodont, labiolingual width of base less than 60% of mesiodistal length (0); incrassate, width greater than 60% of length (1); incrassate, width nearly equal to length (2) (Holtz, 2001:79; Sereno et al., 2009:62; Brusatte et al., 2010:201; Loewen et al., 2013:303; Brusatte and Carr, 2016:201). ORDERED.

265) Maxillary and dentary teeth, mesial and distal denticles, size: not substantially different in size (0); mesial denticles, when present, substantially smaller than distal denticles (1) (Loewen et al., 2013:302; Brusatte and Carr, 2016:352).

CERVICAL VERTEBRAE (17 characters)

266) Axis and postaxial cervicals, anteroposterior length of centrum compared to dorsoventral height of posterior centrum face: greater (0); less than or equal to (1) (Holtz, 2001:80; Brusatte et al., 2010:202; Loewen et al., 2013:307; Brusatte and Carr, 2016:202).

267) Axis, pneumatic foramen (pleurocoel), position, lateral view: near midheight of centrum (0); dorsally located, directly underneath neurocentral suture and directly posterior to diapophysis (1) (Brusatte et al., 2010:203; Loewen et al., 2013:305; Brusatte and Carr, 2016:203).

268) Axis, pneumatic foramen, extent of surrounding fossa, lateral view: limited to margins of foramen (0); extensive, occupies most of lateral surface of centrum (1) (Brusatte et al., 2010:204; Loewen et al., 2013:306; Brusatte and Carr, 2016:204).

269) Axis, ridge on ventral surface of axis, ventral view: absent (0), present (1) (Brusatte et al., 2010:205; Loewen et al., 2013:308; Brusatte and Carr, 2016:205).

270) Axis, pneumatic foramina and fossae on each side of the anterior ridge on the neural spine, anterior view: absent (0), present (1) (Brusatte et al., 2010:206; Loewen et al., 2013:312; Brusatte and Carr, 2016:206).

271) Axis, neural spine, texture of dorsal region of anterior surface, anterior view: generally smooth or with subtle texture (0); highly rugose, with series of grooves, ridges, and eminences (1) (Brusatte et al., 2010:207; Loewen et al., 2013:313; Brusatte and Carr, 2016:207).

272) Axis, dorsal region of neural spine, number of projections on "crown" region, anterior view: two lateral projections, dorsal surface of spine smoothly concave (0); two lateral projections and one dorsal projection on the midline (1) (Brusatte et al., 2010:208; Loewen et al., 2013:314; Brusatte and Carr, 2016:208).

273) Axis, spinous process, form, lateral view: sheet-like (0), anteroposteriorly reduced and rod-like (1) (Loewen et al., 2013:310; Brusatte and Carr, 2016:353).

274) Axis, supradiapohyseal fossa (fossa posterodorsal to diapophysis), lateral view: absent or shallow (0), deeply excavated and funnel-like (1) (Brusatte et al., 2010:209; Brusatte and Carr, 2016:209).

275) Axis and anterior-middle postaxial cervicals, epipophysis, form A, lateral and dorsal views: extends only slightly posterior to postzygapophysis (0), extends far posterior to postzygapophysis (1) (Brusatte et al., 2010:210; Loewen et al., 2013:315; Brusatte and Carr, 2016:210).

276) Axis and anterior-middle postaxial cervicals, epipophysis, form B, lateral and dorsal views: small, pyramidal mound (0); large, rugose flange (1) (Brusatte et al., 2010:210; Loewen et al., 2013:315; Brusatte and Carr, 2016:210).

277) Cervical vertebrae, spinous processes in middle-posterior cervicals, shape, dorsal view: long rectangle, much longer anteroposteriorly than wide transversely (0); short rectangle, base is essentially square shaped due to nearly equal anteroposterior and transverse dimensions (1) (Brusatte et al., 2010:211; Loewen et al., 2013:325; Brusatte and Carr, 2016:211).

278) Cervical vertebrae, spinous processes in middle-posterior cervicals, dorsoventral height, lateral view: substantially shorter (0); or approximately the same height as or taller (1) than posterior centrum face (Holtz, 2001:81; Brusatte et al., 2010:212; Loewen et al., 2013:324; Brusatte and Carr, 2016:212).

279) Cervical vertebrae, posterior centrodiapophyseal laminae in anterior-middle cervicals, form, lateral view: absent or present as a weak ridge (0); present as a thick, laterally offset lamina that demarcates a deep infradiapophyseal fossa (1) (Brusatte et al., 2010:213; Loewen et al., 2013:327; Brusatte and Carr, 2016:213).

280) Cervical vertebrae, hypapophysis on anterior region of ventral surface, presence, anterior and ventral views: absent (0), present (1) (Brusatte et al., 2010:214; Brusatte and Carr, 2016:214).

281) Cervical vertebrae, prezygapophysis in middle cervicals, position, dorsal view: slightly

overhangs centrum laterally (0); strongly overhangs centrum laterally, entire prezygapophyseal facet placed lateral to centrum (1) (Brusatte et al., 2010:215; Loewen et al., 2013:328; Brusatte and Carr, 2016:214).

282) Cervical vertebrae, posterior centrodiapophyseal lamina in anterior-middle cervicals, orientation, lateral view: projects posteroventrally, infrapostzygapophyseal fossa located primarily posterior to lamina (0); nearly horizontal, fossa located primarily dorsal to lamina (1). (Brusatte et al., 2010:216; Loewen et al., 2013:327; Brusatte and Carr, 2-16:216).

DORSAL VERTEBRAE (6 characters)

283) Cervical and dorsal vertebrae, rugose ligament attachment scars in pre- and postspinal fossae, anterior, posterior, and lateral views: absent or weakly developed (0); present as prominent, rectangular flanges that extend outside of the fossae and are visible in posterior view, but on the dorsal vertebrae only (1); prominent in dorsals and cervicals (2) (Brusatte et al., 2010:217; Loewen et al., 2013:335; Brusatte and Carr, 2016:217). ORDERED.

284) Dorsal vertebrae, pneumaticity, presence of pneumatic foramina on lateral centrum surface, lateral view: absent or limited to anterior dorsals (0), present throughout dorsal column (1) (Carr and Williamson, 2010:222; Brusatte et al., 2010:218; Loewen et al., 2013:329; Brusatte and Carr, 2016:218).

285) Dorsal vertebrae, spinous process, level of posterior termination: at approximately the same level as the posterior centrum face (0), far posterior to the posterior centrum face (1) (Carr and Williamson, 2010:223; Brusatte et al., 2010:219; Loewen et al., 2013:331; Brusatte and Carr, 2016:219).

286) Dorsal vertebrae, anteroposterior length of middle-posterior dorsal centra compared to dorsoventral height of posterior centrum face, lateral view: greater (0), less than or equal (1) (var. Carr and Williamson 2010: 224; Brusatte et al., 2010:220; Loewen et al., 2013:330; Brusatte and Carr, 2016:220).

287) Dorsal vertebrae, middle-posterior dorsals, position of postzygapophysis relative to prezygapophysis, lateral view: at same level (0), elevated dorsally (1) (Brusatte et al., 2010:221; Brusatte and Carr, 2016:221).

288) Dorsal vertebrae, middle-posterior dorsals, form of anterior and posterior centrodiapophyseal laminae, ventral view: make contact on ventral surface of transverse process, demarcating a triangular infradiapophyseal fossa (0); do not make contact but roughly parallel each other, infraprezygapophyseal and infradiapophyseal fossae merged into a single fossa (1) (Brusatte et al., 2010:222; Brusatte and Carr, 2016:222).

SACRAL VERTEBRAE (6 characters)

289) Sacral vertebrae, pneumatic foramina on lateral surfaces of centra, presence, lateral view: absent (0), present (1) (Carr and Williamson 2010:225; Brusatte et al., 2010:223; Loewen et al., 2013:341; Brusatte and Carr, 2016:223).

290) Sacral vertebrae, fenestrae between fused neural spines: neural spines unfused (0); spines fused but fenestrae absent (1); spines fused and fenestrae present (2) (Brusatte et al., 2010:224; Loewen et al., 2013:342; Brusatte and Carr, 2016:224). ORDERED.

291) Sacral ribs, position of central ribs on sacrum, lateral, dorsal, and ventral views: span two sacrals (0); limited to a single sacral (1) (Brusatte et al., 2010:225; Loewen et al., 2013:343; Brusatte and Carr, 2016:225).

292) Sacral ribs, position of rib attachment for central ribs on individual sacrals, lateral view: span centrum and neural arch (0); limited to neural arch only (1) (Brusatte et al., 2010:226; Loewen et al., 2013:344; Brusatte and Carr, 2016:226).

293) Sacral vertebra five, position of ventral margin of posterior articular face, lateral view: at same level as (0) or positioned ventral to (1) ventral margin of anterior articular face (Brusatte et al., 2010:227; Loewen et al., 2013:345; Brusatte and Carr, 2016:227).

294) Sacral vertebra five, form of hyposphene in posteriormost sacral, posterior view: absent or present as a single midline structure (0); present and comprised of two parallel-sided sheets (1) (Brusatte et al., 2010:228; Brusatte and Carr, 2016:228).

CAUDAL VERTEBRAE (3 characters)

295) Caudal vertebrae, anterior caudals, position of base of neural spine, lateral view: anterior to (0) or level with or posterior to (1) posterior surface of centrum (Brusatte et al., 2010:229; Loewen et al., 2013:349; Brusatte and Carr, 2016:229).

296) Caudal vertebrae, anterior caudals, shape of transverse processes, dorsal and ventral views: rectangular, with parallel anterior and posterior sides, or slightly ovoid with a gradual expansion in width distally (0); distal end expanded into a spatulate flange (1) (Brusatte et al., 2010:230; Loewen et al., 2013:350; Brusatte and Carr, 2016:230).

297) Caudal vertebrae, anterior caudals, two laminae linking prezygapophysis and transverse process, between which is a deep, triangular fossa, presence, lateral view: absent (0), present (1) (Brusatte et al., 2010:231; Loewen et al., 2013:351; Brusatte and Carr, 2016:231).

SCAPULA (5 characters)

298) Scapula, angle between posterior margin of glenoid and dorsal margin of blade, lateral view: greater (0) or approximately (1) 90 degrees (Sereno et al., 2009:68; Carr and Williamson, 2010:230; Brusatte et al., 2010:232; Loewen et al., 2013:365; Brusatte and Carr, 2016:232).

299) Scapula, acromion, dorsoventral depth: less (0) or greater (1) than 3.0 times minimum dorsoventral depth of blade (Sereno et al., 2009:67; Brusatte et al., 2010:233; Brusatte and Carr, 2016:233).

300) Scapula, ratio of anteroposterior length of bone to minimum dorsoventral depth of blade, lateral view: less (0) or greater (1) than 10.0 (Sereno et al., 2009:69; Brusatte et al., 234; Loewen et al., 2013:363; Brusatte and Carr, 2016:234).

301) Scapula, ratio of dorsoventral depth of distal expansion to minimum dorsoventral depth of blade, lateral view: less (0) or greater (1) than 2.5 (Holtz, 2001:82; Sereno et al., 2009:70; Brusatte et al., 2010:235; Loewen et al., 2013:367; Brusatte and Carr, 2016:235).

302) Scapula and coracoid, glenoid, position relative to posteroventral margin of blade: offset posteroventrally (approximately by the width of the neck of the blade) (0); offset only slightly posteroventrally (less than 50% the width of the neck of the blade) (1) (Sereno et al., 2009:66; Brusatte et al., 2010:236; Brusatte and Carr, 2016:236).

CORACOID (2 characters)

303) Coracoid, anteroposterior length at midpoint, lateral view: approximately 100-150% (0) or 200% or greater (1) than the length of the scapular acromion at midheight. (Sereno et al., 2009:71; Brusatte et al., 2010:237; Loewen et al., 2013:372; Brusatte and Carr, 2016:237).

304) Coracoid, coracoid foramen, lateral view: present (0), absent or extremely small (1) (Brusatte et al., 2010:238; Brusatte and Carr, 2016:238).

HUMERUS (7 characters)

305) Humerus, length relative to the femur: 50-70% (0), 40-30% (1), 25-30% (2) (var. Holtz, 2001:20; Sereno et al., 2009:72; Carr and Williamson 2010:232; Brusatte et al., 2010:239; Loewen et al., 2013:383; Brusatte and Carr, 2016:239). ORDERED.

306) Humerus, head, form, proximal view: low, poorly differentiated, crescentic shaped, overhangs posterior surface and does not overhang anterior surface (0); enlarged, occupies the majority of the proximal end, bulbous in proximal view, overhangs both posterior and anterior surfaces (1) (Sereno et al. 2009:73; Brusatte et al., 2010:240; Loewen et al., 378; Brusatte and Carr, 2016:240).

307) Humerus, apex of deltopectoral crest, location from proximal end, lateral view: 40-50% (0), 25-35% (1), less than 25% (2) of the length of the humerus (Sereno et al., 2009:74; Brusatte et al., 2010:241; Loewen et al., 2013:379; Brusatte and Carr, 2016:241). ORDERED.

308) Humerus, rotation along shaft, orientation of long axis of proximal end relative to that of distal end, proximal and distal views: approximately 30-45 degree angle (0), approximately parallel, shaft rotation absent (1) (Carr and Williamson, 2010:232; Brusatte et al., 2010:242; Loewen et al., 2013:377; Brusatte and Carr, 2016:242).

309) Humerus, additional muscle attachment tubera at the corner of the anterior and lateral surfaces distal to the deltopectoral crest, anterolateral view: absent (0); present (1) (Brusatte et al., 2010:243; Loewen et al., 381; Brusatte and Carr, 2016:243).

310) Humerus, concave notch between external tuberosity and deltopectoral crest, anterior and posterior views: present, two structures clearly separated (0); absent, two structures smoothly confluent (1) (Brusatte et al., 2010:244; Brusatte and Carr, 2016:244).

311) Humerus, form of distal condyles, anterior view: lateral and medial condyles expanded equally (offset from shaft in anterior or posterior view is equal) (0), medial condyle expanded further medially than the lateral condyle is laterally (1) (Brusatte et al., 2010: 245; Loewen et al., 2013:382; Brusatte and Carr, 2016:245).

ULNA (2 characters)
312) Ulna, shaft axis, form, lateral view: bowed (0), straight (1) (Sereno et al., 2009:75; var. Carr and Williamson, 2010:233; Brusatte et al., 2010:246; Loewen et al., 2013:384; Brusatte and Carr, 2016:246).

313) Ulna, distal articular surface, form, distal view: convex (0); flat (1) (Sereno et al., 2009:76; Brusatte et al., 2010:247; Loewen et al., 2013:384; Brusatte and Carr, 2016:247).

CARPUS AND MANUS (9 characters)
314) Principal distal carpal, shape, lateral and proximal views: semilunate in lateral view with trochlear proximal surface (0); discoid with flat proximal surface (1) (Carr and Williamson, 2010:238; Li et al. 2010: 276; Brusatte et al., 2010:248; Loewen et al., 2013:386; Brusatte and Carr, 2016:248).

315) Manus, number of functional digits, dorsal and ventral views: three or more (0); two, metacarpal III is absent or rudimentary, without phalanges (1) (var. Holtz, 2001:22; Sereno et al., 2009: 82; Carr and Williamson, 2010:235; Brusatte et al., 2010:249; Loewen et al., 2013:388; Brusatte and Carr, 2016:249).

316) Metacarpal I, medial distal condyle, form, distal view: well formed and large (0), rudimentary (1) (Sereno et al., 2009:78; Brusatte et al., 2010:350; Loewen et al., 2013:389; Brusatte and Carr, 2016:250).

317) Metacarpal I, medial margin, shape, proximal view: concave (0); smooth convexity (1) (Brusatte et al., 2010:251; Brusatte and Carr, 2016:251).

318) Metacarpals, ratio of the length of metacarpal II:length of metacarpal I, dorsal and ventral views: 2-1.8 (0); 1.8-1.6 (1) (Holtz, 2001:84; Sereno et al. 2009:79; Brusatte et al., 2010:252; Loewen et al., 390; Brusatte and Carr, 2016:252).

319) Metacarpals, metacarpal II, mediolateral width at midpoint: equal to or narrower than (0); or more robust than (1) metacarpal I (Carr and Williamson, 2010:236; Brusatte et al., 2010:253; Loewen et al., 2013:391; Brusatte and Carr, 2016:253).

320) Manual phalanx II-1, length, dorsal and ventral views: longer (0); or subequal to (1) the length of metacarpal 1 (Sereno et al., 2009:81; Brusatte et al., 2010:254; Loewen et al., 2013:394; Brusatte and Carr, 2016:254).

321) Manual unguals, flexor tubercle, form, lateral and medial views: large, robust, rugose, conical structure (0); reduced to a small convexity (1) (Brusatte et al., 2010:255; Brusatte et al., 2010:255; Loewen et al., 2013:395; Brusatte and Carr, 2016:255).

322) Manual unguals, degree of recurvature, lateral and medial views: extensive, flexor margin deeply concave (0); reduced, flexor margin shallowly concave (1) (Brusatte et al., 2010:256; Loewen et al., 2013:396; Brusatte and Carr, 2016:256).

ILIUM (22 characters)
323) Ilium, anteroposterior length compared to length of femur, lateral view: 70-85% (0); 95-105% (1); 105-115% (2) (Holtz, 2001:23; Sereno et al., 2009:83; Carr and Williamson, 2010:247; Brusatte et al., 2010:257; Loewen et al., 2013:400; Brusatte and Carr, 2016:257). ORDERED.

324) Ilium, linear ridge dorsal to the acetabulum on the lateral surface of the blade, lateral view: absent or low convexity (0); present (1) (Holtz, 2001:28; Sereno et al., 2009:84; Carr and Williamson, 2010:242; Brusatte et al., 2010:258; Loewen et al., 2013:409; Brusatte and Carr, 258).

325) Ilium, linear ridge dorsal to the acetabulum on the lateral surface of the blade, orientation, lateral view: vertical or anterodorsally extending (0); posterodorsally oriented (1) (Carr and Williamson, 2010:243, 244; Loewen et al., 2013:409; Brusatte and Carr, 2016:324).

326) Ilium, preacetabular process, anteroventral corner, form, lateral view: subtriangular (0); subquadrate with recurved anterior margin, projecting farther anteriorly than remainder of anterior end ("hook" present) (1) (Holtz, 2001:25; Sereno et al., 2009:87; Carr and Williamson, 2010:245; Brusatte et al., 2010:259; Loewen et al., 2013:406; Brusatte and Carr, 2016:259).

327) Ilium, dorsal margin of blade, position relative to sacral neural spines, dorsal view: separated by a gap (0), lies against neural spines and opposing iliac blades may make contact above neural spines in some individuals (1) (Holtz, 2001:27; Sereno et al., 2009:85; Brusatte et al., 2010:260; Loewen et al., 2013:402; Brusatte and Carr, 2016:260).

328) Ilium, acetabular antitrochanter at posterior end of supraacetabular shelf, lateral view: absent or limited in extent (0); present and visible in lateral view as an extensive flange, which is deeply inset from the remainder of the ischial peduncle (1) (Holtz, 2001:111; Sereno et al., 2009:90; Brusatte et al., 2010:261; Brusatte and Carr, 2016:261).

329) Ilium, acetabular crest, maximum lateral projection relative to ischial peduncle, dorsal and ventral views: significantly greater (0); subequal (1) (Holtz, 2001:29; Sereno et al., 2009:91; Brusatte et al., 2010:262; Loewen et al., 2013:414; Brusatte and Carr, 2016:262).

330) Ilium, acetabular crest, extent on pubic peduncle, lateral view: extensive, extends along most or all of the edge of the peduncle (0); limited, discretely offset from acetabular edge of pubic peduncle (1) (Li et al., 2010:278; Brusatte et al., 2010:263; Loewen et al., 2013:413; Brusatte and Carr, 2016:263).

331) NEW Ilium, acetabulum, rostrodorsal margin, exposure, lateral view: entire acetabulum is exposed to view (0), rostrodorsal corner is concealed by massive and hood-

like acetabular crest (1).

332) Ilium, pubic and ischial peduncles, anteroposterior lengths at dorsal base, lateral view: pubic peduncle much larger than ischial peduncle (0); both peduncles approximately the same length (1) (Li et al., 2010:292; Brusatte et al., 2010:264; Loewen et al., 2013:419; Brusatte and Carr, 2016:264).

333) Ilium, pubic peduncle, joint surface, orientation relative to the dorsal margin of the bone: essentially parallel, such that it is nearly horizontal when the dorsal edge of the blade is held horizontally (0); at an angle, such that the joint surface faces anterodorsally when the dorsal edge of the blade is held horizontally (1) (Loewen et al., 2013:416; Brusatte and Carr, 2016:354).

334) Ilium, postacetabular process, ventral margin, shape, lateral view: straight to slightly convex (0); highly convex, forming a discrete "lobe"-like flange (1) (Brusatte et al., 2010:265; Brusatte and Carr, 2016:265).

335) Ilium, dorsal margin, shape, lateral view: smoothly convex or flat across entire length (0); convex anteriorly and straightens out posteriorly (1) (Brusatte et al., 2010:266; Loewen et al., 2013:399; Brusatte and Carr, 2016:266).

336) Ilium, postacetabular process, form of posterior margin, lateral view: tapering, posterior margin convex (0); squared-off, posterior margin vertical (1) (Holtz, 2001:102; Brusatte et al., 2010:267; Loewen et al., 2013:411; Brusatte and Carr, 2016:267).

337) Ilium, ratio of anteroposterior length to dorsoventral depth above acetabulum, lateral view: subrectangular shape, greater than 3.00, ilium is long and low (0); less than 2.8, ilium is subovoid shape (1) (Benson, 2008; Brusatte et al., 2010:268; Brusatte and Benson, 2013:312; Loewen et al., 2013:398, 401; Brusatte and Carr, 2016:268).

338) Ilium, linear ridge dorsal to acetabulum on the lateral surface of the blade, dorsal extent: elongate, extends to dorsal margin of iliac blade (0); short, terminates ventral to dorsal margin of iliac blade (1) (Benson, 2008; Brusatte and Benson 2013:308; Loewen et al. 2013:410; Brusatte and Carr, 2016:308).

339) Ilium, linear ridge dorsal to acetabulum on the lateral surface of the blade, robustness, lateral view: thick, anteroposterior width of ridge greater than 20% of dorsoventral length of ridge (0); thin, width less than or equal to 20% of length (1) (Brusatte and Benson 2013:309; Brusatte and Carr, 2016:309). Note: Dorsoventral length is measured up until the dorsoventral midpoint of the iliac blade, in order to standardize measurements between taxa that have a dorsoventrally extensive ridge and those that have a shorter ridge that terminates ventral to the dorsal margin of the iliac blade (see character 335).

340) Ilium, brevis fossa, orientation, lateral view: faces primarily ventrally, but it is widely visible in lateral view (0); faces primarily ventrally and medially, and it is obscured in lateral view (1) (Brusatte and Benson 2013:310; Loewen et al., 2013:421; Brusatte and Carr, 2016:310).

341) Ilium, mound-like eminence on lateral surface of pubic peduncle, lateral view: absent (0); present (1) (Benson, 2008; Brusatte and Benson 2013:311; Brusatte and Carr, 2016:311).

342) Ilium, width of notch between preacetabular process and pubic peduncle, lateral view: wide, widens anteriorly when seen in lateral view (0); narrow, remains narrow across its entire length when seen in lateral view (1) (Benson, 2008; Brusatte and Benson 2013:313; Loewen et al., 2013:408; Brusatte and Carr, 2016:312).

343) Ilium, anterodorsal notch, presence: absent (0), present (1) (Holtz, 2001:26; Carr and Williamson, 2010:240; Loewen et al., 2013:405).

PUBIS (9 characters)
344) Pubis, shaft, anterior margin, lateral view: straight or convex (bowed anteriorly) (0); concave (bowed posteriorly (1) (Carr and Williamson 2010:248; Brusatte et al., 2010:269; Loewen et al., 2013:423; Brusatte and Carr, 2016:269).

345) Pubis, pubic tubercle, presence and form, lateral view: absent (0); present as a convexity on the anterior margin of the pubis (1); present as a rugose flange that is discretely offset from the anterior margin of the pubis and is bordered posteriorly by heavy rugosities on the lateral surface on the obturator region of the pubis (2) (Carr and Williamson 2010:251; Brusatte et al., 2010:270; Loewen et al., 2013:432; Brusatte and Carr, 2016:270). ORDERED.

346) Pubis, pubic tubercle, position, lateral view: distally positioned, located ventral to the level of the obturator notch (0); proximally positioned, located level with or dorsal to the obturator notch (1) (Brusatte et al., 2010:271; Loewen et al., 2013:433; Brusatte and Carr, 2016:271).

347) Pubis, pubic boot, anteroposterior length relative to total long axis length of pubis, lateral view: less than (0) or greater than (1) 60% (Holtz, 2001:30; Brusatte et al., 2010:272; Loewen et al., 2013:427; Brusatte and Carr, 2016:272).

348) Pubis, pubic boot, anterior ramus, length relative to posterior ramus: 10-40% (0); subequal (1) (Carr and Williamson, 2010: 253, 254; Sereno et al., 2009: 97; Brusatte et al., 2010:273; Loewen et al., 2013:426; Brusatte and Carr, 2016:273).

349) Pubis, pubic boot, position of anterior process relative to posterior process, lateral view: displaced dorsally, resulting in a highly convex ventral margin of the boot (0); placed at the same level, ventral margin of the boot essentially straight (1) (Carr and Williamson, 2010:255; Brusatte et al., 2010:274; Brusatte and Carr, 2016:274).

350) Pubis, orientation of shaft relative to long axis of ilium when pelvis is in articulation, lateral view: perpendicular, shaft approximately vertical (0); oblique, shaft is propubic (1) (Brusatte et al., 2010:275; Loewen et al., 2013:422; Brusatte and Carr, 2016:275).

351) Pubis, anteroposterior expansion of proximal obturator plate region relative to the anterior edge of the pubis shaft at its midpoint, lateral view: less than (0) or greater than (1) twice the anteroposterior thickness of the shaft at its midpoint (Brusatte et al., 2010:276; Loewen et al.,

2013:422; Brusatte and Carr, 2016:276).

352) Pubis, obturator notch, form: discrete structure, demarcated ventrally by extensive obturator flange (0); essentially absent, no ventral flange (1) (var. Carr and Williamson, 2010:249, 250; Brusatte et al., 2010:277; Loewen et al., 2013:429; Brusatte and Carr, 2016:277).

ISCHIUM (6 characters)
353) Ischium, ischial tubercle ventral to iliac peduncle, lateral view: absent or present as a groove (0); present as a convex bulge on the posterior surface of the ischium (1); present as a rugose, ovoid or triangular flange whose lateral surface is depressed relative to the remainder of the ischium (2) (Holtz, 2001:31; Sereno et al., 2009: 93; Carr and Williamson, 2010:257; Brusatte et al., 2010:278; Loewen et al., 2013:443; Brusatte and Carr, 2016:278). Note: *Aviatyrannus* code changed from ? to 0. ORDERED.

354) Ischium, distal end, expansion relative to midshaft, lateral view: expanded, "foot" present (0); tapering, "foot" absent (1) (Holtz, 2001:33; Sereno et al., 2009:95; Carr and Williamson, 2010:260; Brusatte et al., 2010:279; Loewen et al., 2013:439; Brusatte and Carr, 2016:279).

355) Ischium, midshaft diameter (anteroposterior) relative to pubic midshaft diameter, lateral view: 60-100% or ischium is greater (0); 30-50% (1) (Holtz, 2001:32; Sereno et al., 2009:94; Carr and Williamson, 2010:261; Brusatte et al., 2010:280; Loewen et al., 2013:435; Brusatte and Carr, 2016:280).

356) Ischium, position of apex of obturator flange, distance between apex and proximal end of ischium, lateral view: approximately 40% of ischium length ("distal flange") (0); less than 30% of ischum length ("proximal flange") (1) (Brusatte et al., 2010:281; Loewen et al., 2013:441; Brusatte and Carr, 2016:281).

357) Ischium, position of medial apron, medial view: along posterior margin of shaft (0); along anterior margin of shaft (1) (Brusatte et al., 2010:282; Brusatte and Carr, 2016:282). Note: code for *Aviatyrannis* was changed from ? to 1.

358) Ischium, shaft, curvature, lateral view: roughly straight or slightly curved so that the posterior surface is concave (0); curves so that the anterior surface is concave (1) (Loewen et al., 2013:436; Brusatte and Carr, 2016:355). Note: code for *Aviatyrannis* was changed from ? to 1.

FEMUR (10 characters)
359) Femur, circular scar on posterior surface of shaft distal to fourth trochanter, position, posterior view: absent, low, or positioned approximately centrally on the shaft (0); abuts medial edge of shaft (1) (Carr and Williamson, 2010:264; Brusatte et al., 2010:283; Loewen et al., 2013:452; Brusatte and Carr, 2016:283). ORDERED.

360) Femur, lesser trochanter, height relative to greater trochanter, anterior, lateral, and posterior views: shorter, terminates further distally (0); subequal or slightly taller, the two structures extend to approximately the same level proximally (1) (Holtz, 2001:34; Sereno et al., 2009:98; Carr and Williamson, 2010:265; Brusatte et al., 2010:284; Loewen et al., 2013:449; Brusatte and

Carr, 2016:284).

361) Femur, proximal margin, form, posterior view: approximately straight (0); concave, due to a greater trochanter that is elevated substantially relative to the lateral portion of the proximal surface of the head (1) (Brusatte et al., 2010:285; Brusatte and Carr, 2016:285). ORDERED.

362) Femur, trochanteric fossa on the posterior surface of the head, lateral to the trochanteric fossa, form, posterior view: absent or shallow (0); deep fossa (1); deep, extensive triangular depression that covers most of the posterior surface of the femur proximally and is demarcated medially and ventrally by a pronounced, curving, swollen ridge (2) (Brusatte et al., 2010:286; Loewen et al., 2013:446; Brusatte and Carr, 2016:286). ORDERED.

363) Femur, lesser trochanter, accessory trochanter along the anterior margin, form, lateral view: present as a pronounced flange ventrally, which is distinguishable from the remainder of the lesser trochanter (0); reduced to a subtle convexity, barely distinguishable from the remainder of the trochanter (1) (Brusatte et al., 2010:287; Loewen et al., 2013:450; Brusatte and Carr, 2016:287).

364) Femur, fourth trochanter, position, measurement from proximal margin of head to distal termination of trochanter relative to total length of the femur, posterior view: 35% or less (0); 40% or greater (1) (Brusatte et al., 2010:288; Loewen et al., 2013:451; Brusatte and Carr, 2016:288).

365) Femur, lateral condyle, shape, distal view: circular or ovoid (0); ovoid, but with an anterior bulge that is slightly separated from the remainder of the condyle (1) (Brusatte et al., 2010:289; Brusatte and Carr, 2016:289).

366) Femur, extensor groove on anterior surface of distal end, form, anterior view: absent or extremely shallow, anterior surface flat between the condyles in distal view (0); groove present but shallow, expressed as a broad concave anterior margin in distal view but present as an extensive depression on the anterior surface of the femur (1); groove present and deep, expressed as a deep, U-shaped cleft in distal view and present as an extensive depression on the anterior surface of the femur (2) (Brusatte et al., 2010:290; Loewen et al., 2013:454, 455, 456; Brusatte and Carr, 2016:290). ORDERED.

367) Femur, mesiodistal crest, form, medial view: single structure (0); bifurcates distally to enclose fossa on the medial surface of the medial condyle (1) (Brusatte et al., 2010:291; Brusatte and Carr, 2016:291).

368) Femur, tibiofibular crest, size, distal view: large, width of crest is larger than half the mediolateral width of the lateral condyle (0); small, width of crest is smaller than half the mediolateral width of the lateral condyle, with the crest limited to the medial half of the lateral condyle (1) (Loewen et al., 2013:456; Brusatte and Carr, 2016:356).

TIBIA (5 characters)
369) Tibia, length relative to the femur: 1.05 or greater (0); less than 1.00 (1) (Sereno et

al., 2009:99; Brusatte et al., 2010:292; Loewen et al., 2013:459; Brusatte and Carr, 2016:292).

370) Tibia, lateral condyle of proximal end, anterior process, proximal view: absent (0); present (1) (Carr and Williamson 2010:266; Brusatte et al., 2010:293; Loewen et al., 2013:460; Brusatte and Carr, 2016:293).

371) Tibia, proximal surface, proximal extent of cnemial crest relative to the proximal extent of the posterior condyles: cnemial crest extends farther proximally than condyles (0); cnemial crest and condyles extend to the same approximate level proximally (1) (Brusatte and Carr, 2016:322).

372) Tibia, lateral malleolus, lateral extent, anterior view: limited, mediolateral measure is less than 40% of mediolateral width of adjacent shaft (0); extensive, mediolateral measure greater than 40% of mediolateral width of adjacent shaft (1) (Carr and Williamson 2010:271; Brusatte et al., 2010:294; Loewen et al., 2013:463; Brusatte and Carr, 2016:294).

373) Tibia, lateral malleolus, position relative to medial malleolus: extend to approximately the same level distally (0); lateral malleolus extends significantly further distally than medial malleolus (1) (Brusatte et al., 2010:295; Loewen et al., 2013:464; Brusatte and Carr, 2016:295).

FIBULA (1 character)
374) Fibula, iliofibularis tubercle, form, anterior view: single crest (0); large, rugose, and formed by two crests separated by a depressed fossa ("bipartite" condition) (1) (Mader and Bradley, 1989; Holtz, 2001:36; Carr and Williamson, 2010:267; Brusatte et al., 2010:296; Loewen et al., 2013:467; Brusatte and Carr, 2016:296).

ASTRAGALUS (2 characters)
375) Astragalus, ascending process, mediolateral width of base compared to width of entire bone, anterior view: half or less (0); greater than half (1) (Carpenter et al., 2005; Carr and Williamson, 2010:268; Brusatte et al., 2010:297; Loewen et al., 2013:472; Brusatte and Carr, 2016:297).

376) Astragalus, fossa on anterior surface of ascending process, form, anterior view: shallow concavity that covers most of the ventral region of the ascending process (0); deep, triangular or ovoid fossa immediately above midpoint of condyles, set within a broad fossa that covers most of the ventral region of the ascending process (1) (Carpenter et al., 2005; Carr and Williamson, 2010:269; Brusatte et al., 2010:298; Brusatte and Carr, 2016:298).

PES (10 characters)
377) Pes, arctometatarsalian condition, form of shaft of metatarsal III, anterior and posterior views: pes not arctometatarsalian, mtt III subcylindrical and its proximal end is exposed anteriorly (0), pes arctometatarsalian, mtt III wedge-shaped and its proximal end is covered anteriorly by contact between mtts II and IV (1) (Holtz, 1994; Holtz, 2001:37; Carr and Williamson 2010:271; Sereno et al., 2009:100; Brusatte et al., 2010:299; Loewen et al., 2013:489; Brusatte and Carr, 2016:299).

378) Pes, metatarsal III, form of medial surface, anterior and posterior views: straight or subtly

convex (0); with medial convex expansion forming a bulge along the distal part of the shaft (1) (Li et al., 2010:289; Brusatte et al., 2010:300; Loewen et al., 2013:488; Brusatte and Carr, 2016:300).

379) Pes, metatarsal III, ventral nonarticular surface immediately proximal to the distal condyles, form: concave (0); raised subtriangular platform (1) (Sereno et al., 2009:101; Brusatte et al., 2010:301; Loewen et al., 2013:490; Brusatte and Carr, 2016:301).

380) Pes, metatarsals II-IV, distal separation when in articulation: metatarsals closely appressed and distance between II-III and III-IV is approximately equal (0); distal ends of II and IV diverge from III, and distance between III-IV greater than that between II-III (1) (Brusatte et al., 2010:302; Loewen et al., 2013:481;, Brusatte and Carr, 2016:302).

381) Pes, metatarsal II, articular scar for metatarsal III on distal portion of lateral surface of shaft, form, lateral view: subtle (0); enlarged as a rugose fossa that occupies more than half of the proximodistal length of the shaft and expands in anteroposterior width distally (1) (Brusatte et al., 2010:303; Loewen et al., 2013:483, 484, 493, 494; Brusatte and Carr, 2016:303).

382) Pes, metatarsal II, lateral surface, shape, proximal view: flat or weakly concave (0); strongly concave (1) (Brusatte et al., 2010:304; Loewen et al., 2013:486; Brusatte and Carr, 2016:304).

383) Pes, metatarsal IV, distal end, ratio between anteroposterior long axis (measured from midpoint of condyles posteriorly to anterior surface of bone) and mediolateral width (measured at midpoint), distal view: greater than 1.40, distal surface is elongate anteroposteriorly (0); between 1.40 and 1.20 (1); less than 1.20, distal surface nearly square-shaped with nearly flat anterior surface (2) (Brusatte et al., 2010:305; Brusatte and Carr, 2016:305). ORDERED.

384) Pes, metatarsal IV, lateral muscle attachment scar, form, plantar view: absent or dorsoventrally long and narrow (0); wide oval rugosity that covers more than half the shaft (1) (Loewen et al., 2013:495; Brusatte and Carr, 2016:357).

385) Pes, proximal pedal phalanges, ratio of length to midshaft width: greater than (0) or less than (1) 3.0 (Brusatte et al., 2010:306; Loewen et al., 2013:498; Brusatte and Carr, 2016:306).

386) Pes, pedal unguals, lip overhanging proximal articular surface dorsally (on extensor surface), lateral and medial views: present (0); absent or reduced to a subtle tuber (1). (Brusatte et al., 2010:307; Loewen et al., 2013:500; Brusatte and Carr, 2016:307).

10. Unambiguously Optimized Synapomorphy List

Tyrannosauroidea: 13 (1), 15 (1), 18 (2), 30 (1), 103 (1), 132 (1), 164 (1), 209 (1), 233 (1), 257 (1), 259 (1), 282 (1), 311 (1), 324 (1), 343 (1), 345 (1), 358 (1), 361 (1), 362 (1).
Proceratosauridae: 5 (1), 16 (1), 17 (1), 49 (1), 55 (1), 157 (1), 158 (1), 234 (1), 247 (1), 331 (1).
Dilong + derived tyrannosauroids: 337 (1), 338 (1).
'Stokesosaurids' + derived tyrannosauroids: 41 (1), 45 (1), 58 (1), 109 (1), 144 (1), 240 (2), 241 (1), 257 (2), 259 (2), 287 (1), 305 (1), 307 (1), 310 (1), 323 (1), 349 (1), 356 (1), 365 (1).
'Stokesosauridae': 325 (1).
Xiongguanlong + derived tyrannosauroids: 15 (2), 39 (1), 60 (1), 141 (1), 262 (1), 273 (1), 276 (1), 280 (1), 282 (0), 327 (1), 329 (1), 336 (1), 366 (2).
(*Timurlengia* + Iren Dabasu taxon) + derived tyrannosauroids: 47 (1), 73 (2), 132 (3).
Dryptosaurus + derived tyrannosauroids: 1 (1), 322 (1).
Appalachiosaurus + derived tyrannosauroids: 372 (1).
Bistahieversor + derived tyrannosauroids: 34 (2), 69 (0), 71 (1), 185 (1), 186 (1), 251 (2), 381 (1), 386 (1).
Tyrannosauridae: 31 (1), 68 (1), 100 (1), 111 (0), 124 (1), 176 (1), 182 (1), 184 (1).
Albertosaurus: 86 (0), 96 (1), 106 (1), 114 (1), 115 (1), 147 (1), 180 (1), 210 (1), 358 (1).
Tyrannosaurinae: 14 (2), 40 (1), 54 (1), 81 (1), 123 (1), 125 (1), 131 (1), 174 (1), 177 (1), 178 (1), 188 (1), 216 (1), 283 (2).
Derived tyrannosaurines: 112 (1), 122 (1), 134 (1), 135 (2), 157 (1), 158 (1), 237 (0), 241 (2), 248 (1), 261 (1), 275 (1), 334 (1), 362 (2).
Daspletosaurus + (*Zhuchengtyrannus* + *Tyrannosaurus*): 20 (1), 24 (2), 42 (2), 59 (1), 91 (1), 93 (1), 97 (1), 384 (1).
Daspletosaurus: 43 (2), 72 (1), 89 (1), 113 (1), 130 (1), 154 (1), 173 (1), 184 (0), 219 (1), 229 (0), 261 (0).
Zhuchengtyrannus + *Tyrannosaurus*: 22 (1), 32 (1), 44 (1).
Tyrannosaurus: 23 (1), 264 (2).

11. Ontogenetic Character List

Characters, character states, and codings used to resolve the ontogeny of *Daspletosaurus horneri* (nov. sp.). Phylogenetic characters are identified by boldface type and the designation "PHYLO", and their numbers as they appear in the phylogenetic character list are included. Where applicable, the method of contingent coding of Brazeau (2011) is followed.

GENERAL FEATURES (1 character)
1. Size: small (0), large (1).

MAXILLA (52 characters)
2. Maxilla, promaxillary fenestra, exposure, lateral view: visible (0), hardly visible (1) (Russell, 1970).

3. Maxilla, promaxillary fenestra, position relative to ventral margin of the maxillary fenestra: same level (0), dorsal to ventral margin (1).

4. Maxilla, promaxillary fenestra, shape, caudolateral view: teardrop (0), lens (1).

5. Maxilla, maxillary fenestra, ventral margin, width, dorsolateral view: narrow (0), wide (1).

6. Maxilla, maxillary fenestra, caudodorsal margin, shape, lateral and medial views: gently concave (0), gently convex (1).

7. Maxilla, maxillary fenestra, rostrodorsal margin, length relative to the caudoventral margin, lateral view: same length (0), shorter than the caudoventral margin (1).

8. Maxilla, ascending ramus, antorbital fossa, subordinate pneumatic fossae, presence, lateral view: absent (0), present (1) (var. PHYLO #33).

9. Maxilla, ascending ramus, depression extends rostroventrally across the subcutaneous portion of the ascending process into the antorbital fossa a short distance ahead of the rostral base of the dorsolateral process and caudal to the maxillary flange: absent (0), present (1).

10. Maxilla, antorbital fossa, region below the antorbital fenestra, presence of foramina, lateral view: present (0), absent (1).

11. Maxilla, interfenestral strut, rostrocaudal length: greater than (0) or less than (1) 55% long axis of maxillary fenestra (var. PHYLO #31).

12. Maxilla, lateral interfenestral strut, fossa in base, depth, medial view: shallow (0), deep (1) (Carr, 1999).

13. Maxilla, horizontal ramus (excluding antorbital fossa), depth (measured vertically from the rostroventral corner of the antorbital fossa to the ventral margin of the lateral alveolar process) and taking its ratio relative to the length of the tooth row), lateral view: shallow,

where the ratio is 26% or less (0); deep, where the ratio is greater than 26% (1) (var. PHYLO #34).

14. Maxilla, dorsolateral process, coverage by antorbital fossa, lateral view: covered by fossa (0), more than ventral half of process covered by antorbital fossa (1) (var. PHYLO #37 (reversed)).

15. Maxilla, dorsolateral process, depth, lateral view: shallow, where it is half as deep or less as the fossa below it (0); deep, where it is greater than half as deep as the fossa below it (1).

16. Maxilla, joint surface for the palatine, rostral extent, medial view: stops caudal to the medial interfenestral strut (0), extends below the caudal edge of the strut (1).

17. Maxilla, nasomaxillary suture: tongue in groove (0), peg-in-socket (1) (PHYLO #42).

18. Maxilla, lateral surface below the antorbital fossa, series of fossae, presence, lateral view: absent (0), present (1) (PHYLO #44).

19. Maxilla, interdental notches, depth, lateral view: indistinct (0), distinct (1).

20. Maxilla, alveolar skirts, presence, lateral view: absent (0), present (1) (Carr, 1999).

21. Maxilla, alveolar foramina and sulci, size and depth: foramina and sulci of any amount that are small and shallow (0), all are large and deep (1) (Carr, 1999).

22. Maxilla, caudalmost alveolar foramen, shape, lateral view: long and elliptical (0), enlarged and round (1).

23. Maxilla, caudalmost alveolar foramen, sulcus breaches ventral edge, lateral view: does not breach and it fades caudally (0), breaches (1) (Carr, 1999).

24. Maxilla, caudalmost alveolar foramen, dorsoventral position, lateral view: close to dorsal edge (0), close to ventral edge (1).

25. Maxilla, antorbital fossa-subcutaneous junction rostral to the jugal contact, width, lateral and dorsal views: extends laterally like a shelf (0), nearly vertical, extends lateroventrally (1).

26. Antorbital fossa, lateral flange of maxilla obscuring cranialmost portion of aofo, lateral view: present as small strut (0), large strut that extends caudodorsally along the fossa (1) (PHYLO #20).

27. Maxilla, antorbital fossa, neurovascular foramina, presence, lateral view: absent (0), present (1).

28. Maxilla, number of teeth rostral to the antorbital fossa, lateral view: five (0), more than five (1).

29. Maxilla, number of tooth positions caudal to the medial interfenestral strut, medial view: 7 (0), 6 (1).

30. Maxilla, tooth #8, position relative to the maxillary fenestra, lateral view A: caudal half (0), midlength or further rostral (1).

31. Maxilla, tooth #8, position relative to the maxillary fenestra, lateral view B: caudal half or midlength (0), further rostral than midlength (1).

32. Maxilla, region of the subnarial foramen and vestibular bulla, inflation, lateral and rostral views: not expanded or expanded by the rostrolateral surface is not modified into a strut (0), expanded such that the rostrolateral surface is expanded into a strut (1) (Carr, 1999).

33. Maxilla, region rostrodorsal to the antorbital fossa, inflation, lateral view: not inflated (0), inflated (1).

34. Maxilla, region of lateral joint surface for the nasal rostral to the maxillary flange, lateral view: lateral surface is concave (0); the surface is convex (1).

35. Maxilla, notch ahead of maxillary flange, depth, lateral view: shallow (0), deep (1).

36. Maxilla, strut along rostrodorsolateral edge of bone, from premaxillary buttress to the rostral end of the maxillary flange, size, lateral view A: present, but not fully inflated and it does not extend past the caudal end of the bony naris, or extends to the maxillary flange but is moderately inflated (0); extends to the maxillary flange and is fully inflated (1).

37. Maxilla, strut along rostrodorsolateral edge of bone, from premaxillary buttress to the rostral end of the maxillary flange, size, lateral view B: present, but not fully inflated and it does not extend past the caudal end of the bony naris (0); extends to the maxillary flange but is moderately inflated, or it extends to the maxillary flange and is fully inflated (1).

38. Maxilla, antorbital fenestra, rostralmost incursion, position relative to the height of the maxillary fenestra: at the level of the dorsal margin (0), above the midheight of the fenestra (1).

39. Maxilla, medial interfenestral strut, form, medial view: basal region extends caudodorsally (0), entire strut extends rostrodorsally (1).

40. Maxilla, medial interfenestral strut, position of base, medial view: at the rostral half of the base of the lateral interfenestral strut (0), at the midlength or caudal half of the base of the lateral strut (1).

41. Maxilla, medial interfenestral strut, position of dorsal half relative to the rostral margin of the antorbital fenestra, medial view: caudal to margin of fenestra (i.e., visible in lateral view) (0), rostral to margin of fenestra (not visible laterally) (1).

42. Maxilla, caudal antromaxillary fenestra, position, caudal view: not close to the anteroventral margin of the antorbital fenestra (0), close to the anteroventral margin (1) (Brochu, 2003).

43. Maxilla, epiantral recess, depth, medial view: shallow (0), deep (1) (Carr, 1999).

44. Maxilla, maxillary antrum, dorsal margin, orientation, medial view: extends horizontally (0), extends rostroventrally at a steep angle (1) (Carr, 1999).

45. Maxilla, maxilla, palatal process, shape, medial view: straight (0), sigmoid and slopes rostrodorsally (1) (PHYLO #51).

46. Maxilla, intermaxillary process, joint surface, ventral view: confluent with medial surface of palatal process, process has a uniform width (0), caudal end extends medially, widens rostral end of palatal process (1).

47. Maxilla, palatal shelf, tooth root bulges caudal to maxillary antrum, size, medial view: indistinct (0), distinct (1) (Carr, 1999).

48. Maxilla, palatal process, width next to last alveolus: narrower than alveolus (0), as wide as the alveolus (1).

49. Maxilla, palatal process, margin of the choana, extent: at the level of alveoli 4-8 (0), 7-10 (1) (Currie, 2003).

50. Maxilla, depressions for teeth below rostral end of palatal process, medial view: indistinct (0), distinct (1).

51. Maxilla, depressions for teeth below caudal end of palatal process, medial view: indistinct (0), distinct (1).

52. Maxilla, interdental plates, dorsoventral position, medial view: far dorsal to the ventral margin of the bone (half their height or more above the ventral margin) (0), close to the ventral margin (less than half their height above the ventral margin) (1).

53. Maxilla, ventral jugal process, depth, lateral view: shallow (0), deep (1).

NASALS (9 characters)
54. Nasals, rostral region, width and height: narrow and deep (0), wide and low (1).

55. Nasals, dorsal surface, texture: relatively smooth (0), distinctly coarse (1) (PHYLO #56).

56. Nasals, rugosities, height, lateral view: tall (0), low (1) (var. PHYLO #57).

57. Nasals, caudolateral process, presence, lateral view: absent (0), present (1) (PHYLO #61).

58. Nasals, dorsal foramina rows, mediolateral position, dorsal view: lateral (0), medial (1).

59. Nasals, dorsal foramina rows, point of convergence into a single row, lateral view: above interfenestral strut (0), ahead of the maxillary fenestra (1).

60. Nasals, dorsal foramina rows, concentration of foramina rostrally: close together (0), not close together (1).

61. Nasals, frontal ramus, rostral half of dorsum, form, dorsal view: flat or concave (0), convex (1) (Carr, 1999).

62. Nasals, frontal ramus, concavity at rostral end, depth: concavity is absent or deep (0), very deep (1) (Carr, 1999).

LACRIMAL (16 characters)
63. Lacrimal, shape of the bone, lateral view: T-shaped (0), 7-shaped (1) (PHYLO #65).

64. Lacrimal, cornual process, differentiation, lateral view: not differentiated from the dorsal surface of the rostral ramus (0), differentiated from the rostral ramus (1) (var. PHYLO #67, 68).

65. Lacrimal, cornual process, convex surface, caudal extent, dorsal view: does not extend onto dorsum of supraorbital ramus (0), extends onto supraorbital ramus (1).

66. Lacrimal, accessory cornual process, form, lateral view: absent, or long and dorsoventrally shallow (0); short and deep (1).

67. Lacrimal, antorbital fossa, margin along the leading edge of the ventral ramus, undercut, presence, lateral and rostrolateral views: edge is undercut by the fossa (0), edge is not undercut and the surfaces are confluent (1) (PHYLO #77 (reversed).

68. Lacrimal, dorsal ramus, widest region, dorsal view: above caudal end of ventral ramus (0), behind caudal edge of ventral ramus (1).

69. Lacrimal, surface rostrodorsal to the lacrimal pneumatic recess, form, lateral view: concave (0), convex (1) (Carr, 1999).

70. Lacrimal, strut that bounds the pneumatic recess rostrally, position relative to the lateral surface of the bone, lateral view: inset from the lateral surface (0), nearly level with the lateral surface (1).

71. Lacrimal, proximal accessory recess, enclosure, lateroventral view: recess is open and long (0); recess is enclosed from behind, reducing it to a small and rostrally positioned opening (1).

72. Lacrimal, strut that extends between the ventral ramus and rostral ramus, inflation, lateral view: not inflated such that it is positioned medial to the external surface of the ventral ramus and dorsal part of the rostral ramus (0), inflated such that it is nearly level with the external surface of each ramus (1).

73. Lacrimal, antorbital fossa, margin along the dorsal edge of the strut that bounds the lacrimal pneumatic recess rostrally: the fossa and the subcutaneous surface do not merge together (0), the surfaces are merged together (1).

74. Lacrimal, marginal bone, presence, lateral and medial views: absent or does not form a distinct flange that extends into the antorbital fenestra (0), forms a distinct flange (1).

75. Lacrimal, joint surface for the nasal, medial view: does not extend laterally across rostral ramus immediately ahead of the ventral ramus (0); extends laterally across the rostral ramus immediately ahead of the ventral ramus (1).

76. Lacrimal, proximal region of the ventral ramus, fossa, presence, lateral view: present (0), absent (1).

77. Lacrimal, joint surface for the prefrontal, form, medial view: stepped (gently peg in socket) (0); deeply peg in socket (1).

78. Lacrimal, orbitonasal ridge, inflation: not inflated (0), inflated (1).

JUGAL (5 characters)
79. Jugal, pneumatic recess, lateral margin, direction, lateral view: extends caudodorsally at a low angle (0), extends caudodorsally at a steep angle (1) (PHYLO #93).

80. Jugal, joint surface for the postorbital, form, lateral view: shallow interlocking notch (0), deep interlocking notch with a distinct rostrodorsally extending flange (1) (PHYLO #98).

81. Jugal, cornual process, prominence and texture, lateral view: not maximally prominent, where the caudal margin of the process is not differentiated from the ventral margin of the bone, smooth or lightly textured (0); maximally prominent, where the caudal margin of the process is separated by a change in orientation from the ventral margin of the bone, coarsely textured (1) (PHYLO #103).

82. Jugal, body of the bone, flange ahead of the cornual process, distinctiveness, lateral view: present and distinct (0), eliminated by inflation of the bone (1).

83. Jugal, ventral quadratojugal process, orientation, lateral view: caudodorsal (0), horizontal (1).

POSTORBITAL (1 character)
84. Postorbital, cornual process, position relative to laterotemporal fenestra, lateral view: does not approach fenestra (0), closely approaches the fenestra (1) (PHYLO #113).

SQUAMOSAL (2 characters)
85. Squamosal, dorsal postorbital process, form, lateral view: gently convex or concave along its caudal extent (0), concave for the entire extent of the process (1).

86. Squamosal, caudal process, fossa between the rostroventral corner of the process and the base of the quadratojugal process, presence, lateral view: absent (0), present (1).

QUADRATOJUGAL (2 characters)
87. Quadratojugal, shaft, foramen, presence, lateral view: absent (0), present (1) (PHYLO #140).

88. Quadratojugal, notch between the joint surface for the quadrate and the proximal end of the ventral quadrate process, presence, lateral view: absent (0), present (1).

ECTOPTERYGOID (24 characters)
89. Ectopterygoid, jugal ramus, depth to length ratio: less than 70% (0), 70% or greater (1) (PHYLO #176).

90. Ectopterygoid, jugal ramus, ridge along the lower half of the process, prominence, caudal view: distinct (0), low (1).

91. Ectopterygoid, jugal ramus, rostrodorsolateral edge, form, rostral view: evenly convex (0), developed into a massive ridge that produces a shallow fossa on the dorsal surface of the process (1).

92. Ectopterygoid, jugal ramus, rostroventral edge, form, rostral view: massive ridge (0), ridge is low and indistinct (1).

93. Ectopterygoid, jugal ramus, ventral surface, form, ventral view: clear distinction between convex rostral region and caudal region that is concave or convex (0), distinction obliterated by inflation of the process (1).

94. Ectopterygoid, jugal ramus, rostral edge, form: concavity in the ramus separates it from the body of the bone (0), the leading edge of the ramus is straight and the body extends rostromedially from it (1).

95. Ectopterygoid, strut at medial half of the caudal edge of the recess, size: low (0), prominent (1).

96. Ectopterygoid, joint surface for the jugal, flange, ventral depth: prominent (0), not prominent (1).

97. Ectopterygoid, groove between jugal ramus and ventral flange at the joint surface for the jugal, presence, lateral view: groove is present (0), groove is absent (1).

98. Ectopterygoid, ectopterygoid pneumatic recess, width to length ratio: greater than 35% (0), 35% or less (1) (PHYLO #177, reversed).

99. Ectopterygoid, strut at medial half of the caudal edge of the recess, size: low (0), prominent (1) (PHYLO #178)

100. Ectopterygoid, fossa rostral to pneumatic recess, distinctiveness, ventral view: deep and distinct (0), shallow (1).

101. Ectopterygoid, pneumatic fossa on the caudolateral process, proximal end, form, ventral view: concave (0), convex (1).

102. Ectopterygoid, pneumatic fossa on the caudolateral process, medial surface, form: not developed into a massive strut (0), developed into a massive strut (1).

103. Ectopterygoid, lateral fossa, form, dorsal view: concave (0), convex (1).

104. Ectopterygoid, crease between the bulbous ventral convexity of the body and the caudal process, depth, ventral view: deep (0), shallow (1).

105. Ectopterygoid, body, ventral convexity, form as it extends laterally toward the jugal process, distinctiveness, ventral view: ridge is indistinct (0), ridge is distinct (1).

106. Ectopterygoid, body, ventral convexity, ridge that extends laterally toward the jugal process, direction, ventral view: medial (0), rostrolateral (1).

107. Ectopterygoid, caudal process, rostrolateral surface, muscle attachment, form, caudal view: gently convex (0), dorsoventrally deep and convex (1).

108. Ectopterygoid, caudal process, caudomedial surface, dorsal groove, form: dorsoventrally shallow but deeply incised slot (0), deep groove (1).

109. Ectopterygoid, caudal process, caudomedial surface, ventral ridge and groove, form: both structures are present (0), caudal process is inflated and has merged the ridge and fossa into a single convex surface (1).

110. Ectopterygoid, caudal process, region of the fossa between the ventral surface of the bone and the ridge, width: narrow (0), wide (1).

111. Ectopterygoid, muscle scar of caudal process, lateral extent: extend onto the caudal surface of the jugal process (0), stops short of the jugal process (1).

112. Ectopterygoid, muscle scar of caudal process, width, ventral view: does not span the width of the process (0), spans the width of the process (1).

PALATINE (2 characters)

113. Palatine, dorsal ramus, orientation, lateral view: rostrodorsal (0), dorsal (1) (PHYLO #180).

114. Palatine, caudal pneumatic recess, rostrocaudal position relative to the caudal margin of the dorsal ramus, lateral view: below margin (0), rostral to margin (1) (PHYLO #183).

DENTARY (19 characters)
115. Dentary, symphysis, leading edge, form, medial view A: pair of vertical ridges (0), interlocking (1) (PHYLO #231).

116. Dentary, symphysis, leading edge, form, medial view B: not interlocking or interlocking surface is limited to the ventral half of the symphysis (0), entire height of the symphysis is interlocking (1).

117. Dentary, symphysis, rostroventral notch, presence, medial view: absent (0), present (1).

118. Dentary, joint surface for the rostral process of the splenial, texture, medial view: smooth (0), coarsened by low rostroventrally extending ridges (1) (PHYLO #232).

119. Dentary, bone form, dorsal view: twisted axially (0), not twisted (1).

120. Dentary, bone form, lateral bowing, dorsal view: distinct (0), indistinct (1).

121. Dentary, bone, form, lateral surface, lateral view: strongly convex such that the ventral row of foramina extends along the ventral surface of the bone (0), not strongly convex where the foramina extend along the ventrolateral surface of the bone (1).

122. Dentary, bone, lateral surface, texture, lateral view: dominated by immature bone grain (0), not dominated by immature bone grain (1).

123. Dentary, Meckelian groove, depth, medial view: shallows and fades as it approaches the Meckelian foramen (0), deep condition extends to the Meckelian foramen (1) (var. PHYLO #236).

124. Dentary, Meckelian groove, form, medial view: pinched out into a crease rostrally (0), does not become pinched out (1).

125. Dentary, medial surface, fossa rostrodorsal to the Meckelian foramen and caudal to the symphysis, depth, medial view: shallow (0), deep (1).

126. Dentary, ventral bar, form, medial view: strongly convex (0), convex rostrally and flat caudally (1).

127. Dentary, ventral bar, rostral end, swelling, presence, medial view: indistinct swelling (0), rugose mound (1) (PHYLO #238).

128. Dentary, lingual bar, joint surface for the intercoronoid, distinctiveness, medial view: fades out between alveoli 6-10 (0), distinct along its entire course (1).

129. Dentary, lingual bar, rostrodorsal crease, depth: distinct (0), shallow (1).

130. Dentary, caudal plate, contours, lateral view: lateral surface is convex and continuous with the dentigerous region (0); the convex surface of the dentigerous region splits caudally and extends along the ventral and dorsal margins of the caudal plate, which is concave between the convexities (1).

131. Dentary, Meckelian fossa, rostral limit, medial view A: at the level of the 13th alveolus (0), greater than alveolus 13 (1).

132. Dentary, Meckelian fossa, rostral limit, medial view B: at the level of the 14th alveolus or less (0), greater than alveolus 14 (1).

133. Dentary, bone depth, lateral view: shallow (0), deep (1).

SURANGULAR (26 characters)
134. Surangular, bone depth, lateral view: shallow or intermediate, less than 175 mm deep (0); deep, 175 mm deep or greater (1).

135. Surangular, caudal surangular foramen, distance below the surangular shelf, lateral view: far ventral (0), close (1) (PHYLO #240).

136. Surangular, recess caudal to the caudal surangular foramen, depth, lateral view: shallow or intermediate in depth (0), deep (1).

137. Surangular, shelf lateroventral to the caudal surangular foramen, rostral extent, lateral view: along the caudal extent of the foramen or to the rostroventral corner of the foramen (0), extends rostral to the foramen (1).

138. Surangular, shelf lateroventral to the caudal surangular foramen, dorsoventral height relative to the foramen, lateral view: extends above the level of the ventral margin of the foramen (0), shelf is situated ventral to the level of the foramen (1).

139. Surangular, fossa above the caudal surangular foramen, presence, ventrolateral view: absent (0), present (1).

140. Surangular, fossa rostral to the caudal surangular foramen, depth, lateral view: shallow (0), deep (1).

141. Surangular, ridge ahead of the fossa that is rostral to the caudal surangular foramen, form, lateral view: a low swelling (0), a distinct ridge (1).

142. Surangular, surangular shelf, lateral orientation, lateral view: extends directly laterally from the bone (0), extends lateroventrally (1) (PHYLO #240).

143. Surangular, surangular shelf, rostral extent, lateral view: fades a short distance rostral to the caudal surangular foramen (0), extends far rostral to the foramen (1).

144. Surangular, surangular shelf, region of greatest width, dorsal view: rostral end of the caudal surangular foramen (0), rostral to the foramen (1).

145. Surangular, caudal region of the surangular shelf below the glenoid region, groove in lateral or ventrolateral surface, lateral view: absent or indistinct (0), distinct (1).

146. Surangular, coronoid region, rostral extent, form, dorsal view: gently concave without an abrupt plane change to the surangular shelf (0), flat with an abrupt plane change that separates it from the surangular shelf (1).

147. Surangular, coronoid region, regionalization, dorsal view: distinctly coarse rostromedial region is present (0), regionalization is absent (1).

148. Surangular, coronoid region ahead of the glenoid fossa, form, dorsal view: flat (0), deeply concave (1).

149. Surangular, coronoid process, medial surface, texture: finely textured (0), coarsely textured (1).

150. Surangular, lateral part of the glenoid, rostrodorsal angle, dorsal view: steep (0), low (1).

151. Surangular, lateral part of the glenoid, rostrolateral quadrant, form: concave (0), convex (1).

152. Surangular, quadrate notch, depth, lateral view: deep, 49% of its length or higher (0); shallow, less than 40% of its length (1).

153. Surangular, glenoid process, pit, depth: absent or shallow (0), deep (1).

154. Surangular, glenoid tubercle, height, lateral view: lower than the caudal flange of the articular or the same height (0), taller than the caudal process (1).

155. Surangular, lateral scar, extent on the caudal glenoid process: covers its rostral half (0), covers the entire process (1).

156. Surangular, bar that gives rise to the medial flange, depth, medial view: deep (0), shallow (1).

157. Surangular, joint surface for the angular, orientation of the caudodorsal extent of the margin, lateral view: rostrodorsal (0), rostroventral (1).

158. Surangular, joint surface for the angular, slot above its caudoventral edge: present (0), absent (1).

159. Surangular, joint surface for the angular, texture, lateral view: coarse (0), coarsened by massive ridges (1).

ANGULAR (1 character)
160. Angular, bone, form, lateral view: not flexed (0), flexed (1) (PHYLO #247).

SPLENIAL (3 characters)
161. Splenial, caudal process, ridge on medial surface: limited caudally (0), massive and extends rostrally (1).

162. Splenial, bar below the rostral mylohyoid foramen, dorsoventral thickness: shallow (0), deep (1).

163. Splenial, fossa along dorsal margin of the rostral mylohyoid foramen, presence, medial view: present (0); absent where the corresponding surface is convex (1).

DENTITION
164. Dentition, maxillary, number of teeth: 15 (0), greater than 15 (1).

12. Unambiguously Optimized Synontomorphies List

Growth stage 1 (exemplar: MOR 553S): n/a
Growth stage 2 (exemplar: AMNH FARB 5477): 1 (1).
Growth stage 3 (exemplar: MOR 590): 2 (1), 6 (1), 8 (1), 11 (1), 14 (1), 19 (1), 20 (1), 21 (1), 22 (1), 28 (1), 30 (1), 33 (1), 34 (1), 37 (1), 38 (1), 40 (1), 42 (1), 43 (1), 45 (1), 49 (1), 50 (1), 52 (1), 53 (1), 164 (1).
Growth stage 4 (exemplar: MOR 3068): 135 (1), 136 (1), 141 (1), 144 (1), 145 (1), 147 (1), 148 (1), 153 (1), 155 (1), 156 (1), 158 (1), 161 (1).
Growth stage 5 (exemplar: MOR 1130): 134 (1), 137 (1), 138 (1), 142 (1), 143 (1), 146 (1), 150 (1), 151 (1), 152 (1), 157 (1), 159 (1), 162 (1), 163 (1).

13. References

Bakker, R.T., M. Williams, and P.J. Currie. 1988. *Nanotyrannus*, a new genus of pygmy tyrannosaur, from the latest Cretaceous of Montana. Hunteria 1: 1-30.

Benson, R.B.J. 2008. New information on *Stokesosaurus*, a tyrannosauroid (Dinosauria: Theropoda) from North America and the United Kingdom. Journal of Vertebrate Paleontology 28: 732-750.

Brazaitis, P & and M. E. Watanabe. Crocodilian behavior: a window to dinosaur behavior? *Historical Biology* **23**, 73-90 (2011).

Brazeau, M. D. Problematic character coding methods in morphology and their effects. *Biol. J. Linn. Soc.* **104**, 489-498 (2011).

Brochu, C.A. 2003. Osteology of *Tyrannosaurus rex*: insights from a nearly complete skeleton and high-resolution computed tomographic analysis of the skull. Society of Vertebrate Paleontology Memoir 7:1-138.

Brusatte, S.L. and R.B.J. Benson. 2013. The systematics of Late Jurassic tyrannosauroids (Dinosauria: Theropoda) from Europe and North America. Acta Palaeontologica Polonica 58: 47-54.

Brusatte, S.L., M.A. Norell, T.D. Carr, G.M. Erickson, J.R. Hutchinson, A.M. Balanoff, G.S. Bever, J.N. Choiniere, P.J. Makovicky, and X. Xu. 2010. Tyrannosaur paleobiology: new research on ancient exemplar organisms. Science 329: 1481-1485.

Brusatte, S.L. and T.D Carr. 2016. The phylogeny and evolutionary history of tyrannosauroid dinosaurs. Scientific Reports 6: 20252, doi: 10.1038/srep20252.

Brusatte, S.L., A. Averianov, H.-D. Sues, and I.B. Butler. 2016. New tyrannosaur from the mid-Cretaceous of Uzbekistan clarifies the evolution of giant body sizes and advanced senses in tyrant dinosaurs. Proceedings of the National Acadamy of Sciences USA 113(13): 3447, doi: 10.1073/0nas.1600140113.

Carpenter, K., C. Miles, and K. Cloward. 2005. New small theropod from the Upper Jurassic Morrison Formation of Wyoming. *In* K. Carpenter (editor), The Carnivorous Dinosaurs: 23-48. Bloomington: University of Indiana Press.

Carr, T.D. 1999. Craniofacial ontogeny in Tyrannosauridae (Dinosauria: Coelurosauria). Journal of Vertebrate Paleontology 19: 497-520.

Carr, T.D., T.E. Williamson, and D.R. Schwimmer. 2005. A new genus and species of tyrannosauroid from the Late Cretaceous (Middle Campanian) Demopolis Formation of Alabama. Journal of Vertebrate Paleontology 25: 119-143.

Carr, T.D. and T.E. Williamson. 2010. *Bistahieversor sealeyi*, gen. et sp. nov., a new tyrannosauroid from New Mexico and the origin of deep snouts in Tyrannosauroidea. Journal of Vertebrate Paleontology 30: 1-16.

Currie, P.J. 2003. Cranial anatomy of tyrannosaurid dinosaurs from the late Cretaceous Alberta, Canada. Acta Paleontological Polonica 48: 191-226.

Currie, P.J., J.H. Hurum, and K. Sabath. 2003. Skull structure and evolution in tyrannosaurid dinosaurs. Acta Palaeontologica Polonica 48: 227-234.

Gold, M.E.L., S.L. Brusatte, and M.A. Norell. 2013. The cranial pneumatic sinuses of the tyrannosaurid *Alioramus* (Dinosauria: Theropoda) and the evolution of cranial pneumaticity in theropod dinosaurs. American Museum Novitates 3790: 1-46.

Goloboff, P. A., Farris, J. A. & Nixon, K. C. 2008. TNT, a free program for phylogenetic analysis. Cladistics 24: 774-786.

Hieronymus, T. L., Witmer, L. M., Tanke, D. H. & Currie, P. J. 2009. The facial integument of centrosaurine ceratopsids: morphological and histological correlates of novel skin structures. Anat. Record 292: 1370-1396.

Hieronymus, T. L. & Witmer, L. M. 2010. Homology and evolution of avian compound rhamphothecae. Auk 127: 590-604.

Holtz, T. R. 1994. The phylogenetic position of the Tyrannosauridae: Implications for theropod systematics. Journal of Paleontology 68: 1100-1117.

Holtz, T.R. 2001. The phylogeny and taxonomy of the Tyrannosauridae. *In* D.H. Tanke and K. Carpenter (editors), Mesozoic Vertebrate Life: 64-83. Bloomington: Indiana University Press.

Li, D., M.A. Norell, K. Gao, N.D. Smith, and P.J. Makovicky. 2010. A longirostrine tyrannosauroid from the Early Cretaceous of China. Proceedings of the Royal Society Series B, 277: 183-190.

Kurzanov, S.M. 1976. A new Late Cretaceous carnosaur from Nogon-Tsav Mongolia [in Russian]. Soviet-Mongolian Paleontological Expeditions 3: 93-104 (1976).

Loewen, M. A., Irmis, R. B., Sertich, J. J. W., Currie, P. J. & Sampson, S. D. Tyrant dinosaur evolution tracks the rise and fall of Late Cretaceous oceans. PLoS ONE 8(11), e79420, doi: 10.1371/journal.pone.0079420 (2013).

Lü, J., L. Yi, S.L. Brusatte, L. Yang, H. Li, and L. Chen. 2014. A new clade of Asian Late Cretaceous long-snouted tyrannosaurids. Nature Communications 5: 3788 (DOI: 10.1038/ncomms4788).

Mader, B.J. and R.L. Bradley. 1989. A redescription and revised diagnosis of the syntypes of the Mongolian tyrannosaur *Alectrosaurus olseni*. Journal of Vertebrate Paleontology 9: 41-55.

Maleev, E. A. 1974. Gigantic carnosaurs of the family Tyrannosauridae. Joint Soviet-Mongolian Paleontological Expedition, Transactions, 1: 132-191. (In Russian.)

Molnar, R.E. 1974. A distinctive theropod dinosaur from the Upper Cretaceous of Baja California (Mexico). Journal of Paleontology 48: 1009-1017.

Molnar, R.E. 1991. The cranial morphology of *Tyrannosaurus rex*. Palaeontographica Abteilung A 217: 137-176.

H. F. Osborn, H.F. 1912/. The crania of *Tyrannosaurus* and *Allosaurus*. Memiors of the American Museum of Natural History 1: 1-30.

Papp, M.J. 2000. A Critical Appraisal of Buccal Soft-Tissue Anatomy in Ornithischian Dinosaurs. Unpublished MSc Dissertation, Ohio University, Athens, 229 pp.

Rauhut, O.W.M., A.C. Milner, and S. Moore-Fay. 2010. Cranial osteology and phylogenetic position of the theropod dinosaurs *Proceratosaurus bradleyi* (Woodward, 1910) from the Middle Jurassic of England. Zoological Journal of the Linnean Society 158: 155-195.
Russell, D.A. Tyrannosaurs from the Late Cretaceous of western Canada. National Museum of Natural Sciences Publications in Palaeontology 1: 1-32 (1970).

Sedlmayr, J.C. 2002. Anatomy, Evolution, and Functional Significance of Cephalic Vasculature in Archosauria. Unpublished PhD Dissertation, Ohio University, Athens, 398 pp.

Sereno, P.C., A.L. Beck, D.B. Duthiel, B. Gado, H.C.E. Larsson, G.H. Lyon, J.D. Marcot, O.W.M. Rauhut, R.W. Sadlier, C.A. Sidor, D.J. Varricchio, G.P. Wilson, J.A. Wilson. 1998. A long-snouted predatory dinosaur from Africa and the evolution of spinosaurids. Science 282: 1298-1302.

Sereno, P.C., L. Tan, S.L. Brusatte, H.J. Kriegstein, X. Zhao, and K. Cloward. 2009. Tyrannosaurid skeletal design first evolved at small body size. Science 326: 418-422.

Witmer, L.M. 1997. The evolution of the antorbital cavity of archosaurs: a study in soft-tissue reconstruction in the fossil record with an analysis of the function of pneumaticity. Society of Vertebrate Paleontology Memoir 3: 1-173.

Witmer, L.M. and R.C. Ridgley. 2009. New insights into the brain, braincase and ear region of tyrannosaurs (Dinosauria, Theropoda) with implications for sensory organization and behavior. Anatomical Record 292: 1266-1296.

13. Data S1. Ontogenetic Specimen-Character Matrix.

```
                           111111111122222222223333333333444444444455555555556666666666777
Taxon/Node        123456789012345678901234567890123456789012345678901234567890123456789012
                  --------------------------------------------------------------------------------------------
artificial embryo 000000000000000000000000000000000000000000000000000000000000000000000000
MOR 553S          0???????????????????????????????????????????????????????????????????????
AMNH FARB 5477    100000001?00?000010000?0100000?000??00?0?000000?00000???????????????????
MOR 590           1100010110110100?01111000011010011001101011010?0110110100000000000000000
MOR 3068          1???????????????????????????????????????????????????????????????????????
MOR 1130          11111111011?1111111111111011111111111111111111111111111011111111111111111
```

Input data matrix (continued):

```
                                                 111111111111111111111111111111111111111111111
                  777777788888888889999999999000000000011111111112222222222333333333344444
Taxon/Node        345678901234567890123456789012345678901234567890123456789012345678901234
                  --------------------------------------------------------------------------------------------
artificial embryo 000000000000000000000000000000000000000000000000000000000000000000000000
MOR 553S          ??????????????????????????????????????????0?000000000000000?0???????????
AMNH FARB 5477    ????????????????????????????????????????????????????????????????????????
MOR 590           000000000000000000100110111111111?110110111011111101111101101000000100000
MOR 3068          ?????????????????????????????????????????????????????????????011000?1001
MOR 1130          111111111111111111111111111111111111111100111111111111111111111111111111
```

Input data matrix (continued):

```
                  11111111111111111111
                  44444555555555566666
Taxon/Node        56789012345678901234
                  --------------------------------------
artificial embryo 00000000000000000000
MOR 553S          ????????????????????
AMNH FARB 5477    ???????????????????0
MOR 590           00000000000000000001
MOR 3068          101110001111010?100?
MOR 1130          111101111?1111111111
```

15. Table S1. Measurements of the skull and craniomandibular bones of the type (MOR 590) and paratype specimen (MOR 1130) of *Daspletosaurus horneri* sp. nov. All measurements are in millimeters. Abbreviations: L, left; R, right.

Measurement	MOR 590	MOR 1130
Skull, rostrocaudal length (premaxilla-quadrate)	894.5 [L]	947.0+
Skull, dorsoventral height through postorbital bar	363.4 [L]	-
Nasal, rostrocaudal length	440.3+	
Nasal, caudal end of bony naris, mediolateral width	-	121.2+
Nasal, midregion, minimum mediolateral width	~67.9	71.6
Nasal, frontal ramus, minimum mediolateral width	-	~52.2
Maxilla, maximum rostrocaudal length	~585.6 [L]	521.5 [L]
Maxilla, dorsoventral height, rostral end of internal antorbital fenestra	275.4+ [L]	280.2 [R]
Maxilla, antorbital fossa, length rostral to internal antorbital fenestra	149.8 [L]	136.1 [L], 129.5 [R]
Maxilla, length ahead of the internal antorbital fenestra	347.2 [L]	364.3 [L], 333.4 [R]
Maxilla, tooth row, maximum rostrocaudal length	467.0 [L]	521.5 [L], ~477.0 [R]
Lacrimal, rostral ramus, rostrocaudal length	149.0 [L]	168.9 [L]
Lacrimal, ventral process, dorsoventral height	195.4+ [L]	165.7+ [R]
Lacrimal, supraorbital process, rostrocaudal length	18.9 [R]	26.9 [L]
Lacrimal, dorsal ramus (rostral ramus + supraorbital ramus), rostrocaudal length	230.7+ [L]	300.9 [L]
Lacrimal, maximum dorsoventral height (dorsal ramus + ventral ramus)	270.1 [L]	245.9+ [R]
Jugal, maximum rostrocaudal length	405.0 [L]	366.9+ [R]
Jugal, maxillary ramus, rostrocaudal length	~196.9 [L]	135.7+ [R]
Jugal, maxillary ramus, maximum dorsoventral height	100.5 [L]	79.9+
Jugal, quadratojugal ramus, maximum length	103.2+ [L]	62.5+ [R]
Jugal, quadratojugal ramus, dorsoventral height at it rostral end	51.0 [L]	61.2 [R]
Jugal, postorbital process, dorsal height	163.6+ [L]	108.7+ [R]
Postorbital, maximum dorsal rostrocaudal length	232.0+ [L]	-
Postorbital, frontal process, maximum rostrocaudal length	~98.6 [L]	-
Postorbital, squamosal process, maximum rostrocaudal length	~101.8 [L]	-
Postorbital, jugal process, dorsoventral height	~191.7 [L]	-
Postorbital, maximum height through entire bone	~236.4 [L]	-
Squamosal, maximum length from the postorbital ramus to the caudal process	~215.3 [L]	172.3+ [R]
Squamosal, quadratojugal process, maximum rostrocaudal length	85.9 [L]	42.6+ [R]
Squamosal, postorbital ramus, dorsoventral height at midlength	57.8 [L]	-
Squamosal, length from tip of medial process to rostral edge of quadrate cotyle	175.7 [L]	145.4+ [R]
Quadratojugal, bone, maximum dorsoventral height	151.4 [L]	198.7+ [R]
Quadratojugal, squamosal process, maximum rostrocaudal length	81.6+ [L]	127.2 [R]
Quadratojugal, stem, minimum length	25.3+ [L]	36.8 [R]
Quadratojugal, maximum ventral length (jugal + ventral quadrate process)	149.5 [L]	176.0 [R]
Quadrate, bone, maximum dorsoventral height	194.1+ [L]	~262.2 [R]
Quadrate, maximum rostrocaudal length through orbital process	-	~160.5 [R]
Quadrate, maximum mediolateral width across mandibular condyles	99.3 [L]	109.4 [R]
Vomer, maximum rostrocaudal length	329.3+	399.2+
Vomer, rostral process, maximum mediolateral width	46.5	56.7+
Palatine, bone, rostrocaudal length (maxillary process + dorsolateral process)	204.7+ [L]	229.9+ [R]
Palatine, bone, rostrocaudal length (maxillary process + pterygoid process)	227.3+ [L]	234.0+ [R]

Palatine, maximum dorsoventral height	166.2 [L], 159.1 [R]	156.8 [L]
Ectopterygoid, bone, maximum rostrocaudal length	-	~88.1 [R]
Ectopterygoid, bone, maximum mediolateral width	~148.9 [L], ~142.4 [R]	167.8+ [R]
Ectopterygoid, primary pneumatic recess, maximum mediolateral width	~46.3 [L], 50.1 [R]	~27.9 [R]
Epipterygoid, maximum dorsoventral height	128.3+ [L]	-
Epipterygoid, maximum rostrocaudal length	31.3+ [L]	-
Dentary, bone, maximum rostrocaudal length	487.6+ [L], 604.5+ [R]	558.3+ [R]
Dentary, dorsoventral height at the third alveolus	~93.3 [L], 100.6 [R]	115.1 [R]
Dentary, maximum dorsoventral height	144.2+ [L], 158.8+ [R]	~184.0 [R]
Dentary, tooth row, maximum rostrocaudal length	~401.7 [L], ~416.0 [R]	~423.0 [R]
Surangular, bone, maximum rostrocaudal length	396.2+ [L], 436.0+ [R]	366.6+ [L], 505.5 [R]
Surangular, bone, dorsoventral height through caudal surangular foramen	147.7 [L], 148.4 [R]	~175.0 [R]
Surangular, bone, maximum dorsoventral height	160.2 [L], 167.6+ [R]	~206.0 [L]
Angular, bone, maximum rostrocaudal length	284.9+ [L], 154.5 [R]	364.1 [R]
Angular, bone, maximum dorsoventral height	~68.5 [L]	~77.9 [R]
Prearticular, maximum rostrocaudal length	188.0+ [L], 191.0+ [R]	-
Prearticular, midshaft, mediolateral width	33.8 [L], ~33.1 [R]	-
Splenial, bone, maximum rostrocaudal length	272.9+ [L], 383.7+ [R]	299.9+ [L]
Splenial, bone, maximum dorsoventral height	107.6+ [L], 138.8+ [R]	164.1 [L]
Splenial, rostral mylohyoid foramen, maximum rostrocaudal length	57.9 [L], ~63.0 [R]	75.3 [L]
Splenial, rostral mylohyoid foramen, maximum dorsoventral height	29.1 [L], 32.5 [R]	29.7 [L]
Intercoronoid, bone, maximum rostrocaudal length	206.4+ [L]	-

16. Table S2. Measurements of the vertebrae of the paratype specimen (MOR 1130) of *Daspletosaurus horneri* sp. nov. All measurements are in millimeters. Abbreviations: L, left; R, right.

Vertebra	Anteroposterior centrum length	Minimum centrum width	Dorsoventral height of anterior surface of centrum	Mediolateral width of anterior surface of centrum	Dorsoventral height of posterior surface of centrum	Mediolateral width of posterior surface of centrum	Dorsoventral height of spinous process from the dorsum of the transverse process	Minimum anteroposterior width of the spinous process
Cervical 4	~57.4	88.3	83.7	106.1	86.7+	121.9+	130.9	24.7
Cervical 7	88.2+	~108.9	67.0+	~113.0	105.8+	128.4+	~148.1	17.1
Cervical 8	92.7	104.7	94.8	~128.0	120.8	127.1+	-	24.6
Dorsal A	76.4+		138.7+	134.3	125.4+	146.0	144.8	~67.9
Dorsal B	62.7+		127.4+	~142.4	97.9+	124.6+	-	-
Dorsal C	-		-	-	-	-	147.5	69.8
Caudal A	~140.6	79.5+	143.3	143.3	136.2	130.7	-	-
Caudal B	-	-	-	-	-	-	181.3	68.4
Caudal C	-	-	-	-	-	-	116.5+	68.7
Caudal D	142.1	46.7+	130.7	110.1	131.3	90.1	147.4	~67.7
Caudal E	-	-	-	-	-	-	~111.5	70.4
Caudal F	-	-	-	-	-	-	~124.0	77.4
Caudal G	-	-	-	-	-	-	93.4	75.1
Caudal H	150.0	35.2+	112.5	106.0	111.0	93.5	-	-
Caudal I	142.1	34.7+	~106.2	99.9	~109.1	86.5+	-	-
Caudal J	145.9	32.5+	113.7	83.7	105.8	70.7	-	-
Caudal K	124.6	~56.2	60.9	~91.6	49.1	73.4+	-	-
Caudal L	74.4	27.8	22.9	29.8	27.7	19.8+	-	-
Caudal M	45.2	~14.4	13.1+	17.9	12.3	15.3	-	-

17. Table S3. Measurements of the appendicular skeleton of the type (MOR 590) and paratype specimen (MOR 1130) of *Daspletosaurus horneri* sp. nov. All measurements are in millimeters. Abbreviations: L, left; R, right.

Measurement	MOR 590	MOR 1130
Humerus, proximodistal length	300.8+ [L]	-
Humerus, proximal mediolateral width	77.2+ [L]	-
Humerus, distal mediolateral width	39.0 [L]	-
Femur, proximodistal length	875.0 [L]	802.7+ [L, R]
Femur, mediolateral width at midheight	107.2 [L]	-
Femur, anteroposterior length at midheight	99.8+ [L]	-
Femur, midshaft circumference	328.0 [L]	-
Femur, proximal mediolateral width	266.0 [L]	-
Femur, distal mediolateral width	186.0 [L]	227.7 [R]
Femur, distal anteroposterior length through medial condyle	176.2 [L]	222.5 [R]
Tibia, proximodistal length	820.0 [L]	920.0 [R]
Tibioastragalus, proximodistal length	840.0 [L]	-
Tibia, mediolateral width at midheight	111.5+ [L]	136.0 [R]
Tibia, anteroposterior length at midheight	65.0+ [L]	83.2 [R]
Tibia, midshaft circumference	~285.0 [L]	345.0 [R]
Tibia, proximal mediolateral width	132.2 [L]	~159.8 [R]
Tibia, proximal anteroposterior length	194.0 [L]	235.3 [R]
Tibia, distal mediolateral width	237.0 [L]	196.0+ [R]
Tibia, distal anteroposterior length	-	~80.3 [R]
Fibula, proximodistal length	710.0 [L]	740.8+ [R]
Fibulocalcaneum length	781.0 [L]	775.0+ [R]
Fibula, mediolateral width at midheight	~29.7 [L]	38.0 [R]
Fibula, anteroposterior length at midheight	40.2 [L]	~42.1 [R]
Fibula, proximal mediolateral width	44.2 [L]	-
Fibula, proximal anteroposterior length	126.3 [L]	166.3+ [R]
Fibula, distal mediolateral width	28.7 [L]	42.3 [R]
Fibula, distal anteroposterior length	53.2 [L]	63.2 [R]
Astragalus, mediolateral width across condyles	202.4+ [L]	-
Astragalus, dorsoventral height of ascending process	160.8 [L]	-
Calcaneum, maximum mediolateral width of condylar portion	30.8 [L]	38.0 [L], 29.6 [R]
Calcaneum, maximum anteroposterior length	90.9 [L]	101.1 [L], 97.2 [R]
Calcaneum, maximum dorsoventral height	77.3 [L]	96.3 [L], 104.1 [R]
Distal tarsal III, anteroposterior length	56.6 [L]	75.9 [R]
Distal tarsal III, mediolateral width	66.3 [L]	85.7 [L]
Distal tarsal IV, anteroposterior length	60.0 [L]	68.2+ [L]
Distal tarsal IV, mediolateral width	87.4 [L]	89.2 [L]
Metatarsal II, proximodistal length	461.8 [L]	480.0 [R]
Metatarsal II, mediolateral width at midheight	63.0 [L]	68.4 [R]
Metatarsal II, anteroposterior length at midheight	75.9 [L]	75.3 [R]
Metatarsal II, midshaft circumference	220.0 [L]	239.0 [R]
Metatarsal II, proximal mediolateral width	121.5 [L]	140.8 [R]
Metatarsal II, proximal anteroposterior length	126.2 [L]	144.7 [R]
Metatarsal II, distal mediolateral width	81.1 [L]	91.3 [R]
Metatarsal II, distal anteroposterior length	86.2 [L]	85.7 [R]
Metatarsal III, proximodistal length	510.8 [L]	-
Metatarsal III, maximum mediolateral width	64.8 [L]	-

Metatarsal III, anteroposterior length at midheight	-	-
Metatarsal III, midshaft circumference	-	-
Metatarsal III, proximal mediolateral width	-	-
Metatarsal III, proximal anteroposterior length	-	-
Metatarsal III, distal mediolateral width	86.3 [L]	-
Metatarsal III, distal anteroposterior length	78.8 [L]	-
Metatarsal IV, proximodistal length	475.1 [L]	530.3 [R]
Metatarsal IV, mediolateral width at midheight	50.6 [L]	50.8 [R]
Metatarsal IV, anteroposterior length at midheight	73.1 [L]	60.7 [R]
Metatarsal IV, midshaft circumference	200.0 [L]	230.0 [R]
Metatarsal IV, proximal mediolateral width	102.4 [L]	126.0 [R]
Metatarsal IV, proximal anteroposterior length	98.9 [L]	102.1 [R]
Metatarsal IV, distal mediolateral width	64.0 [L]	67.1 [R]
Metatarsal IV, distal anteroposterior length	79.6 [L]	93.4 [R]
DII-1, anteroposterior length	141.5 [L]	-
DII-1, minimum mediolateral width	49.5 [L]	-
DII-1, proximal mediolateral width	78.5 [L]	-
DII-1, proximal dorsoventral height	89.8 [L]	-
DII-1, distal mediolateral width	65.0 [L]	-
DII-1, distal dorsoventral height	53.7 [L]	-
DII-2, anteroposterior length	99.5 [L]	-
DII-2, minimum mediolateral width	~44.1 [L]	-
DII-2, proximal mediolateral width	61.8 [L]	-
DII-2, proximal dorsoventral height	53.8 [L]	-
DII-2, distal mediolateral width	50.5 [L]	-
DII-2, distal dorsoventral height	42.1 [L]	-
DII-3, anteroposterior length	88.1+ [L]	-
DII-3, proximal dorsoventral height	38.0+ [L]	-
DII-3, proximal mediolateral width	36.0+ [L]	-
DIII-1, anteroposterior length	129.4 [L]	-
DIII-1, minimum mediolateral width	~50.0 [L]	-
DIII-1, proximal mediolateral width	85.4 [L]	-
DIII-1, proximal dorsoventral height	82.6 [L]	-
DIII-1, distal mediolateral width	76.3 [L]	-
DIII-1, distal dorsoventral height	54.1 [L]	-
DIII-2, anteroposterior length	92.3 [L]	112.7 [L]
DIII-2, minimum mediolateral width	44.4 [L]	47.2 [L]
DIII-2, proximal mediolateral width	68.5+ [L]	~77.4 [L]
DIII-2, proximal dorsoventral height	56.4 [L]	57.3 [L]
DIII-2, distal mediolateral width	62.8 [L]	67.7 [L]
DIII-2, distal dorsoventral height	40.5 [L]	43.9 [L]
DIII-3, anteroposterior length	74.9 [L]	85.2 [R]
DIII-3, minimum mediolateral width	37.8 [L]	47.0 [R]
DIII-3, proximal mediolateral width	56.6 [L]	71.6 [R]
DIII-3, proximal dorsoventral height	40.2 [L]	47.3 [R]
DIII-3, distal mediolateral width	48.9 [L]	59.9 [R]
DIII-3, distal dorsoventral height	35.6 [L]	39.6 [R]
DIII-4, anteroposterior length	99.4+ [L]	-
DIII-4, proximal dorsoventral height	42.5+ [L]	-
DIII-4, proximal mediolateral width	33.0 [L]	-
DIV-1, anteroposterior length	98.5 [L]	-
DIV-1, minimum mediolateral width	48.4 [L]	-
DIV-1, proximal mediolateral width	67.4 [L]	-

DIV-1, proximal dorsoventral height	98.9 [L]	-
DIV-1, distal mediolateral width	62.9+ [L]	-
DIV-1, distal dorsoventral height	52.1 [L]	-
DIV-2, anteroposterior length	~79.1 [L]	57.4 [L]
DIV-2, minimum mediolateral width	53.2 [L]	63.9 [L]
DIV-2, proximal mediolateral width	65.0 [L]	82.4 [L]
DIV-2, proximal dorsoventral height	53.1 [L]	57.4 [L]
DIV-2, distal mediolateral width	~57.8 [L]	70.1 [L]
DIV-2, distal dorsoventral height	44.6 [L]	48.0 [L]
DIV-3, anteroposterior length	59.2 [L]	-
DIV-3, minimum mediolateral width	47.3 [L]	-
DIV-3, proximal mediolateral width	54.3+ [L]	-
DIV-3, proximal dorsoventral height	44.1 [L]	-
DIV-3, distal mediolateral width	50.6 [L]	-
DIV-3, distal dorsoventral height	35.1 [L]	-
DIV-4, anteroposterior length	45.7+ [L]	-
DIV-4, minimum mediolateral width	39.3 [L]	-
DIV-4, proximal mediolateral width	43.7 [L]	-
DIV-4, proximal dorsoventral height	34.5+ [L]	-
DIV-4, distal mediolateral width	44.4 [L]	-
DIV-4, distal dorsoventral height	29.4 [L]	-
DIV-5, anteroposterior length	77.5+ [L]	-
DIV-5, proximal dorsoventral height	39.0 [L]	-
DIV-5, proximal mediolateral width	31.9 [L]	-
